# High‐Performance Recyclable Polyester Elastomers Through Transient Strain‐Stiffening

**DOI:** 10.1002/adma.202416674

**Published:** 2025-04-16

**Authors:** Chang Gao, Kam C. Poon, Matilde Concilio, Thomas Zinn, Georgina L. Gregory, Charlotte K. Williams

**Affiliations:** ^1^ Chemistry Research Laboratory Department of Chemistry University of Oxford Oxford OX1 3TA United Kingdom; ^2^ Diamond Light Source Harwell Science and Innovation Campus Didcot OX11 0DE United Kingdom

**Keywords:** block copolymer, high‐performance, mechanical properties, polycaprolactone, polyester, sustainable polymer, thermoplastic elastomers

## Abstract

Polyester thermoplastic elastomers are promising sustainable materials but their mechanical properties need improvement, in particular, attempts to increase strength often result in compromised elasticity. Strong and tough elastomers are known but require complex polymer formulations together with control over cross‐linking or crystallinity, both of which challenge recycling. Here, the introduction of transient strain‐stiffening approaches into fully amorphous structures show both strengthening and toughening of elastomers while conserving recyclability. The new amorphous block polyester elastomers are prepared by controlled polymerization methods using commercial monomers. The block polymers comprise a central poly(ɛ‐caprolactone‐*co*‐ɛ‐decalactone) block flanked by poly(cyclohexene oxide‐*alt*‐phthalate) blocks. Elastomer thermomechanical properties are tuned by varying ratios of ɛ‐caprolactone to ɛ‐decalactone within the mid‐block to access materials with excellent mechanical properties. The best elastomers feature 30–50 wt.% polycaprolactone and exhibit tensile strengths up to 40 MPa, elongations at break above 2000%, with excellent elastic recovery (>90%). These materials exhibit strain‐induced crystallization and outperform current commercial elastomers, entering a new region of tensile mechanical property space. They have service temperature ranges from −60 to 140 °C and high temperature stability (≥300 °C), with wide thermal (re)processing windows. These new polyester elastomers also show high resistance to creep, humidity resistance, and excellent recyclability.

## Graphical abstract

Transient strain‐crystallization simultaneously strengthens and toughens block polyester elastomers while conserving recyclability. Using controlled polymerization catalysis and commercial monomers, block polyester elastomers outperform current commercial elastomers, entering a new region of tensile mechanical property space.

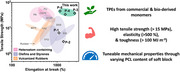

## 1 Introduction

Designing more sustainable polymers is essential to address growing wastes and unacceptable carbon dioxide (CO_2_) emission levels.^[^ ^1^ ^]^ Since the majority of CO_2_ emissions arise during monomer‐polymer manufacturing cycles, it is a priority to ensure all recoverable polymer wastes are recyclable. Indeed, recycling is the only CO_2_‐reduction strategy that falls within other planetary boundaries, for example, land use, critical elements, and climate change.^[^ ^2^ ^]^ One consequence is that the many classes of material properties delivered by polymers need to be reconsidered through the optimization of products for recycling.^[^ ^3^ ^]^

Thermoplastic elastomers (TPEs) are widely used in construction, transportation, household goods, electronics, and other sectors.^[^ ^4^ ^]^ With varying applications as sealants, pipes, vehicle interiors, tires, shoe soles, sports equipment, catheters, and cables, their global market is predicted to reach 5.55 mT by 2026.^[^ ^5^ ^]^ Currently, most elastomers are derived from fossil feedstocks, and whilst their properties are optimized for thermo‐mechanical performances, end‐of‐life waste management is often less clearly considered. In particular, the highest‐performance commercial elastomers, with tensile strengths of 40 to 60 MPa, are generally comprised of heteroatom‐containing (co)polymers. In these cases, the elastomer strength arises from inter‐chain interactions including crystallinity (e.g. polyamides and copolyester elastomers), hydrogen bonding (e.g.polyurethanes and polyamides), or permanent cross‐linking (e.g. vulcanized rubbers). The need to control such interactions across length scales means that these materials are both challenging to process and very difficult to recycle using either thermal reprocessing (mechanical recycling) or depolymerization techniques (chemical recycling).^[^ ^6^ ^]^ One interesting approach to the delivery of high‐performance elastomers would be to exploit transient inter‐chain alignments, for example, strain‐induced crystallization (SIC) which is known in natural rubber.^[^ ^7^ ^]^ The benefits could be that crystallinity would only be temporary, with materials being in a fully amorphous state both before and after the application of stress, thereby enabling recycling without the need to control crystallization rates, crystallite sizes, or other interchain connections.

Block copolymers are an important class of elastomers, including high‐volume products like poly(styrene‐b‐isoprene‐b‐styrene) or poly(styrene‐b‐butadiene‐b‐styrene) (SIS/SBS) or polyolefin elastomers (POE), but these structures tend to deliver lower overall tensile strengths compared to the heteroatom containing polymers. These block copolymers have physical cross‐linking, rather than permanent chemical bonds, which arise from phase separation of the blocks. They often show triblock copolymer structures, where the outer hard blocks comprise a polymer with a glass transition temperature (*T*_g_) or melting temperature (*T*_m_) above room temperature and the central soft block has a *T*_g_ or *T*_m_ below room temperature. Microphase separation of the hard and soft blocks results in an elastomer network with hard domains, which act as physical crosslinks for the soft matrix.^[^ ^4^ ^]^ The material elasticity results from the soft blocks, whereas, the hard block domains bestow mechanical strength and elastic recovery. Thermoplastic elastomers are attractive alternatives to thermoset or vulcanized rubbers with physical rather than chemical crosslinking and can, therefore, be mechanically recycled.^[^ ^8^ ^]^ Design of high‐performing elastomers with high tensile strengths, without a compromise in elasticity or toughness is a long‐standing challenge.^[^ ^9^ ^]^

Hillmyer and co‐workers have pioneered many block polyester‐based elastomers, comprising semi‐crystalline poly‐L‐lactide (PLLA) as the hard blocks combined with innovative soft‐block polymers using monomers from biomass feedstocks.^[^ ^10^ ^]^ For example, elastomeric polyesters were reported using ɛ‐decalactone (from castor oil), menthide (from mint), and methyl‐ɛ‐caprolactone (cresols from lignin bio‐oil). An attraction of this approach is the use of commercial semi‐crystalline PLLA (from corn starch) as the hard block since it is also a leading bio‐derived plastic. These materials tend to show equivalent tensile mechanical properties to the styrenic‐type conventional elastomers (SIS/SBS) but do not always meet the same service temperature range, as the upper limit is restricted by the low *T*_g_ of the polylactide hard blocks (≈5560 °C).

To reach higher operating temperatures, semi‐aromatic polyesters could be useful as end‐blocks and a versatile controlled polymerization to produce them is the ring‐opening copolymerization (ROCOP) of epoxides and anhydrides.^[^ ^11^ ^]^ For example, the ROCOP of phthalic anhydride (PA) and cyclohexene oxide (CHO) yields an amorphous semi‐aromatic polyester with a high *T*_g_ up to 140 °C.^[^ ^12^ ^]^ Additionally, CHO can be produced from the self‐metathesis of waste plant oil^[^ ^13^ ^]^ and PA from corn stover.^[^ ^13^, ^14^ ^]^ It would also be beneficial to use a single reactor and catalyst to access both the hard semi‐aromatic polyester blocks and the soft aliphatic polyester blocks. Our prior reports of switchable polymerization catalysis using mixtures of lactone, epoxide, and anhydride, with a [Zn(II)Mg(II)] heterodinuclear catalyst, produced elastomers showing strengths up to 5 MPa, elasticity 1800%, a wide service temperature range from −50 to 140 °C, and potential for recycling.^[^ ^15^ ^]^ The same switchable, one‐pot polymerization method, allows for similar elastomers but with poly(trimethylene carbonate) (PTMC) as the soft block. These showed higher tensile strengths up to 25 MPa with the strain at break exceeding 800%.^[^ ^15f^ ^]^ The improved properties appear to relate to strain‐induced crystallization of PTMC but the change to mid‐block chemistry unfortunately compromises the operating temperature range due to the *T*_g_ of PTMC being −12 to −18 °C. One consideration is whether the mid‐block strain‐induced crystallization approach is generalizable to other chemistries, and if so, whether it can be used to produce elastomers at high strengths and elasticity, along with improved (low) temperature operability.

Polycaprolactone (PCL) would be a very attractive soft‐block material since it is already commercialized^[^ ^16^ ^]^ and could in the future be bio‐sourced.^[^ ^17^ ^]^ It shows desirable thermomechanical properties with a low glass transition temperature (−60 °C) with a high chain entanglement density (*M*_e_ ≈ 3 kg mol^−1^).^[^ ^18^ ^]^ In the molar mass range needed for elastomers, PCL would be semi‐crystalline, with a *T*_m_ of 59–64 °C, and these properties are much less desirable in a soft‐block material. However, its copolymerization with other lactones is known to result in fully amorphous copolyesters, and in some cases, these maintain the attractive low *T*_g_ value.^[^ ^19^ ^]^ For example, triblock copolymers featuring statistical copolymers of ɛ‐CL with cyclic carbonates (TMC) or esters (δ‐VL, ɛ‐DL, and ɛ‐MCL) as the mid‐block in combination with PLLA outer blocks have been reported.^[^ ^10^, ^20^ ^]^ These elastomeric materials show the desired suppression of PCL crystallinity, with low glass transitions (*T*_g_ = −40 to −60 °C), and reached tensile strengths of 10 to 20 MPa and strain at break ranging between 350%–1000%. Very recently, and while finalizing our manuscript, Li and co‐workers reported a PLLA‐PCL‐PLLA pre‐polymer which was reacted with di‐isocyanates to make polyurethanes, some of which showed high tensile strengths up to 50 MPa and elasticities up to 1000%.^[^ ^21^ ^]^ The polyurethane toughening was attributed to a combination of the PLLA permanent crystallinity and transient strain‐induced crystallization attributed to both PLLA and PCL blocks. These findings substantiate the potential for PCL to undergo strain‐induced crystallization, in a similar manner to our 2023 report of TMC elastomers toughened by strain‐induced crystallization.^[^ ^15f^ ^]^ Our objective in the elastomer design is to avoid any permanent crystallinity since it complicates and limits recycling, therefore PCL should be applied with other co‐monomers to deliver amorphous polyesters. In addition, urethane linkages might be best avoided due to the need for toxic di‐isocyanates, specialized processing conditions, and complexities in their recycling.

To summarize quite a few block polyester elastomers are now able to match the mechanical properties of currently used mid‐strength styrenic block polymers (SIS and SBS). However, there are not yet any materials that fully compete with the high‐performances of polyurethanes (PU), copolyester elastomers (COPE), poly(styrene‐ethylene‐butadiene‐styrene) elastomers (SEBS), and polyamides (PA). Competing with these leading commercial elastomers requires new materials science toughening strategies which also enable efficient, low‐energy recycling. We propose that using copolymers of PCL, which is a leading commercial polyester, as the mid‐block could enable transient strain stiffening (strain‐induced crystallization) as a means to produce both strong and resilient elastomers. In order to deliver the right elastomeric properties, we hypothesized that the mid‐block PCL copolyesters should be flanked by semi‐aromatic polyesters (from PA/CHO) since these polymers show high upper glass transition temperatures and should improve the elastomer service temperature range. To accelerate the uptake of the materials, the entire block polyester should be produced using an efficient, one‐reactor process using commercial monomers and a single catalyst.

## 2 Results and Discussion

### 2.1 Copolymerization of ɛ‐CL and ɛ‐DL

A series of statistical copolymers were synthesized through the copolymerization of ɛ‐caprolactone (ɛ‐CL) and ɛ‐decalactone (ɛ‐DL). ɛ‐DL is also a 7‐membered ring, featuring a substituent adjacent to the ester linkage, and was chosen based on its track record to disrupt PCL crystallinity at minimal incorporation of the comonomer.^[^ ^19^ ^]^ Polymerizations were conducted using a [LZnMg(C_6_F_5_)_2_] heterodinuclear catalyst, at 80 °C, with 1,4‐benzenedimethanol (BDM) as an alcohol initiator (Table S1, Supporting Information). The ratio of ɛ‐CL to ɛ‐DL was varied from 1: 3 to 3: 1 to investigate the onset composition for suppression of PCL crystallinity in the copolymers. Analysis of the polymers by SEC showed high molar mass copolymers with *M*_n_ = 80–120 kg mol^−1^ (Figure S1, Supporting Information), these values agreed well with *M*_n_ determined from ^1^H NMR spectroscopy (Figure S2, Supporting Information). Reactivity ratios, *r*_CL _= 5.2 and *r*_DL _= 0.2 were determined by the Meyer‐Lowry model using the [LZnMg(C_6_F_5_)_2_] catalyst (Figure S3, Supporting Information), and are in good agreement with previous studies.^[^ ^10^, ^22^ ^]^ ɛ‐CL polymerizes faster than ɛ‐DL which results in a gradient microstructure with a long initial run of PCL with ɛ‐DL incorporation increasing as the reaction progresses. As a diol initiator was used, the polymer chains are richer in PCL at the center and show greater PDL content at the ends.

Since, PDL is amorphous (*T*_g_ = −52 °C) and PCL is semi‐crystalline (*T*_g_ = −62 °C, *T*_m _= 60 °C), it is expected the thermal properties of the P(CL‐*co*‐DL) polymers will change with composition. Differential scanning calorimetry (DSC) analysis showed the crystallinity of the materials decreased with higher PDL content as expected (Figure S4, Supporting Information). The polymers are semi‐crystalline at 68 wt.% PCL, with a melt at 22 °C. The degree of crystallinity decreases from 40% in the PCL homopolymer to 15% in the copolymer. The onset of crystallinity suppression was observed in polymers with PCL content below 45 wt.%, the crystallinity is fully suppressed and they are fully amorphous. The ability to control crystallinity by varying the content of PCL and PDL is valuable and makes P(CL‐*co*‐DL) an attractive soft block for the elastomers.

### 2.2 Triblock Polyester Synthesis

**Figure 1:**
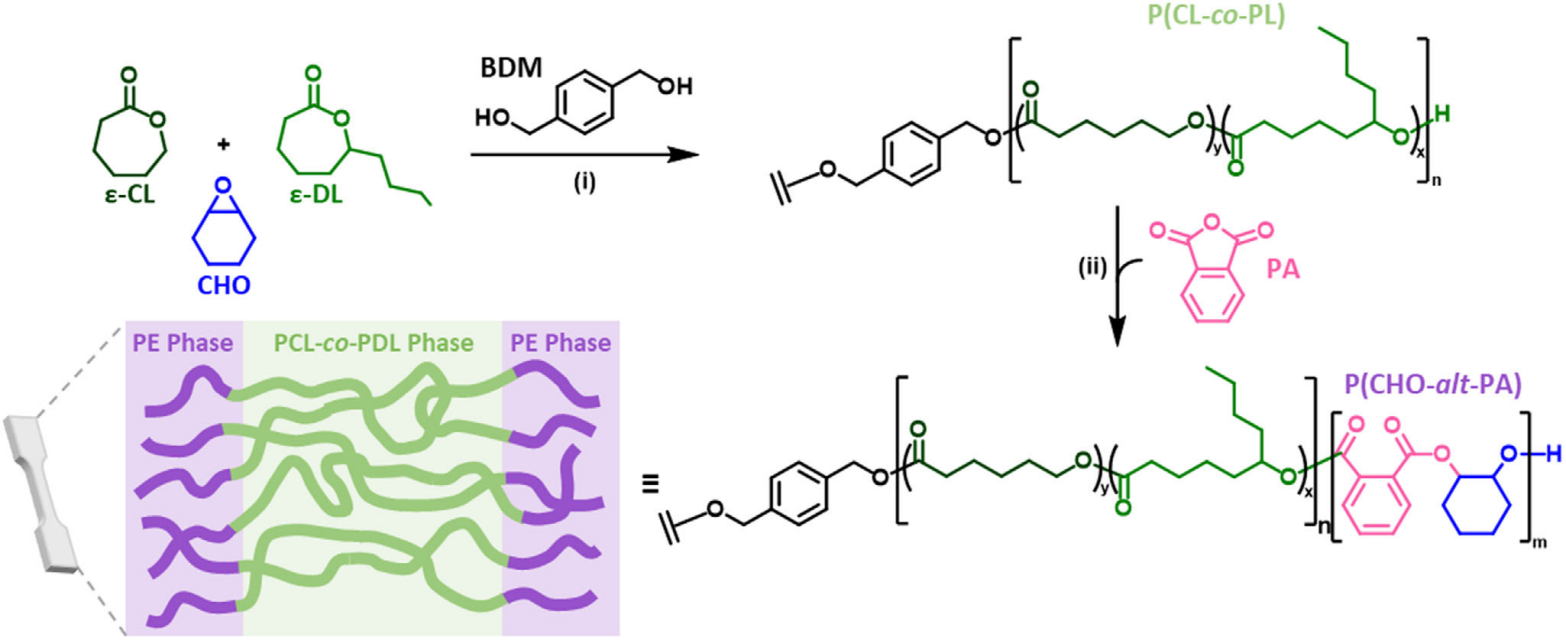
Synthesis of the block polyesters. i) ROP of ɛ‐CL and ɛ‐DL, 80 °C, catalyst = [LZnMg(C_6_F_5_)_2_], where [LZnMg(C_6_F_5_)_2_]_0_: [BDM]_0_: [lactone]_0_: [PA]_0_: [CHO]_0_ = 1: 2: 2000: 384: 577 and [lactone]_0_ = 1.0 m in toluene. ii) ROCOP of CHO and PA, 110 °C, excess of CHO (1.5 equiv. relative to PA).

The synthesis of poly(cyclohexene oxide‐*alt*‐phthalate)‐*b*‐poly(ɛ‐caprolactone‐co‐ɛ‐decalactone)‐*b*‐ poly(cyclohexene oxide‐*alt*‐phthalate) was achieved in a one‐pot procedure using the same [Zn(II)Mg(II)] catalyst (**Figure** **1**). First, the ring‐opening polymerization (ROP) of the two lactones, in the presence of CHO afforded the soft block, and following complete ɛ‐CL/ɛ‐DL consumption, the phthalic anhydride was added. Adding the anhydride triggered a mechanistic switch to ring‐opening copolymerization (ROCOP) of PA and CHO. Through the exploitation of this switchable catalysis, the target triblock polyesters were prepared efficiently and selectively. Both the ROP of ɛ‐CL/ ɛ‐DL, and ROCOP of PA /CHO were catalyzed by the same [LZnMg(C_6_F_5_)_2_] catalyst (Figure S5, Supporting Information). The initiating species, a metal alkoxide, forms from the reaction of the diol with the organometallic catalyst. This highly controlled catalyst results in the production of block polyesters with predictable high molar masses, and monomodal distributions, with high end‐group fidelity, and obviates the requirement for intermediary polymer purification steps.

The statistical copolymerization of ɛ‐CL and ɛ‐DL takes place first, at 80 °C with an initial lactone concentration of 1 M in toluene, to form only poly(ɛ‐caprolactone‐*co*‐ɛ‐decalactone) (P(CL‐*co*‐DL)) soft‐block in high conversion (90%) (Table S2, Supporting Information). Monomer conversion was monitored by ^1^H NMR spectroscopy of a reaction aliquot through a comparison of the relative integrals of the resonances of the ─CH resonances in the monomers (4.27 ppm) with PCL (4.11 ppm) and PDL (4.91 ppm) (Figure S6, Supporting Information). Once the ROP of ɛ‐CL and ɛ‐DL was complete, PA was introduced to grow the alternating polyester end‐capping blocks (P(CHO‐*alt*‐PA)). To improve the PA/CHO reaction rates, the reaction temperature was increased to 110 °C, and the PA/CHO ROCOP reached full conversion (>99%) after 96 h, as confirmed by ^1^H NMR spectroscopy by comparison of the relative integrals of the aromatic resonances in PA (7.99 ppm) and P(CHO‐*alt*‐PA) (7.59 ppm) (Figure S6, Supporting Information). Following the successful synthesis of P(CHO‐*alt*‐PA)‐*b*‐P(CL‐*co*‐DL)‐*b*‐P(CHO‐*alt*‐PA), the reaction was quenched by exposure to air, followed by removal of catalyst and isolation of the polymer by precipitation into methanol. The polymers were solvent cast and dried under vacuum to remove all residual solvent (150 °C, 24 h) to produce transparent and colorless free‐standing films. ^1^H NMR spectroscopy and thermogravimetric analysis (TGA) helped confirm the complete removal of the processing solvents and catalyst. The materials were compression molded (150 °C, 1.0 ton, 60 min) to produce samples suitable for mechanical testing.

The formation of block polymers is supported by SEC analysis of reaction aliquots which shows a steady increase in polymer molar mass (*M*_n_) throughout the reaction whilst retaining a monomodal distribution of moderate dispersity (*Ð* < 1.6). There was no evidence of residual P(CL‐co‐DL) copolymer from the SEC trace (Figure S7, Supporting Information). The ^1^H NMR spectrum of the triblock polymer confirms the outer blocks comprise perfectly alternating PA and CHO, without any ether linkages as evidenced by a 2:1 integration ratio of PA aromatic environments to the CHO methine groups (Figure S6, Supporting Information). Analysis of the carbonyl region of the ^13^C{^1^H} NMR spectra reveals three signals assigned to the P(CL‐co‐DL) soft block (173.5 and 173.4 ppm) and P(CHO‐*alt*‐PA) hard block (166.7 ppm) (Figure S8, Supporting Information). These observations are consistent with block polymer formation without any transesterification block scrambling. Analysis of the chain end‐groups, carried out by ^31^P{^1^H} NMR spectroscopy following the reaction with a phospholane reagent, showed the presence of only P(CHO‐*alt*‐PA) hydroxy end‐groups (146.4 ppm) with no contamination of P(CL‐*co*‐DL) (147.1 ppm) or diblock hydroxy end‐groups (147.1 ppm) (Figure S9, Supporting Information).^[^ ^23^ ^]^ Selective formation of the block polyester is further supported by DOSY NMR spectroscopy through the observation of a single diffusion coefficient for all signals consistent with blocks of P(CHO‐*alt*‐PA) and P(CL‐*co*‐DL) being joined (Figure S10, Supporting Information).

Three block polymers (**P‐2** to **P‐4**) were prepared, where the ratio of PCL:PDL was altered within the soft block while the hard block content was kept constant (≈25 wt.%). These compositions were selected since a hard block weight fraction of ≈25% often yields the body‐centered cubic (BCC) packed spherical or hexagonally packed cylindrical morphologies typically associated with elastomeric performances.^[^ ^15a,e–g^ ^]^ To systematically investigate how the mid‐block polymer influenced the TPE properties its composition was varied (**Table** **1**). As control samples, **P‐1** features only a PDL soft block (27 wt.% hard block) and **P‐5** features only a PCL soft block.^[^ ^15a^ ^]^ All polymers showed high molar mass *M*_n_ ≈ 100 kg mol^−1^, and relatively narrow dispersity (1.3 ≤ *Ð* ≤ 1.6). In all syntheses, monomer conversions were high (>90% lactone conversion and >99% PA conversion) and reactions were highly selective for triblock polyester formation. The soft block compositions were determined from the relative integrals of proton environments in the ^1^H NMR spectrum for PCL (4.11 ppm) and PDL (4.91 ppm). Comparison of integrals for the P(CL‐*co*‐DL) block resonances (4.11 and 4.91 ppm) to the P(CHO‐*alt*‐PA) resonance (5.14 ppm) gives the relative hard block wt.% (Figure S6, Supporting Information).

**Table 1:** Overview of block polyester characterization data.

Sample	PCL:PDL^a)^ [wt%]	PE^a)^ [wt%]	*M*_n,SEC_ ^b)^ [kg mol^−1^] [*Đ*]^c)^	*T*_g_ [°C]^d)^	*T*_m_ [°C]^d)^	*X* [%]^f)^	*T*_d, 5%_ [°C]^g)^
**P‐1**	0:100	27	106 [1.06]	−42, 146	–	–	306
**P‐2**	23:77	24	110 [1.6]	−55, 143	–	–	310
**P‐3**	48:52	26	135 [1.4]	−57, 132	–	–	312
**P‐4**	70:30	28	74 [1.3]	−62, 120^e)^	–	–	317
**P‐5**	100:0	27	101 [1.5]	−62, 111	53	21	317

^a)^ Determined from ^1^H NMR spectrum of purified samples (Figure S7, Supporting Information). PE = P(CHO‐*alt*‐PA);

^b)^ Estimated by SEC with THF eluent, RI detector, calibrated using PS standards;

^c)^ *M*_w_/*M*_n_;

^d)^ Determined by DSC, second heating curve;

^e)^ Upper glass transition temperature from peak in tan(δ) by DMTA;

^f)^ Degree of crystallinity calculated using the reference enthalpy of fusion for fully crystalline PCL (139.5 J g^−1^);^[^ ^24^ ^]^

^g)^ Thermal degradation is reported as the temperature at which 5% mass is lost by TGA.

### 2.3 Polymer Properties

#### 2.3.1 Thermal Properties

**P‐1** to **P‐4** are all amorphous polymers, and **P‐5** has a melting temperature of 53 °C and a degree of crystallinity of 21%. The copolymer midblocks of **P‐2** and **P‐3** are amorphous and the resulting triblock copolymers are also amorphous. In **P‐4**, the midblock polymer on its own showed a small degree of crystallinity (15%), but in the triblock polyester, it is fully amorphous. This suggests that the crystallinity is suppressed by the introduction of the two end‐capping hard blocks. Upon introduction of the hard blocks in **P‐5**, the degree of PCL crystallinity reduces from 40% in PCL to 20% in the triblock polyester. The suppression or reduction of crystallinity observed in **P‐4** and **P‐5**, respectively, likely results from restricted chain mobility due to confinement and anchoring from the hard domains upon their introduction to the triblock.^[^ ^25^ ^]^

For a successful elastomer, phase separation of the hard and soft domains is essential to achieve the desired mechanical properties. To establish phase separation occurs, thermal characterization by differential scanning calorimetry (DSC) is a good first assessment. Two glass transition temperatures close to the expected values for the constituent blocks were observed by DSC analysis indicating microphase separation into P(CL‐*co*‐DL) and P(CHO‐*alt*‐PA) domains for **P‐1** to **P‐5** (Figure S11, Supporting Information). In all samples, the lower glass transition temperature was between −55 and −62 °C: consistent with values expected for the P(CL‐*co*‐DL) block. A decrease in lower *T*_g_ is observed as PCL content is increased within the soft block from **P‐1** to **P‐5**. The upper glass transition temperatures were broad and more difficult to observe by DSC due to the lower hard block content within the polyesters.

**Figure 2:**
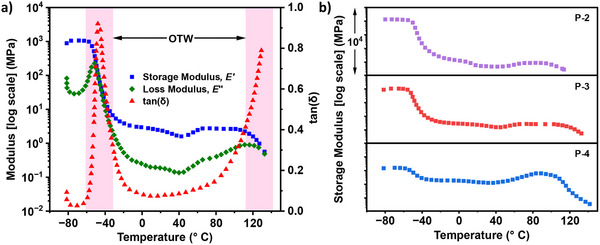
a) Dynamic mechanical thermal analysis (DMTA) temperature sweeps for **P‐3**, OTW = operating temperature window. b) Storage moduli from DMTA temperature sweeps for **P‐2**, **P‐3**, and **P‐4**.

Dynamic mechanical thermal analysis (DMTA) was used to gain a better insight into the thermal transitions taking place (**Figure** **2**a; Figures S12–S14, Supporting Information). Distinct peaks were observed in the tan(δ) profiles (111 to 146 °C) which are clear indicators of the upper *T*_g_ resulting from the hard block. The lower *T*_g_ of the materials was also observed by DMTA by distinct peaks in the tan(δ) profiles (−43 to −48 °C). As a result, the materials have a wide operating temperature window from −60 up to 120 °C. Thermogravimetric analysis (TGA) showed high thermal stability with the onset to decomposition occurring at *T*_d,5%_ ≥ 300 °C; as such there is a reasonably wide processing temperature range for all materials (Figures S15–S18, Supporting Information).

For materials **P‐2** to **P‐4**, there is a sudden increase in storage modulus at ≈50 °C which is indicative of a cold crystallization (Figure 2b). This effect is more pronounced in **P‐4** which has a greater PCL content and shows a larger increase in the storage modulus (E’). This increase in E’ modulus is only present for materials that feature the P(CL‐*co*‐DL) soft block, and is not observed in the DMTA for **P‐1** which shows an extended rubbery plateau. The cold crystallization is not observed by DSC, which could suggest that when materials are placed under strain during the DMTA experiment some PCL crystallization takes place, resulting in the stiffening and increase in E’.

#### 2.3.2 Small‐Angle X‐Ray Scattering

**Figure 3:**
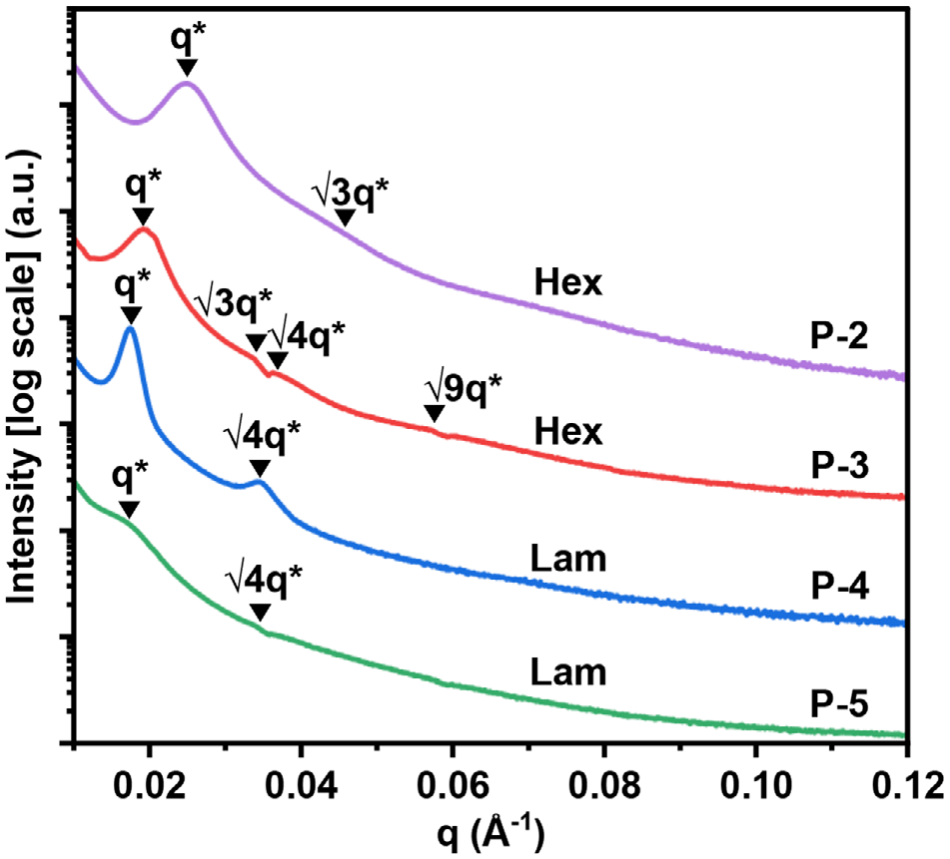
Room‐temperature small‐angle X‐ray scattering (SAXS) profiles showing principal scattering peaks (q*) and higher‐order peaks (*q*/q*).

Small‐angle X‐ray scattering (SAXS) data of films of **P‐2** to **P‐5** support the formation of phase‐separated morphologies with physical cross‐linking. For a good representation of the mechanically tested samples, the films were prepared by solvent casting and annealed at 150 °C, prior to SAXS (**Figure** **3**; Table S3, Supporting Information). In all samples, the SAXS experiments show a principal scattering peak (q*) and some evidence of higher‐order peaks (*q*/q*). The lack of long‐range translational order is common for triblock polymers and commonly observed when χ is low.^[^ ^26^ ^]^ The domain size, calculated from the position of the principal scattering peak (*d* = 2π/q*), ranges between 25–37 nm. **P‐2** and **P‐3** exhibit a principal scattering peak (q*) and higher‐order diffraction peaks (*q*/q* at $\sqrt{3\;}$, $\sqrt{4\;}$) which is consistent with the formation of hexagonally packed cylindrical morphologies. **P‐4** shows the formation of a lamellar phase, indicated by *q*/q* at $\sqrt{4\;}$ with a domain spacing of 35 nm. **P‐5** shows a principal scattering peak corresponding to a domain spacing of 37 nm, with weak further long‐range ordering. Crystalline domains in **P‐5** could cause more restricted mobility of the chains which inhibits long‐range phase separation. A weak diffraction peak at 2q* is tentatively assigned and could correspond to a lamellar morphology. The lack of long‐range order in the new triblock polymers could result from the higher overall molecular weight which leads to more limited chain mobility. The higher density of chain entanglements in high *M*_n_ samples slows chain diffusion and phase separation kinetics.^[^ ^4^ ^]^

#### 2.3.3 Mechanical Properties

To evaluate the stress‐strain behavior of the materials and how the soft block composition impacts mechanical properties, uniaxial tensile testing was carried out at a 10 mm min^−1^ strain rate. Dumbbell‐shaped specimens were cut from the polymer films according to ISO 527‐2, specimen type 5B using a cutting press. For each sample, five different specimens were tested and results are reported as the mean value with standard deviations representing the errors. Young's modulus, tensile strength, strain at break, and tensile toughness are reported for each material (**Table** **2**).

**Table 2:** Tensile properties of polyester elastomers P‐1 to P‐5.

Sample	PCL:PDL^a)^ [wt%]	*E*_y_ ^b)^ [MPa]	σ^c)^ [MPa]	ɛ_b_ ^d)^ [%]	*U*_T_ ^e)^ [MJ m^−3^]	ER^f)^ [%]
**P‐1**	0:100	5.0 ± 0.7	6.5 ± 0.2	1097 ± 37	40 ± 2	95 ± 5
**P‐2**	23:77	2.1 ± 0.2	3.0 ± 0.3	1374 ± 39	31 ± 2	95 ± 5
**P‐3**	48:52	7.3 ± 2.0	38.1 ± 1.8	2422 ± 159	345 ± 36	97 ± 4
**P‐4**	70:30	130.6 ± 22.6	34.2 ± 2.5	1506 ± 64	247 ± 24	93 ± 12
**P‐5**	100:0	515.0 ± 60.8	46.2 ± 6.4	1156 ± 101	311 ± 61	62 ± 16

Samples were tested at a 10 mm min^−1^ strain rate.

^a)^ Determined from ^1^H NMR spectrum of purified samples (Figure S6, Supporting Information);

^b)^ Young's modulus;

^c)^ Tensile strength;

^d)^ Strain at break;

^e)^ Tensile toughness (area under the stress‐strain curve). Mean values ± std. dev. from measurements conducted independently on five specimens;

^f)^ Elastic recovery determined from cyclic tensile testing to 200% strain, mean values ± std. dev. over ten hysteresis cycles.

**Figure 4:**
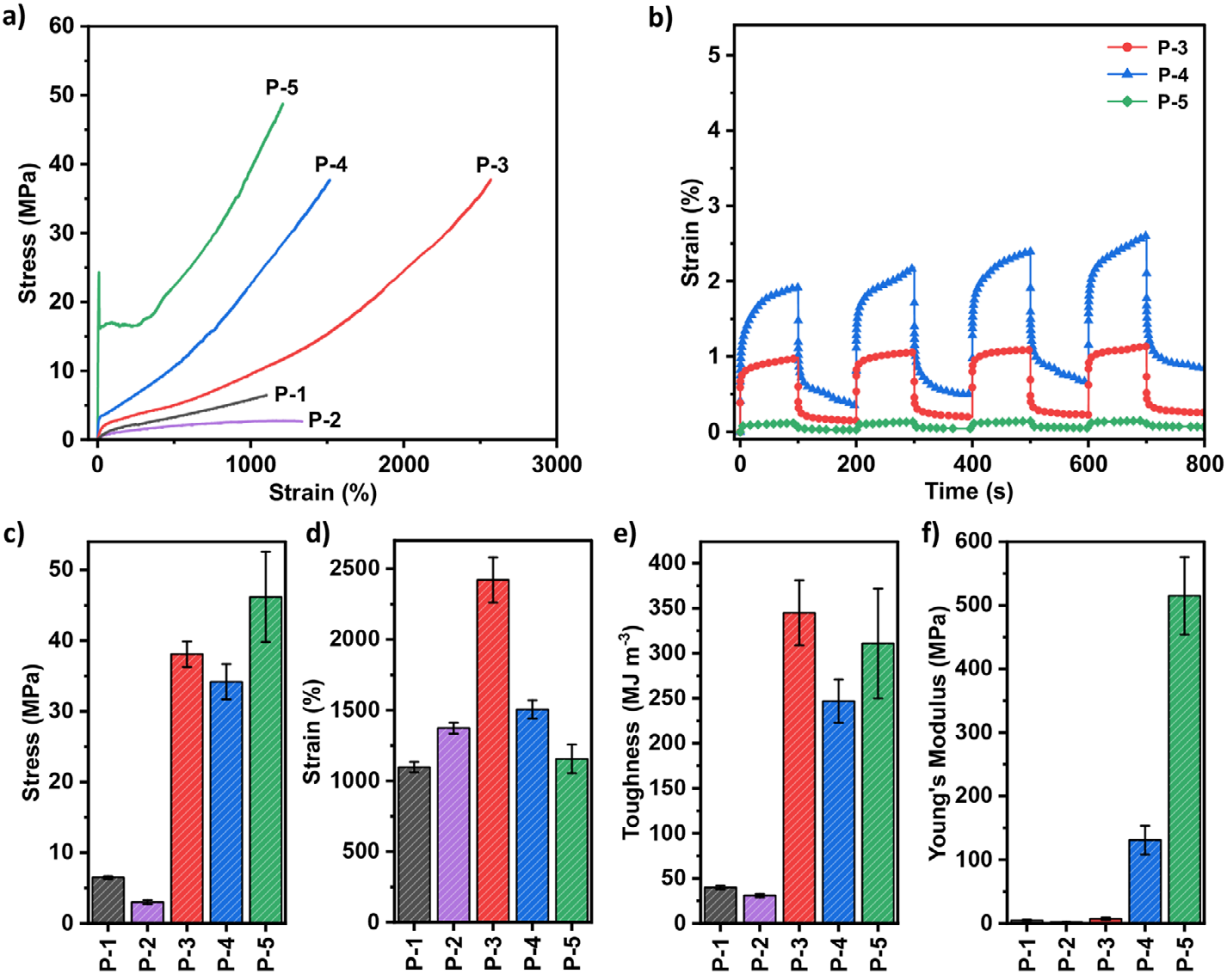
a) Tensile stress‐strain curves (representative of five repeats). b) Creep‐recovery experiments for lead samples **P‐3** to **P‐5**. Comparison of c) tensile strength, d) strain at break, e) tensile toughness, and f) Young's modulus for materials **P‐1** to **P‐5**.

The material's tensile properties depend on the relative PCL content within the soft block. Tensile strengths from 1–40 MPa and elongations at break from 1100%–2400% were achieved across the sample series (Table 2, **Figure** **4**a). **P‐2** to **P‐4**, which feature the P(CL‐*co*‐DL) soft block, are elastomers and exhibit linear stress‐strain relationships with no obvious yield point. **P‐1**, where the soft block consists only of PDL, is a low‐strength elastomer. Introducing a small quantity of PCL into the soft block in **P‐2** (23 wt.%) resulted in a material with comparable mechanical properties to **P‐1**.^[^ ^15a^ ^]^ **P‐2** showed a slight decrease in tensile strength with lower Young's modulus but reached a higher strain at break compared to **P‐1**. Increasing the PCL:PDL ratio to 48:52 in **P‐3** significantly enhanced the overall mechanical properties of the elastomer. **P‐3** showed a tensile strength of 38 MPa (vs. 6 MPa for **P‐1**), strain at break of 2422% (vs 1097% for **P‐1**). It showed a 9‐fold increase in tensile toughness compared to **P‐1**, increasing from 40 to 345 MJ m^−3^. **P‐3** shows a significant improvement in tensile strength with an equivalent increase in elongation at break. Further increasing the PCL content of the soft block in **P‐4** stiffened the material even more, compared with the elastomer featuring only PDL as the soft block, and still exhibited a strain at a break of 1506% and high tensile strength of 34.2 MPa. This means **P‐4** has a high tensile toughness of 247 MJ m^−3^. Introducing 48 to 70 wt.% of PCL into the soft block results in optimal properties and simultaneously improves the tensile strength, extensibility, and toughness compared to **P‐1** with a PDL midblock.

The stress‐strain data for **P‐3** and **P‐4** show a gradual increase in strengths occurring at 1500% and 700% strain, respectively. These are accompanied by a rapid increase in modulus which significantly enhances the mechanical performance. We attribute this behavior to transient strain‐induced crystallization of the PCL segments in the soft block (vide infra). This uptick in the stress‐strain curve is typical of materials with SIC behavior.^[^ ^15f^ ^]^ The crystalline domains formed can act as barriers to deformation, increasing the strength of the elastomers as they are being stretched. The stress‐strain data of **P‐5**, where the soft block comprises only PCL, is typical of a tough and ductile plastic. A clear yield point is observed at 24 MPa and 9% strain, indicating plastic deformation, followed by significant strain‐hardening. The crystallinity in **P‐5** is responsible for the strain‐hardening as the crystallites act as physical barriers to chain dislocation movement. **P‐5** achieves the highest tensile strength of 46 MPa and still shows a very high strain at a break of 1156% and tensile toughness of 311 MJ m^−3^. Across the series, the polymers show increasing Young's modulus from 2 MPa to 515 MPa as more PCL is incorporated into the midblock of the materials. It is clear that using PCL in the soft block improves their mechanical properties including increases in tensile strength, elongation at break, and toughness.

Rheological creep‐recovery samples were conducted on lead samples **P‐3**, **P‐4**, and **P‐5** (Figure 4b). For high‐performance applications, materials must be able to withstand mechanical creep, the permanent deformation or distortion over time under applied stress. The experiments were carried out at 30 °C and at a constant stress of 5 kPa for four intervals lasting 100 s, each followed by 100 s of 0 Pa stress to recover. Out of the elastomers, **P‐3** performed the best, reaching just 1.0% strain which did not increase in subsequent cycles. **P‐4** showed minor creep with a strain of 2.0% that increased to 2.6% in the final cycle. **P‐5** showed a very low strain of only 0.1% which remained unchanged in the following cycles. Overall, all samples showed excellent creep resistance likely due to the presence of PCL crystalline domains in the polymer backbone.

**Figure 5:**
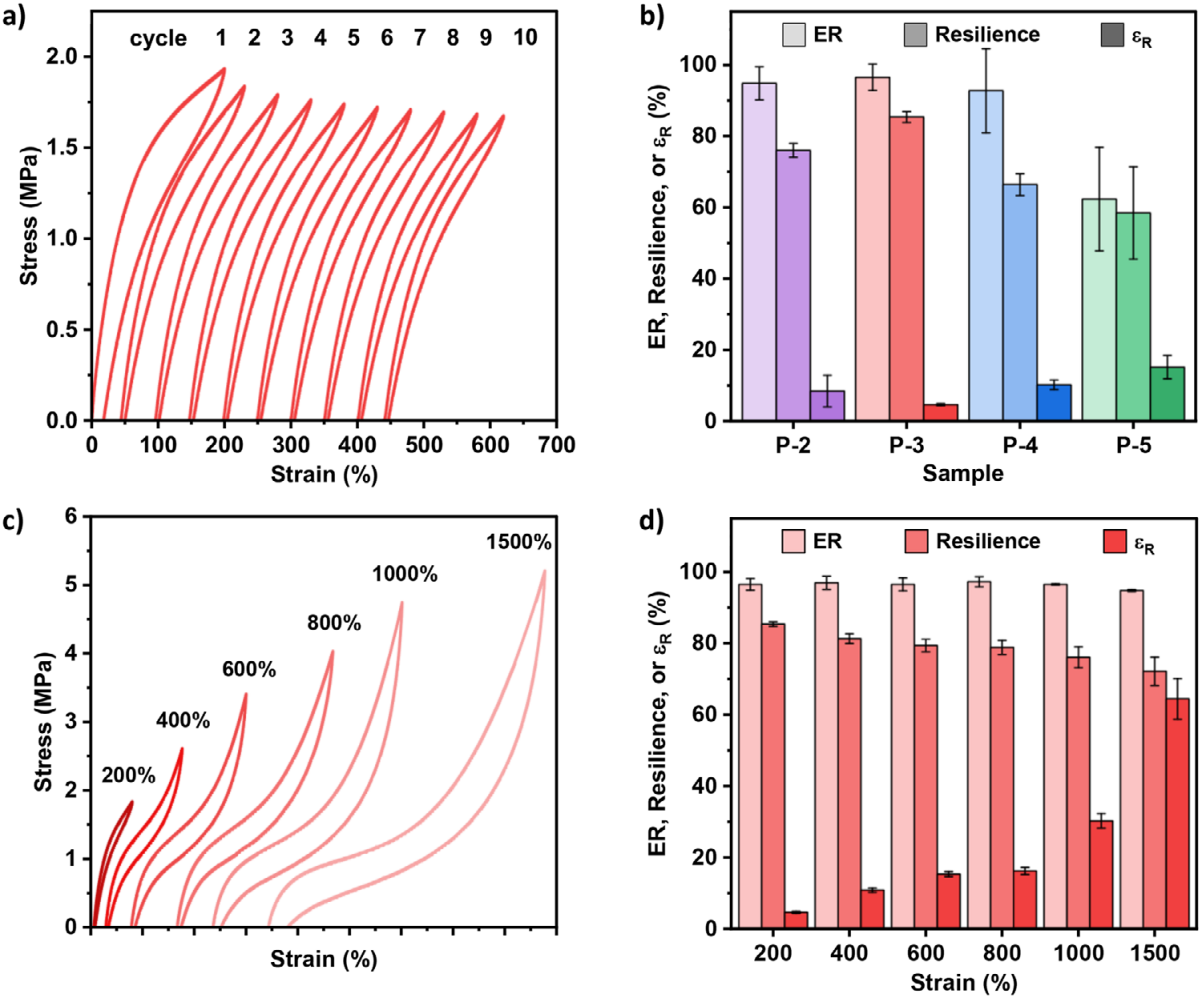
a) Cyclic testing for **P‐3** (200% strain) exhibiting narrow hysteresis (offset by 50% for clarity). b) Comparison of elastic recovery (ER), resilience, and residual strain (ɛ_R_) for **P‐2**, **P‐3**, **P‐4**, and **P‐5**. c) Cyclic tensile testing for **P‐3** to different % strains (offset for clarity). d) Corresponding elasticity properties at different % strains for **P‐3**.

To better assess the elastomeric behavior further testing was conducted. Samples **P‐2** to **P‐5** were subjected to 10 loading and unloading cycles where each sample was stretched to 200% strain and relaxed at a rate of 10 mm min^−1^ to 0% strain (**Figure** **5**a; Figure S20, Supporting Information). In all cases, the first cycle displays greater hysteresis than subsequent cycles due to stress‐induced chain disentanglement within the soft block.^[^ ^27^ ^]^ Elastic recovery, the ability of the material to recover its original dimension after extension, and resilience (the ability of a material to recover energy after deformation) along with residual strain (permanent deformation remaining in the material after extension and removal of applied stress) are important parameters in measuring elastomer performance (Figure S19, Supporting Information). Using the cyclic testing experiments, the elastic recovery (ER), resilience, and residual strain (ɛ_R_) were determined for each material (Figure 5b; Table S4, Supporting Information). **P‐2** to **P‐4** all demonstrate excellent elastomeric properties >90% high elastic recovery, >60% high resilience, and <10% residual strain. **P‐3** was the best performing elastomer, showing the highest elastic recovery (97%) and resilience (85%) with minimal residual strain (5%). The elastomeric performance drops slightly as more PCL is introduced in the soft block in **P‐4**. This suggests a 50:50 ratio of PCL:PDL within the soft block is optimal for achieving the best elastomeric performance. As expected, **P‐5** shows lower recovery and resilience due to the rigid PCL midblock and absence of PDL in the soft block (ER = 62%, resilience = 58%, and ɛ_R_ = 15%). To demonstrate the very high elasticity of **P‐3** cyclic tensile testing experiments were conducted at different strains from 200% to 1500% (Figure 5c,d). At greater strains up to 1500%, hysteresis loss and slightly lower resilience were observed. Even at 800% strain, the residual strain remained low (<20%). At strains ≥1000%, nonetheless, residual strain increases more significantly in the cyclic experiments to ɛ_R_ = 30% and 64%. The drop in resilience and increase in ɛ_R_ during cyclic tests at higher strains is consistent with the proposed SIC and could be rationalized by the retention of small PCL crystallites during the unloading cycle. Despite this, elastic recovery remains exceptionally high (≥95%) for **P‐3** from 200% up to 1500% strain.

### 2.4 Strain‐Induced Crystallization

**Figure 6:**
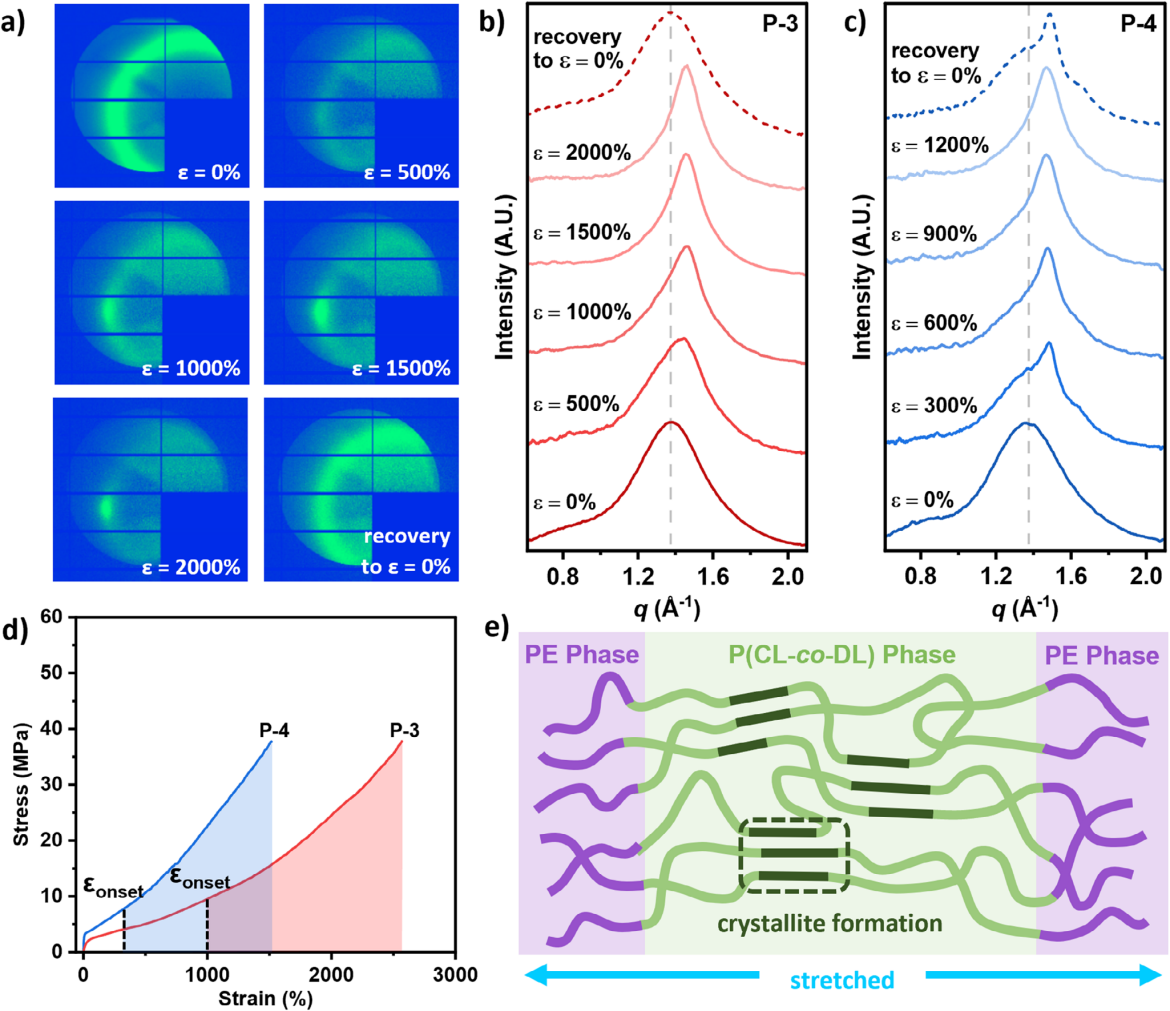
a) Evolution of 2D‐WAXS patterns of **P‐3** recorded at increasing % strain (ɛ) and after recovery to 0% strain. 1D‐WAXS intensity profiles at increasing % strain (ɛ) and after recovery to 0% strain for **b) P‐3** and **c) P‐4**. **d)** Tensile stress‐strain curves for **P‐3** and **P‐4** showing the crystallization onset strain (ɛ_onset_). **e)** Schematic showing strain‐induced crystallization.

In situ wide‐angle x‐ray scattering (WAXS) profiles were collected during tensile testing of materials **P‐1** to **P‐5** to assess the presence of strain‐induced crystallization (**Figure** **6**a; Figure S23–S25, Supporting Information). The samples were uniaxially extended at a 10 mm min^−1^ strain rate while WAXS profiles were collected at increasing increments of strain to study the crystallinity of the sample under strain.

Initially, a broad scattering peak at *q* = 1.37 Å^−1^ is observed in the unstretched samples of **P‐1** to **P‐4** which have PDL containing soft blocks (Figure 6b,c; Figure S26, Supporting Information). This peak relates to chain alignment effects related to the n‐butyl substituents on the PDL backbone, specifically the separation is consistent with intra‐substituent separations between the alkyl groups.^[^ ^28^ ^]^ These amorphous domains are expected to be randomly orientated, consistent with the uniform circular diffraction rings observed in the isotropic 2D‐WAXS pattern at ɛ = 0% (Figure 6a; Figures S23–S25, Supporting Information). For elastomers **P‐1** and **P‐2,** strain‐induced crystallization is not expected due to their limited PCL content and inferior mechanical performance. No differences were observed in the WAXS pattern and the broad peak at *q* = 1.37 Å^−1^ remained unchanged as the materials were strained up to 1200%, these results are consistent with no strain‐induced crystallization occurring (Figure S26, Supporting Information).

The superior mechanical performance of elastomers **P‐3** and **P‐4** is attributed to SIC of PCL segments within the P(CL‐*co*‐DL) soft block. As these materials were strained (up to 1500%), anisotropic WAXS patterns with discrete spots were observed, indicating the formation of orientated crystallites upon stretching (Figure 6a). The broad PDL peak at *q* = 1.37 Å^−1^ overlaps with the expected crystalline reflections at *q* = 1.5 and 1.65 Å^−1^ for PCL (corresponding to the (110) and (200) crystal planes, respectively).^[^ ^29^ ^]^ However, a clear shift to higher *q* is observed in the 1D‐WAXS intensity profiles for **P‐3** and **P‐4** (Figure 6b,c), this corresponds to a decrease in domain spacing which suggests crystalline regions within the polymer form and align along the stretching direction. Narrowing of the scattering peak is also observed at increasing strain, signifying growth in crystallite size with higher crystalline order and decreased contribution from the broad PDL peak. This further demonstrates the formation of orientated PCL crystallites in the stretched samples. The crystallization onset strain (ɛ_onset_) is higher for **P‐3** (>1000%) compared to **P‐4** (>300%), which is expected since **P‐4** has greater PCL content within its soft block (Figure 6d). To test the reversibility of the crystallite formation in **P‐3** and **P‐4**, WAXS measurements were taken after the elastomers were relaxed to 0% strain. For **P‐3,** only a broad peak corresponding to PDL is observed, indicating the sample returns to the amorphous state when relaxed and the SIC is transient. For **P‐4**, the scattering peak shows some preservation of the sharp peak assigned to PCL crystallites, suggesting that there is some residual crystallinity retained after relaxing the sample (Figure 6c). Deconvolution of this peak was possible and shows the contributions from the broad PDL peak at *q* = 1.37 Å^−1^ as well as peaks at *q* = 1.5 and 1.65 Å^−1^ corresponding to PCL crystallites (Figure S27, Supporting Information). The more limited SIC reversibility, in this case, is consistent with the lower elastic resilience of **P‐4** which shows higher residual strain and lower resilience in testing. The tensile‐WAXS of **P‐3** was repeated at a higher strain rate of 50 mm min^−1^ to assess the effect of strain rate on SIC. However, there were no changes in the extent or reversibility of the strain‐induced crystallinity with faster strain rates (Figure S28, Supporting Information).

To examine the effect of stress on the SIC, stress relaxation experiments were conducted where **P‐3** was strained to 1500% and held under constant strain as the decrease in stress was measured over time, and crystallinity was monitored by WAXS (Figure S29, Supporting Information). No changes in the crystallinity of the sample were observed as stress dropped during the relaxation process. This finding is quite consistent with the literature as materials that exhibit SIC are known to show stress relaxation mechanisms dominated by crystallization rather than chain disentanglement at high strains.^[^ ^30^ ^]^ After 3 h of holding at 1500% strain, **P‐3** was recovered to 0% strain and the elastomer returned to the amorphous state shown by the melting of PCL crystallites in the WAXS pattern. This provides further evidence of reversible SIC in **P‐3** and shows crystallization only takes place when materials are placed under high strain. SIC is the key contribution to the strain‐stiffening of elastomers **P‐3** and **P‐4,** which results in toughened elastomers that are capable of reaching high tensile strengths with no sacrifice in elasticity.

In situ tensile‐WAXS measurements of **P‐5**, which feature only PCL as its mid‐block, confirm it is a crystalline material and suggest a random orientation for those crystallites (Figure S30, Supporting Information). As expected in the unstrained (starting) sample, there is no PDL scattering peak at *q* = 1.37 Å^−1^, but rather there are two PCL peaks at *q* = 1.5 and 1.65 Å^−1^, with a shoulder at *q* = 1.55 Å^−1^. Stretching of **P‐5** orientates the existing crystalline domains resulting in anisotropic scattering and slight broadening of the peaks in the WAXS profiles. The strain‐hardening behavior of **P‐5** arises from strain‐induced alignment of crystalline domains which prevents mechanical deformation resulting in a toughened plastic.

### 2.5 Environmental Stability Testing and Polymer Recycling

To confirm the environmental stability of these polymers in humid conditions, the modulus was monitored with increasing relative humidity for the three best performing materials **P‐3**, **P‐4,** and **P‐5** (Figure S31, Supporting Information). Constant values of storage and loss moduli were maintained as the relative humidity increased from 10% to 90% for all three materials. The excellent stability of **P‐3**, **P‐4,** and **P‐5** can be attributed to the hydrophobicity of PCL and PDL which are the majority component of these materials, and ensures the mechanical integrity of the samples remains uncompromised even in the harshest humid environments.

**Figure 7:**
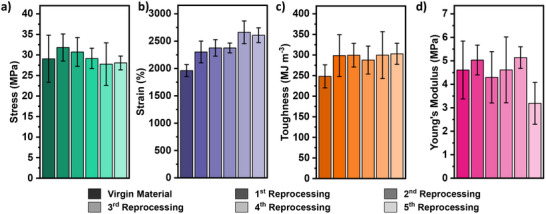
a) Tensile strength, b) Strain at the break, c) Tensile toughness, and d) Young's Modulus for virgin P‐3 after five mechanical reprocessing cycles. Mean values ± std. dev. from measurements conducted independently on five specimens.

Designing polymers to facilitate closed‐loop recycling and reprocessing is crucial to minimize end‐of‐life environmental impacts. The reprocessing temperature was determined by rheological temperature sweep experiments which showed a crossover in G′ and G″, at 150 °C (Figure S32, Supporting Information). Mechanical recyclability of the lead sample, **P‐3**, was investigated by compression molding at 150 °C, 1.0 ton for 30 min over five reprocessing cycles (Figure S33, Supporting Information). This allowed the preparation of recycled films suitable for uniaxial tensile testing which remained transparent and colorless throughout the mechanical reprocessing process. The recycled materials retained the same excellent tensile strengths, strains at the break, tensile toughness, and Young's modulus over the five reprocessing cycles in all cases compared equivalently to the virgin material (**Figure** **7**). Thus, mechanical reprocessing of these high‐performance materials would be used as an efficient means to recycle waste products and manufacturing scraps without degradation or worsening mechanical performance.

## 3 Discussion

**Figure 8:**
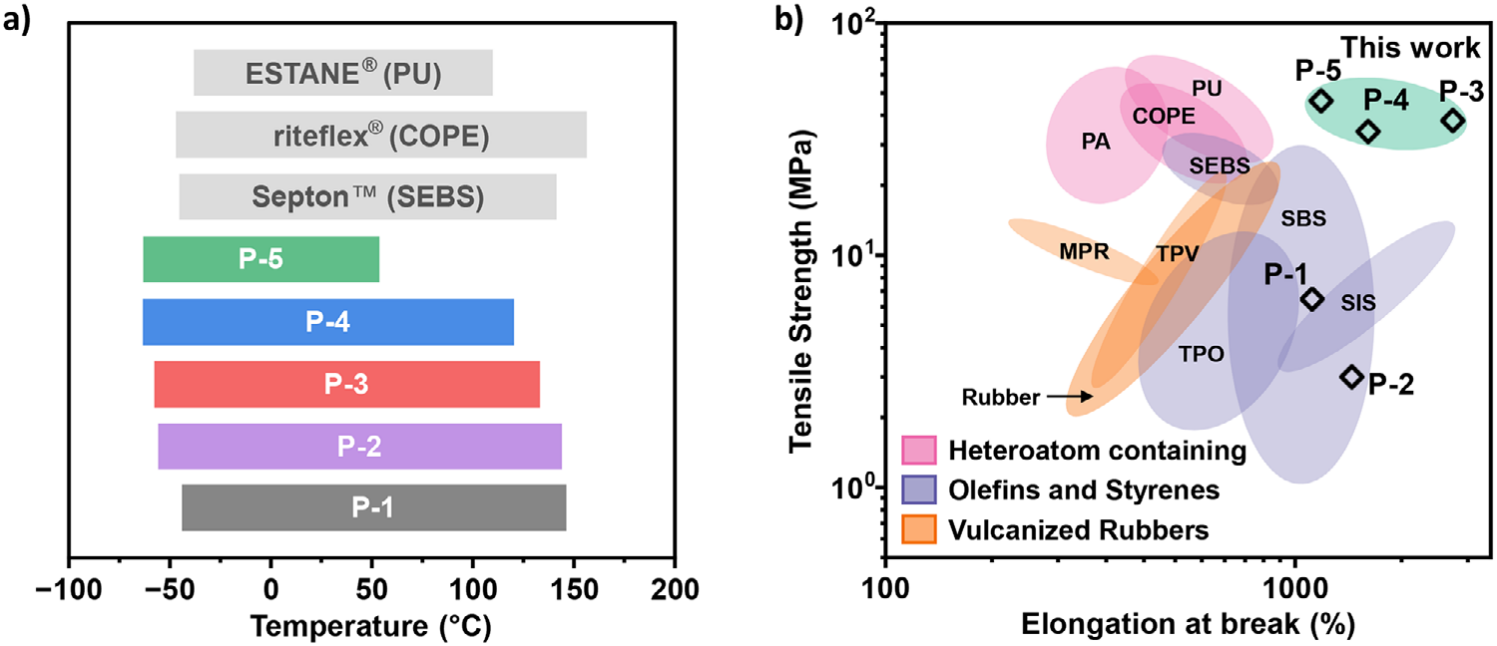
a) Comparison of operating temperature window for **P‐1** to **P‐5** with commercial TPEs. b) Ashby plot produced using mechanical data for commercial TPEs compared with **P‐1** to **P‐5**.^[^ ^32^ ^]^ PU = polyurethane, COPE = copolyester elastomer, PA = polyamide, SEBS = poly(styrene‐b‐ethylene‐co‐butylene‐b‐styrene), SIS = styrene‐isoprene‐styrene, SBS = styrene‐butadiene‐styrene, MPR = melt processable rubber, TPV = thermoplastic vulcanizates, TPO = thermoplastic polyolefin.

Here, a straightforward method to tune the thermomechanical properties of elastomers by predictable changes to block polyester compositions is presented. A range of different materials, from toughened elastomers to ductile plastics, are produced through control of mid‐block PCL content, from 23–100 wt.%. These materials are synthesized via efficient and controlled polymerizations, in one reactor, using a single catalyst (0.05 mol% loading) and at accessible temperatures (80–110 °C). These synthesis conditions contrast with those used to produce currently used styrenic block polymer elastomers, like SIS and SBS, which requires precise control over monomer additions and is energy intensive due to the low temperatures needed.^[^ ^4^ ^]^ The thermal and mechanical properties of these new elastomers are comparable to or outperform the highest‐strength commercial elastomers, like polyurethanes (PU), copolyester elastomers (COPE), and styrenic block polymers (SBS, SIS, & SEBS). Current materials often achieve their strengths, without compromising elasticity, by careful control over intra‐chain crystallinity, hydrogen bonding, or permanent cross‐linking.^[^ ^31^ ^]^ These strategies are effective from a materials science perspective but do create significant challenges for the re‐processing and recycling of the polymers. Thus, there is an incentive to explore fully amorphous block polymer elastomers, since these are much more straightforward to reproducibly recycle, and are strong and resilient. In this work, the new triblock elastomers show exactly such high strengths and elasticities, and the materials are amorphous; they have wide service temperatures from −60 up to 140 °C, which is comparable to or better than today's top‐performing commercial elastomers (**Figure** **8**a). Achieving such high temperature operability has been a very longstanding challenge in the elastomer field and has not proven possible in most cases using PLLA. Further, the current polyesters show a reasonable processing temperature range from 150–300 °C which is compatible with many current methods.

In the literature, the majority of reported polyester elastomers to match the mechanical performances of SIS or SBS styrenic block polymers, with very few examples capable of reaching both high tensile strength and high elongation at break (usually reported at a very high strain rate).^[^ ^10^, ^15^ ^]^ In this series of elastomers, **P‐3** and **P‐4** showed the best mechanical performances, with impressive tensile strengths (>35 MPa) and significantly higher elongation at breaks (>2400%), entering a new region of elastomer property space (Figure 8b). The tensile mechanical and elastomeric behavior of leading commercial elastomers, SEBS (Septon) and PU (Estane) were examined to compare directly with **P‐3** and **P‐4** (Figures S34–S39, Table S5, Supporting Information). Considering their tensile mechanical properties, **P‐3** closely resembles the commercial SEBS, while **P‐4** displays a similar property profile to the PU elastomer. Elastomers **P‐3** and **P‐4** also showed similarly high creep resistance to leading commercial materials, performing comparably to SEBS and PU samples, respectively (Figure S40, Supporting Information). The environmental stability of **P‐3, P‐4**, and **P‐5** were very similar to commercial SEBS and, importantly, all samples showed no change in storage modulus with increasing humidity. Conversely, a loss in mechanical integrity was observed for the commercial PU with increasing humidity (Figure S41, Supporting Information). The phenomena resulting in the latter effect are beyond the scope of this work but likely arise from plasticization/disruption of hydrogen bonding networks at high humidity. The challenge of producing block polymer thermoplastic elastomers that outperform existing petrochemical elastomers has been achieved here using PCL in fully amorphous polymers.

Recycling of these new polyester elastomer materials is a very significant benefit and was achieved at low temperatures (150 °C with compression molding) and over five repeated recycles maintaining the high tensile mechanical and thermal properties. To calibrate the results, high‐performance commercial PU and SEBS elastomers were also mechanically recycled, using similar procedures but at slightly higher temperatures (190 °C). The SEBS retained good mechanical performance in the first three reprocessing cycles but in recycles four and five the samples showed significantly lower stress, elongation at break, and tensile toughness (Figure S42, Supporting Information). The PU recycling performed very poorly, showing a large drop‐off in mechanical performance after the first recycle and a gradual decrease in each subsequent recycling round (Figure S43, Supporting Information). When compared to commercial materials, **P‐3** retains excellent mechanical properties over five rounds of mechanical reprocessing, showing superior mechanical recyclability. The new triblock elastomers can also be processed at lower temperatures which may be beneficial in reducing process energy requirements.

The unusual mechanical performances of **P‐3** and **P‐4** are attributed to strain‐induced crystallization of PCL segments within the soft block portion of the polymer. In situ tensile‐WAXS studies revealed the formation of PCL crystallites at high strain with an ɛ_onset_ of >1000% and >300% for **P‐3** and **P‐4**, respectively. The copolymer soft block has a gradient microstructure where regions of the polymer chain are richer in PCL. Alignment and crystallization of PCL segments occur under stress, giving rise to toughened materials that have high tensile strength without sacrificing extensibility. Signs of crystallite formation were also suggested by the DMTA analysis whereby a sudden increase in storage modulus occurred for materials featuring the P(CL‐*co*‐DL) blocks (**P‐2**, **P‐3,** and **P‐4**); such increases are indicative of a cold crystallization. Strain‐induced crystallization in **P‐3** was shown to be reversible by WAXS which showed melting of PCL crystalline domains upon release of strain. DSC analysis immediately after stretching the sample also showed no evidence of any crystallites (Figure S44, Supporting Information). The transient nature of the crystallinity means the elastomers are fully amorphous before and after stretching, enabling facile reprocessing and recycling of materials, without the need to control the extent of crystallization.

The use of poly(caprolactone) PCL in these elastomers is important in the delivery of the tuneable mechanical properties and in accessing the new region of mechanical property space. Commercial materials (SIS/SBS, SEBS, COPE, PA, and PU), apply many different monomers and polymer chemistries to reach the target mechanical properties. In contrast, there may be advantages to using a more limited monomer palette and controlling compositions, as described here, to cross property space from softer elastomers (**P‐2**) to toughened elastomers (**P‐3** and **P‐4**) and ductile plastics (**P‐5**). Furthermore, the majority component of the highest performing materials is PCL, which is already a commercialized plastic and which now shows high potential in the elastomer sector. The strategy to exploit the beneficial properties of PCL outlined in this work should be amenable to other monomer mixtures, particularly as epoxide/anhydride ROCOP is known to be effective using many (rigid) epoxides and anhydrides.^[^ ^11b^ ^]^ There are also known routes to many such lactones, epoxides, and anhydrides using industrial wastes and biomass which could further improve sustainability.^[^ ^11^, ^33^ ^]^

## 4 Conclusion

Block polyester elastomers are attractive for their controlled synthesis and recyclability. However, they often fail to meet the materials performances of leading commercial elastomers. Here, a new series of block polyesters, comprising poly(caprolactone‐co‐decalactone) mid‐blocks flanked by semi‐aromatic polyester hard blocks, were efficiently prepared from commercial monomers, which are all already used in polymer manufacturing. A range of elastomers with tunable thermomechanical properties were accessed, through varying the polyester composition, which highlights the benefits of the controlled polymerization catalysis. Elastomers with impressive tensile strengths (40 MPa), extensibilities (2500%), creep resistance, and excellent elastic recovery (>90%) were achieved. Using only PCL, as the soft block, resulted in a tough, ductile plastic with a high tensile strength of 46 MPa, a high strain at break of 1156%, and tensile toughness of 311 MJ m^−3^. These materials showed wide service temperature ranges (−60 up to 140 °C) which are comparable to the highest‐performing commercial elastomers. The elastomers were efficiently and effectively mechanically recycled, by compression molding at 150 °C, resulting in recycled products showing the same thermal‐mechanical properties and very good environmental stability. This work demonstrates that starting from just four already commercialized monomers and exploiting controlled polymerization methodologies, it is feasible to cross materials property maps for elastomers. It also highlights the significant potential for polycaprolactone, a leading sustainable plastic, in the elastomer sector. The design strategy of producing fully amorphous elastomers that are toughened by strain‐induced crystallization should be exploited in the future to prepare a wide variety of recyclable plastics and elastomers.

## Conflict of Interest

The authors declare no conflict of interest.

## Supporting information



Supporting Information

## Data Availability

The data that support the findings of this study are available in the supplementary material of this article.
