# Supplementary material for: High‐Performance Recyclable Polyester Elastomers Through Transient Strain‐Stiffening
Source: Adv Mater. 2025 Apr 16;37(27):2416674. doi: 10.1002/adma.202416674 (PMC12243709; doi:10.1002/adma.202416674)
Supplement: Supplementary file 1 — Supporting Information [file ADMA-37-2416674-s001.docx]

Supporting Information:

**High performance Recyclable Polyester Elastomers Through Transient Strain-Stiffening**

Chang Gao, Kam C. Poon, Matilde Concilio, Thomas Zinn, Georgina L. Gregory, & Charlotte K. Williams*

**Table of Contents**

[Experimental Details 5](#_Toc190172898)

[NMR Spectroscopy 5](#_Toc190172899)

[Size Exclusion Chromatography 5](#_Toc190172900)

[Phosphorus End-Group Tests 5](#_Toc190172901)

[Differential Scanning Calorimetry 5](#_Toc190172902)

[Thermogravimetric Analysis (TGA). 5](#_Toc190172903)

[Film Preparation 5](#_Toc190172904)

[Tensile Testing. 5](#_Toc190172905)

[Dynamic Mechanical Thermal Analysis (DMTA) 5](#_Toc190172906)

[Rheology 5](#_Toc190172907)

[Small-angle X-ray Scattering (SAXS). 6](#_Toc190172908)

[Wide-angle X-ray Scattering (WAXS). 6](#_Toc190172909)

[Reagents and Methods 6](#_Toc190172910)

[Synthesis of [LZnMg(C_6_F_5_)_2_] Catalyst 6](#_Toc190172911)

[General Polymerization Procedure. 7](#_Toc190172912)

[Table S1. Composition and thermal characteristics for Poly(CL-co-DL) copolymers (mid-block compositions for block polymers). 8](#_Toc190172913)

[Figure S1. SEC trace (THF eluent, 1 mL min^-1^) for poly(CL-co-DL) copolymers. 8](#_Toc190172914)

[Figure S2. ^1^H NMR (400 MHz, CDCl_3_) spectrum of PCD-76. 9](#_Toc190172915)

[Figure S3. Meyer-Lowry fit to ε-CL/ε-DL copolymerisation data. 10](#_Toc190172916)

[Figure S4. Differential scanning calorimetry (DSC) traces (second heating curve) for poly(CL-co-DL) copolymers. 10](#_Toc190172917)

[Figure S5. Schematic illustrating switch catalysis strategy employed for synthesis of poly(cyclohexene-alt-phthalate)-*b*-poly(ε-caprolactone-*co*-ε-decalactone)-*b*-poly(cyclohexene-alt-phthalate) triblock copolymers 11](#_Toc190172918)

[Table S2. Summary of Polymerisation Conditions and Results 11](#_Toc190172919)

[Figure S6. ^1^H NMR (400 MHz, CDCl_3_) spectrum of P-3. 12](#_Toc190172920)

[Figure S7. SEC trace (THF eluent, 1 mL min^-1^) for (a) PDL block aliquot and purified PE-P(CL-*co*-DL)-PE (P-3). (b) SEC traces of P-2, P-4, and P-5. 12](#_Toc190172921)

[Figure S8. ^13^C NMR (400 MHz, CDCl_3_) spectrum of P-3. 13](#_Toc190172922)

[Figure S9. ^31^P{^1^H} NMR (CDCl_3_) end-group analysis of P-3 (see experimental details above). No signal for PDL hydroxyl end-groups (147.1 ppm) observed for either. 13](#_Toc190172923)

[Figure S10. ^1^H DOSY NMR (500 MHz, CDCl_3_) spectrum of P-3. 14](#_Toc190172924)

[Figure S11. DSC traces (second heating curve) for polymers P-2 to P-5. 14](#_Toc190172925)

[Figure S12. DMTA of P-2 (heated from -80 to 150 °C, 1 Hz frequency, 1% amplitude strain). 15](#_Toc190172926)

[Figure S13. DMTA of P-4 (heated from -80 to 150 °C, 1 Hz frequency, 1% amplitude strain). 15](#_Toc190172927)

[Figure S14. DMTA of P-5 (heated from -80 to 150 °C, 1 Hz frequency, 1% amplitude strain). 16](#_Toc190172928)

[Figure S15. TGA profile for P-2. 17](#_Toc190172929)

[Figure S16. TGA profile for P-3. 17](#_Toc190172930)

[Figure S17. TGA profile for P-4. 18](#_Toc190172931)

[Figure S18. TGA profile for P-5. 18](#_Toc190172932)

[Table S3. Summary of SAXS data for P-2 to P-5 19](#_Toc190172933)

[Figure S19. Parameters measured during cyclic tensile test. Calculation of elastic recovery (ER) and resilience where E_L_ is energy loss, E_R_ energy recovery, ε_max_ (maximum strain), ε_min_ (minimum strain), residual strain (ε_R_). 19](#_Toc190172934)

[Figure S20. Cyclic tensile testing of (a) P-2, (b) P-4, and (c) P-5 to 200% strain. 10 cycles shown after pre-processing after stretching to 100% strain (offset by 50% for clarity). 20](#_Toc190172935)

[Figure S21. Cyclic tensile testing of (a) P-2, (b) P-3, (c) P-5, and (d) P-5 to 200% strain. 10 cycles shown after pre-processing after stretching to 100% strain. 21](#_Toc190172936)

[Table S4. Summary of elastic recovery (ER), resilience, and residual strain (ε_R_) for all samples 21](#_Toc190172937)

[Figure S22. Samples P-3 and P-4 before and after tensile testing showing transition from transparent to opaque. 22](#_Toc190172938)

[Figure S23. Evolution of 2D-WAXS patterns of P-1 recorded at increasing % strain (ε). 22](#_Toc190172939)

[Figure S24. Evolution of 2D-WAXS patterns of P-2 recorded at increasing % strain (ε). 23](#_Toc190172940)

[Figure S25. Evolution of 2D-WAXS patterns of P-4 recorded at increasing % strain (ε) and after recovery to 0% strain. 23](#_Toc190172941)

[Figure S26. 1D-WAXS intensity profiles at increasing % strain (ε) and after recovery to 0% strain for a) P-1 and b) P-2. 24](#_Toc190172942)

[Figure S27. Deconvoluted 1D-WAXS intensity profile of P-4 after recovery to 0% strain. 24](#_Toc190172943)

[Figure S28. 2D-WAXS pattern of P-3 recorded at 1500% strain after extension at (a) 10 mm min^-1^ strain rate and (b) 50 mm min^-1^ strain rate. 1D-WAXS intensity profiles at increasing % strain (ε) recorded at (c) 10 mm min^-1^ strain rate and (d) 50 mm min^-1^ strain rate. 25](#_Toc190172944)

[Figure S29. (a) 2D-WAXS profiles taken during the stress relaxation of P-3 extended to 1500% for 3 h. b) 1D-WAXS intensity profiles at increasing P-3 at each time point and after recovery to zero stress. 26](#_Toc190172945)

[Figure S30. (a) Evolution of 2D-WAXS patterns of P-5 recorded at increasing % strain (ε) and at break (ε_b_). (b) 1D-WAXS intensity profiles at increasing % strain (ε) and at break (ε_b_). 26](#_Toc190172946)

[Figure S31. DMA relative humidity sweeps for (a) P-2, (b) P-3, (c) P-4, and (d) P-5. 27](#_Toc190172947)

[Figure S32. Rheological temperature sweep of P-3 where the G’ to G’’ crossover occurred at 149 °C. 28](#_Toc190172948)

[Figure S33. Schematic of mechanical recycling by compression remolding. 28](#_Toc190172949)

[Table S5. Summary of TPE properties. 29](#_Toc190172950)

[Figure S34. Tensile stress-strain curves (representative of five repeats) comparing P-3 and P-4 to commercial SEBS and PU. 30](#_Toc190172951)

[Figure S35. Cyclic testing of SEBS to 200% strain (offset by 50% for clarity). 30](#_Toc190172952)

[Figure S36. Cyclic testing of SEBS to 200% strain. 31](#_Toc190172953)

[Figure S37. Cyclic testing of PU to 200% strain (offset by 50% for clarity). 31](#_Toc190172954)

[Figure S38. Cyclic testing of PU to 200% strain. 32](#_Toc190172955)

[Figure S39. Comparison of elastic recovery (ER), resilience, and residual strain (ε_R_) for P-3, P-4 , SEBS an PU. 32](#_Toc190172956)

[Figure S40. Creep -recovery experiments commercial SEBS and PU. 33](#_Toc190172957)

[Figure S41. DMA relative humidity sweeps for (a) commercial SEBS and (b) commercial PU. 33](#_Toc190172958)

[Figure S42. a) Tensile strength, b) Strain at break, c) Tensile toughness, and d) Young’s Modulus for virgin commercial SEBS and after five mechanical reprocessing cycles. Mean values ±std. dev. from measurements conducted independently on five specimens. 34](#_Toc190172959)

[Figure S43. a) Tensile strength, b) Strain at break, c) Tensile toughness, and d) Young’s Modulus for virgin commercial PUand after five mechanical reprocessing cycles. Mean values ±std. dev. from measurements conducted independently on five specimens. 34](#_Toc190172960)

[Figure S44. DSC trace for P-3 and P-3 (dotted) measured immediately after tensile testing. 35](#_Toc190172961)

[References 36](#_Toc190172962)

Experimental Details

NMR Spectroscopy. ^1^H and ^31^P{^1^H} NMR spectra were obtained using a Bruker AVIII HD 400 NMR spectrometer. ^13^C {^1^H} NMR spectra were obtained using a Bruker NEO 600 NMR spectrometer. ^1^H DOSY spectra were obtained using a Bruker AVIIIHD 500 NMR spectrometer.

Size Exclusion Chromatography **(SEC).** Polymers (2-5 mg) dissolved in THF. Samples were passed through 0.2 µm PTFE filters prior to analysis. Analysis was carried out on a Shimadzu LC-20AD instrument, equipped with a Refractive Index (RI) detector and two PSS SDV 5 µm linear M columns. HPLC grade THF was used as the eluent at 1.0 mL/min at 30 °C. Monodisperse polystyrene standards were used for calibration.

Phosphorus End-Group Tests. Following literature procedure.^[1]^ Polymer samples (40 mg) were dissolved in CDCl_3_ (0.5 mL) and a solution (40 µL) containing Cr(acac)_3_ (5.5 mg) and internal standard, bisphenol A (400 mg) in pyridine (10 mL) followed by 40 μL of 2-chloro-4,4,5,5-tetramethyl dioxaphospholane.

Differential Scanning Calorimetry **(DSC).** Recorded for purified polymer samples of triblock copolymers were measured using a DSC25 (TA Instruments). A sealed, empty crucible was used as a reference, and the DSC was calibrated using sapphire and indium. Samples were heated from -80 °C to 150 °C, at a rate of 10 °C min^-1^ under N_2_ flow (80 mL min^-1^) followed by a 5-minute isotherm at 150 °C to erase thermal history. Samples were subsequently cooled to -80 °C, at a rate of 10 °C min^-1^, and kept at -80 °C for a further 5 minutes, followed by a heating-cooling procedure from -80 °C to 150 °C, at a rate of 10 °C min^-1^. Each sample was analysed over two heating-cooling cycles. Glass transition temperatures (*T*_g_) are reported as the midpoint of the transition taken from the second heating cycle.

Thermogravimetric Analysis (TGA). Measured using a TGA5500 system (TA Instruments). Samples were heated from 30 °C to 600 °C, at a rate of 5 °C min^-1^, under N2 flow (100 cm^3^ min^-1^).

Film Preparation. For mechanical testing, transparent films were prepared by solvent casting into Teflon moulds from DCM. The solvent allowed to dry at room temperature for being dried in a vacuum oven at 60 °C for at least 48 hours or until no solvent was observed by NMR or TGA analysis.

Tensile Testing. Tests were carried out using a universal testing machine (Instron 6800). Dumbbell-shaped specimens were cut using a Zwick ZCP020 cutting press equipped with a cutting device for ISO 527-2 type 5B (length= 35 mm, gauge length = 10 mm, width = 2 mm). Uniaxial extension experiments (10 mm min^-1^ cross-head speed) were run according to ISO 527. 5 specimens were tested for each material.

Dynamic Mechanical Thermal Analysis (DMTA). DMTA was carried out using a DMA850 (TA Instruments), using an ACS III cooling system. Specimens of uniform width (5.3 mm) were cut using two parallel blades. Samples were heated from -80 °C to 250 °C (or until the material deformed beyond the limits of the geometry employed) at a rate of 3 °C min^-1^, with a frequency of 1 Hz, 0.1 N pre-load force and 0.1 % strain amplitude. Glass transition temperatures (*T*_g_) are reported as the peak maxima in tan (δ). Relative humidity analysis (RH DMA) was carried out with a DMA-RH accessory. RH DMA experiments were carried out at 30 °C, with a frequency of 1 Hz, 0.1 N pre-load force and 0.1 % strain amplitude. Relative humidity was increased from by 20% every 50 minutes.

Rheology. Shear storage (G’) and loss (G’’) moduli were measured on a TA ARES-G2 instrument using polymer samples between 25 mm or 8 mm stainless steel plates. Measurements were conducted in the linear viscoelastic region as determined by amplitude sweeps. Temperature sweep experiments were conducted from 30 °C to 200 °C at a heating rate of 2 °C min^-1^, 1 Hz frequency. Creep recovery experiments were conducted on an ARES-G2 (TA Instruments) using polymer samples between 8 mm stainless steel plates. Stress control conditioning experiments were conducted at 30 °C in the linear viscoelastic region as determined by amplitude sweeps. 5 kPa of stress was applied to the samples for 100 s followed by a period of 0 kPa for 100 s and the strain (creep) exhibited monitored, this was repeated a further 3 times.

Small-angle X-ray Scattering (SAXS). Polymer films (prepared as for mechanical testing as described above) in a solid sample grid were submitted to beamline station I22, located at Diamond Light Source, Harwell, United Kingdom. A monochromatic X-ray radiation (λ = 0.1 nm) and 2D SAXS detector (Pilatus P3-2M, DECTRIS Ltd.) were used for the experiments. 2D scattering patterns were reduced to 1D using Dawn software developed at the Diamond Light Source.^[2]^

Wide-angle X-ray Scattering (WAXS). Performed at beamline station I22, located at Diamond Light Source, Harwell, United Kingdom. A monochromatic X-ray radiation (λ = 0.1 nm) and 2D WAXS detector (Pilatus3-2M-DLS-L, DECTRIS Ltd.) were used for the experiments. Specimens were prepared as for tensile testing described above. For the tensile-WAXS measurements, the uniaxial extension experiments were carried out on a Shimadzu EZ-LX Universal testing instrument at an extension rate of 10 mm min^-1^ to 50 mm min^-1^. Specimens were stretched to the selected strains at 23 °C before the WAXS measurement. The 2D detector images were azimuthally integrated between 170° to 190° to produce 1D intensity profiles using Dawn software developed at the Diamond Light Source.^[2]^

# Reagents and Methods

The macrocyclic ligand, H_2_L, was synthesized following a previously reported procedure.^[3]^ Magnesium bis(1,1,1,3,3,3-hexamethyldisilazan-2-ide) (97%) and bis(pentafluorophenyl)zinc (97%) were purchased from Sigma-Aldrich. Bis(pentafluorophenyl)zinc was used as received and magnesium bis(1,1,1,3,3,3-hexamethyldisilazan-2-ide) was recrystallized from hexane. Solvents used for synthesis and polymerization were collected from a solvent purification system (SPS), degassed with three freeze-pump-thaw cycles, and stored over 4 Å molecular sieves, under an inert atmosphere. ε-Decalactone (ε-DL) (Sigma-Aldrich, 98% purity) was dried over CaH_2_ followed by fractional distillation at 120 °C under reduced pressure and stored under a nitrogen atmosphere. ε-Caprolactone (ε-CL) (Sigma-Aldrich, 98% purity) was dried over CaH_2_ followed by fractional distillation at 120 °C under reduced pressure and stored under a nitrogen atmosphere. Cyclohexene 1,2-epoxide (98 %) (CHO) was purchased from Alfa Aesar, dried by stirring over CaH_2_, followed by fractional distillation at 60 °C and stored under an inert atmosphere. Phthalic anhydride (PA) (Sigma Aldrich, anhydrous, 98% purity) was stirred in dry toluene (purified by SPS) for 16 hr. After cannula filtration and removal of toluene *in vacuo*, the white powder was recrystalised from anhydrous chloroform and sublimed three times under vacuum at 80 °C then stored in a glovebox. 1,4-Benzenedimethanol (BDM) was recrystallized from toluene and stored under inert atmosphere. Commercial elastomers Estane® 58887 (PU) (acquired from Goodfellow) and Septon™ 8004 (SEBS) (acquired from Kuraray) were used as received.

Synthesis of [LZnMg(C_6_F_5_)_2_] Catalyst The catalyst was synthesized following a previously reported procedure.**^[3]^** Under inert conditions, the macrocyclic ligand (H_2_L) (0.50 g, 0.90 mmol) and recrystallised Mg{N[Si(CH_3_)_3_]_2_}_2_ (0.31 g, 0.90 mmol) were dissolved in THF (10 mL), at 25 °C, for 1 hour. Zn(C_6_F_5_)_2_ (0.36 g, 0.90 mmol) was dissolved in THF (5 mL) and added dropwise to the reaction mixture to afford an off-white solution which was stirred overnight at 25 °C. Solvent was removed *in vacuo* to yield a off-white solid. The product was isolated by washing with cold (-30 °C) THF (5 mL) and pentane (2 x 5 mL) followed by centrifugation. The off-white powder was dried in vacuo overnight at 25 °C (0.62 g, 70% yield). ^1^H NMR (400 MHz, CDCl_3_) δ 6.81 (s, 2H), 6.76 (s, 2H), 4.42 – 4.25 (m, 4H), 3.40 (d, J = 13.7 Hz, 2H), 3.26 (d, J = 13.3 Hz, 2H), 3.14 – 2.94 (m, 4H), 2.68 (s, 6H), 2.11 – 2.00 (m, 2H), 1.27 (s, 3H), 1.20 (s, 18H), 1.17 (s, 3H), 1.06 – 1.01 (m, 6H).

General Polymerization Procedure. In a glovebox, ε-DL (3.6 mL, 20.5 mmol, 1000 equiv.) followed by ε-CL (2.27 mL, 20.5 mmol, 1000 equiv.) was added to a solution of CHO (1.2 mL, 11.8 mmol, 577 equiv.), 1,4-BDM (5.0 mg, 0.0410 mmol, 2 equiv.), and [LZnMg(C_6_F_5_)_2_] (20 mg, 0.0205 mmol, 1 equiv.) in toluene (35 mL). The reaction vessel was sealed and heated to 80 °C. The progress of the reaction was monitored by ^1^H NMR spectroscopy. After 1 hour, >90% conversion of lactone was achieved. PA (1.2 g, 7.9 mmol, 384 equiv.) was added to the reaction mixture, then heated to 110 °C and stirred for 72 hours. PA conversion was determined by NMR analysis of an aliquot (>99%). The reaction mixture was quenched by exposure to air. The polymer was purified by precipitation in methanol (400 mL) three times and dried under reduced pressure to remove all solvents.

# Table S1. Composition and thermal characteristics for Poly(CL-co-DL) copolymers (mid-block compositions for block polymers).

| # | ***M*_n,SEC_ (kg mol^-1^) *^a^***  **[*Ð*]*^b^*** | ***M*_n,NMR_**  **(kg mol^-1^) *^c^*** | **wt %**  **PCL:PDL*^c^*** | **mol %**  **PCL:PDL*^c^*** | **DP_PCL_ – DP_PDL_*^c^*** | ***T*_g_ (°C)*^d^*** | ***T*_m_ (°C)*^d^*** | ***X* (%)*^e^*** |
| --- | --- | --- | --- | --- | --- | --- | --- | --- |
| **PCL** | 87 [1.4] | 78 | 100 : 0 | 100 : 0 | 685 - 0 | -62 | 56 | 40 |
| **PCD-76** | 78 [1.4] | 75 | 68 : 32 | 76 : 24 | 444 - 143 | -63 | 22 | 15 |
| **PCD-54** | 90 [1.6] | 171 | 44 : 56 | 54 : 46 | 656 - 563 | -59 | - | - |
| **PCD-36** | 121 [1.3] | 83 | 26 : 74 | 35 : 65 | 190 - 359 | -59 | - | - |
| **PDL** | 86 [1.1] | 125 | 0 : 100 | 0 : 100 | 0 - 735 | -52 | - | - |

^a^Determined from SEC analysis (THF, 1mL min^-1^), RI and UV detector, calibrated using poly(styrene) standards. ^b^*M*_w_/*M*_n_. ^c^Determined from ^1^H NMR sample of purified polymer sample. ^d^Determined by DSC, second heating curve. *Upper glass transition temperature from peak in tan(δ) by DMTA. ^e^Degree of crystallinity calculated using the reference enthalpy of fusion for fully crystalline PCL (139.5 J g^-1^).^[4]^


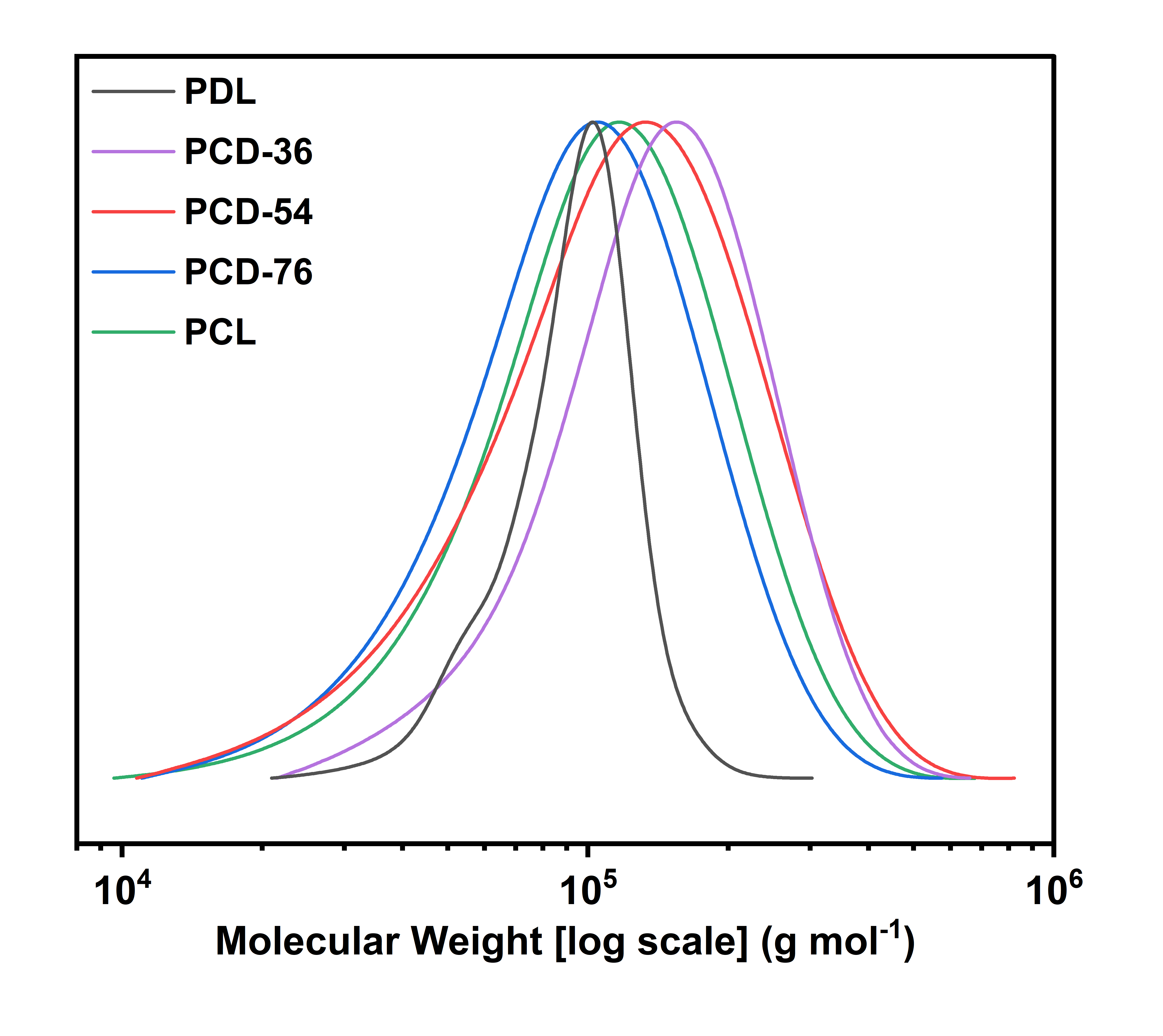


# Figure S1. SEC trace (THF eluent, 1 mL min^-1^) for poly(CL-co-DL) copolymers.


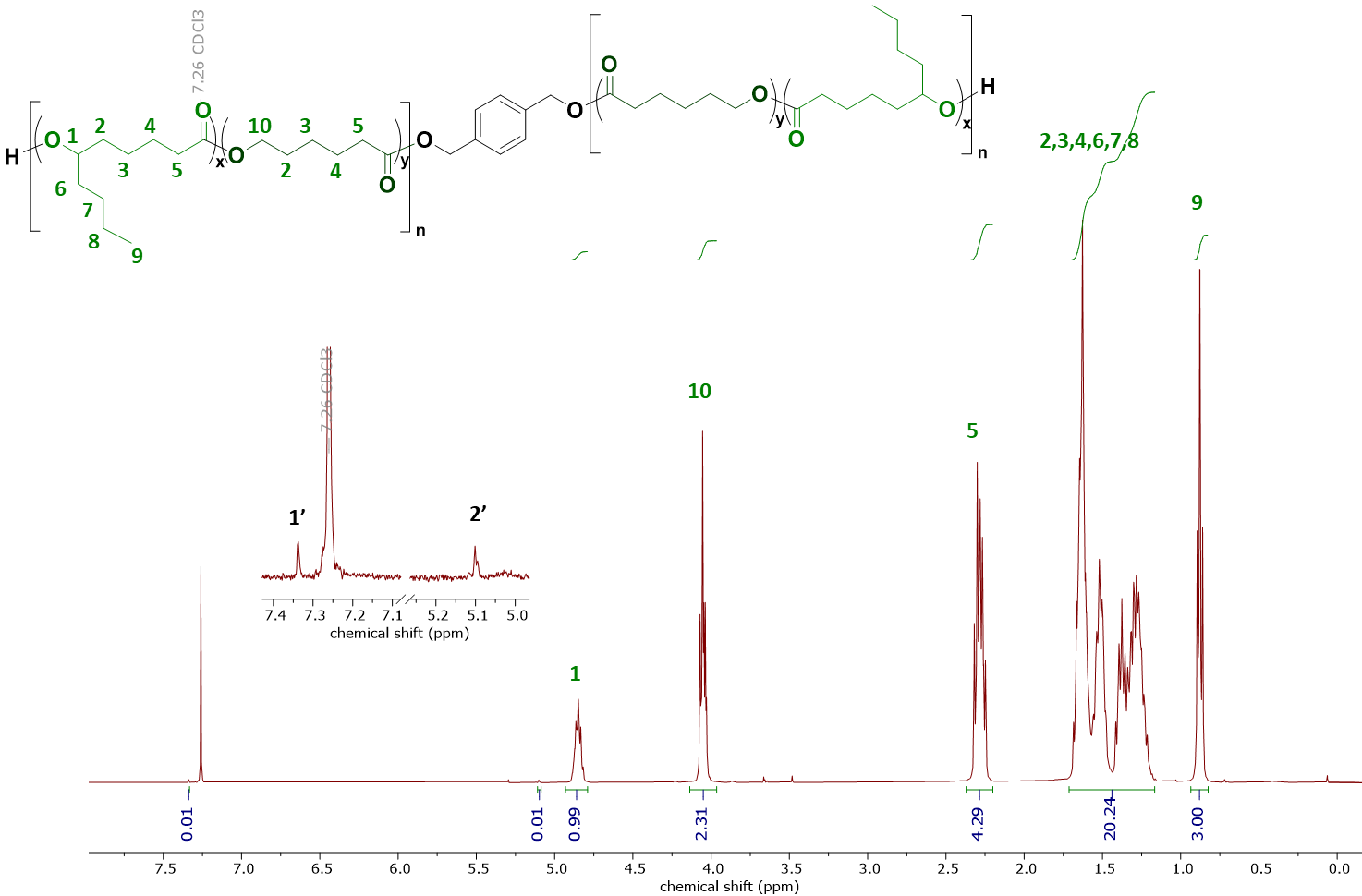


# Figure S2. ^1^H NMR (400 MHz, CDCl_3_) spectrum of PCD-76.

The Meyer-Lowry model was used to estimate the reactivity ratios at conversions <30% by fitting the compositional drift in *f*_CL_ to the total conversion *X*_n_.^[5]^

$$\chi_{n}=1-\left( \frac{f_{1}}{f_{10}} \right)^{\alpha}\left( \frac{f_{2}}{f_{20}} \right)^{\beta}\left( \frac{f_{10}-\delta}{f_{1}-\delta} \right)^{\gamma}$$

$$\alpha= \frac{r_{2}}{(1-r_{2})}; \beta= \frac{r_{1}}{(1-r_{1})}; \gamma= \frac{1-r_{1}r_{2}}{(1-r_{1})(1-r_{2})}; \delta= \frac{(1-r_{2})}{(2-r_{1}-r_{2})}$$

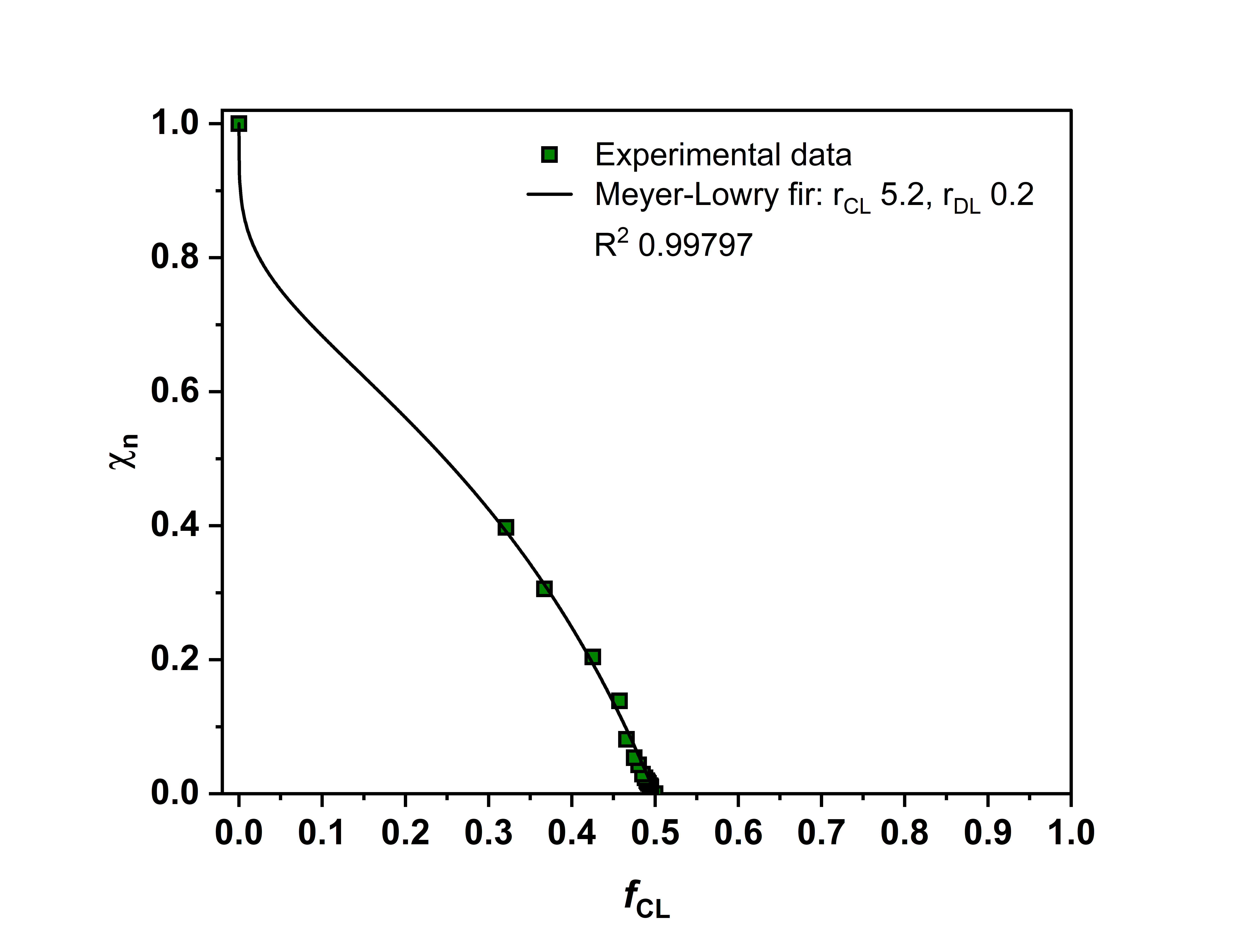


# Figure S3. Meyer-Lowry fit to ε-CL/ε-DL copolymerisation data.


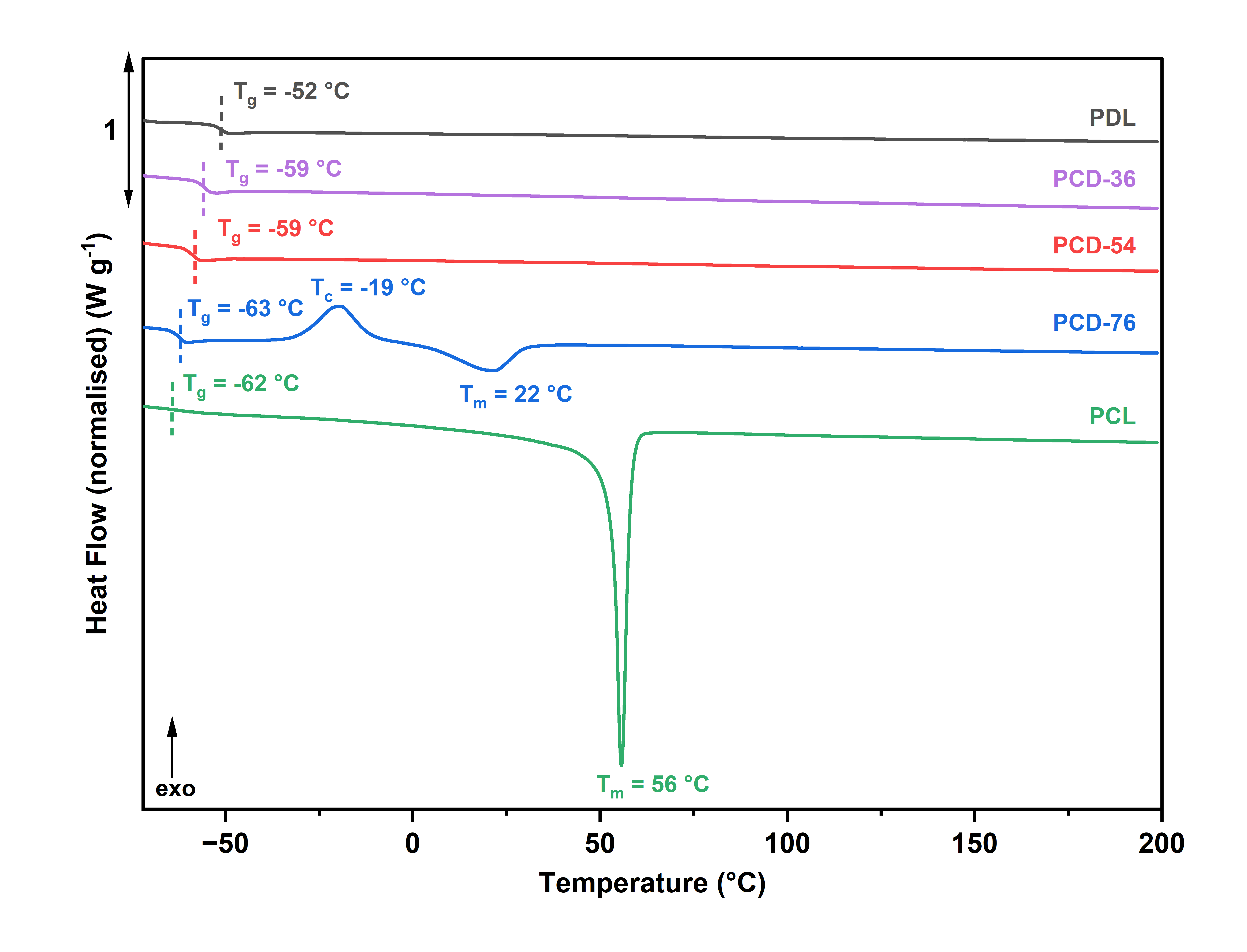


# Figure S4. Differential scanning calorimetry (DSC) traces (second heating curve) for poly(CL-co-DL) copolymers.

# Figure S5. Schematic illustrating switch catalysis strategy employed for synthesis of poly(cyclohexene-alt-phthalate)-*b*-poly(ε-caprolactone-*co*-ε-decalactone)-*b*-poly(cyclohexene-alt-phthalate) triblock copolymers

# Table S2. Summary of Polymerisation Conditions and Results

| # | **ROP** | | | **ROCOP** | | | **PCL:PDL*^d^* (wt%)** | **PCL:PDL*^d^* (mol%)** | **PA/CHO *^d^* (wt%)** |
| --- | --- | --- | --- | --- | --- | --- | --- | --- | --- |
|  | **Time (h)** | **Conv.*^a^* (%)** | ***M*_n SEC_*^b^* [Ð]*^c^***  **(kg mol^-1^)** | **Time (h)** | **Conv.*^a^* (%)** | ***M*_n SEC_*^b^* [Ð]*^c^***  **(kg mol^-1^)** |  |  |  |
| **P-2** | 3 | 88 | 126 [1.4] | 96 | 86 | 110 [1.6] | 23 : 77 | 30 : 70 | 24 |
| **P-3** | 1 | 91 | 127 [1.6] | 120 | 96 | 135 [1.4] | 48 : 52 | 58 : 42 | 26 |
| **P-4** | 1 | 94 | 102 [1.3] | 96 | 99 | 74 [1.3] | 70 : 30 | 77 : 23 | 28 |
| **P-5** | 0.5 | 99 | 251 [1.3] | 72 | 99 | 101 [1.5] | 100 : 0 | 100 : 0 | 27 |

*^a^ Determined from ^1^H NMR spectrum of reaction aliquot. ^b^Determined from SEC analysis (THF, 1mL min^-1^), RI and UV detector, calibrated using poly(styrene) standards. ^c^ M_w_/M_n_. ^d^Determined from ^1^H NMR sample of purified polymer sample.*


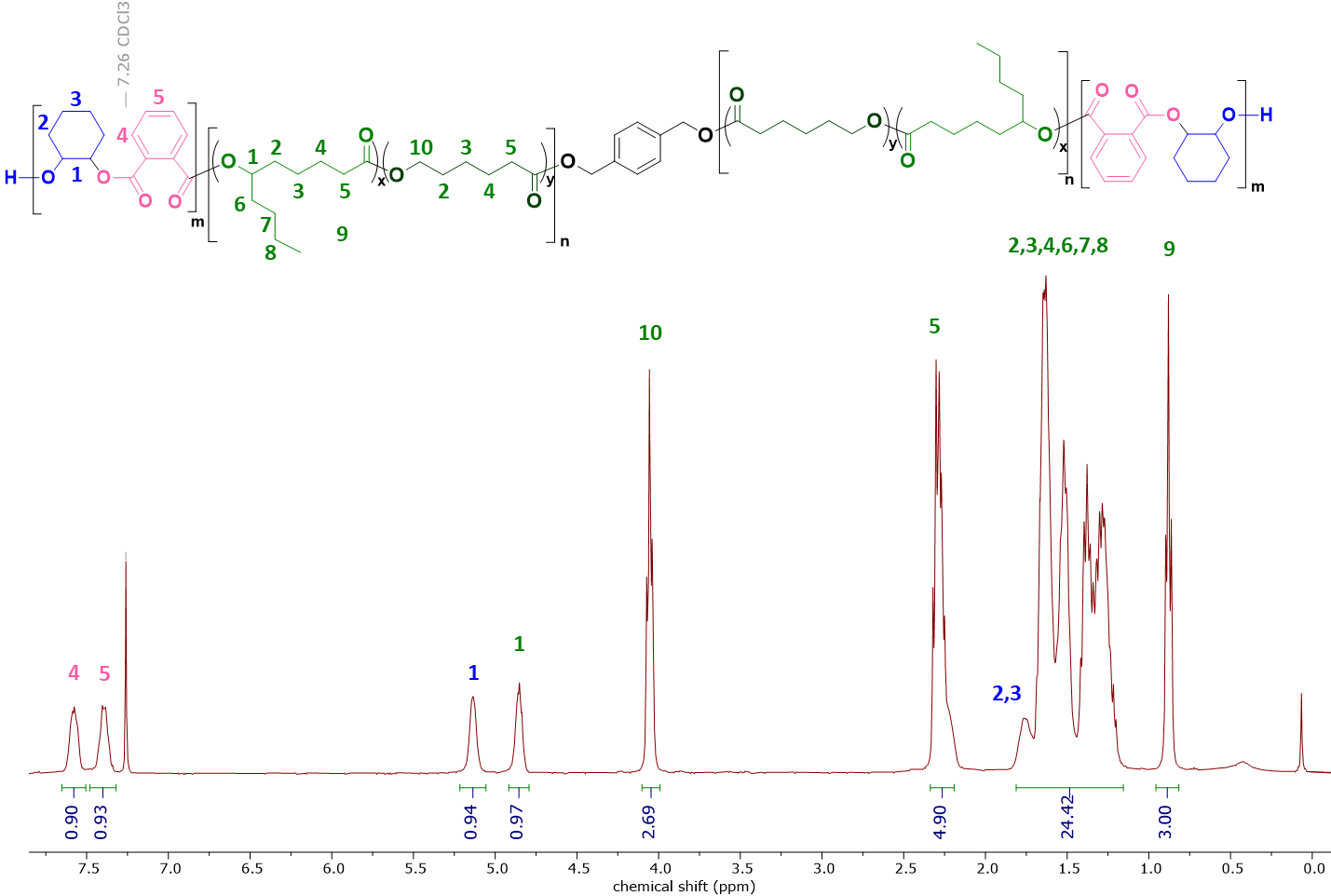


# Figure S6. ^1^H NMR (400 MHz, CDCl_3_) spectrum of P-3.


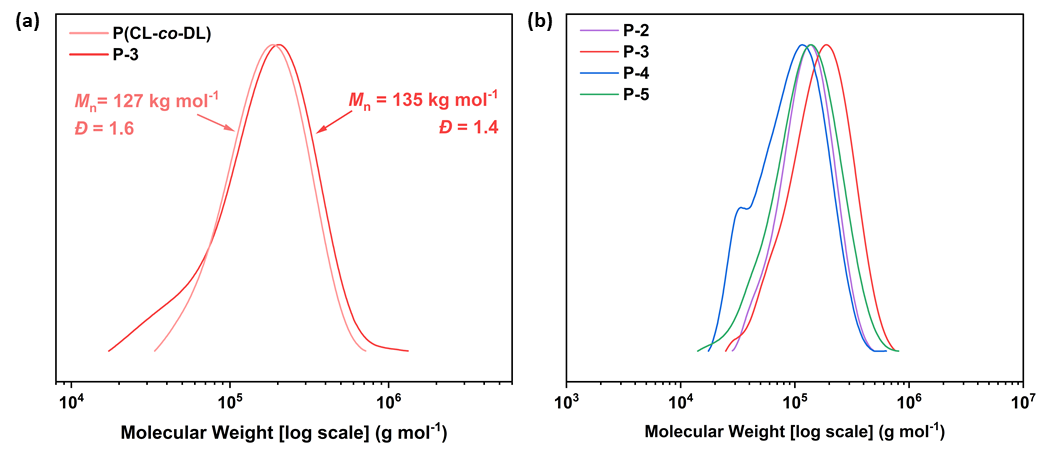


# Figure S7. SEC trace (THF eluent, 1 mL min^-1^) for (a) PDL block aliquot and purified PE-P(CL-*co*-DL)-PE (P-3). (b) SEC traces of P-2, P-4, and P-5.


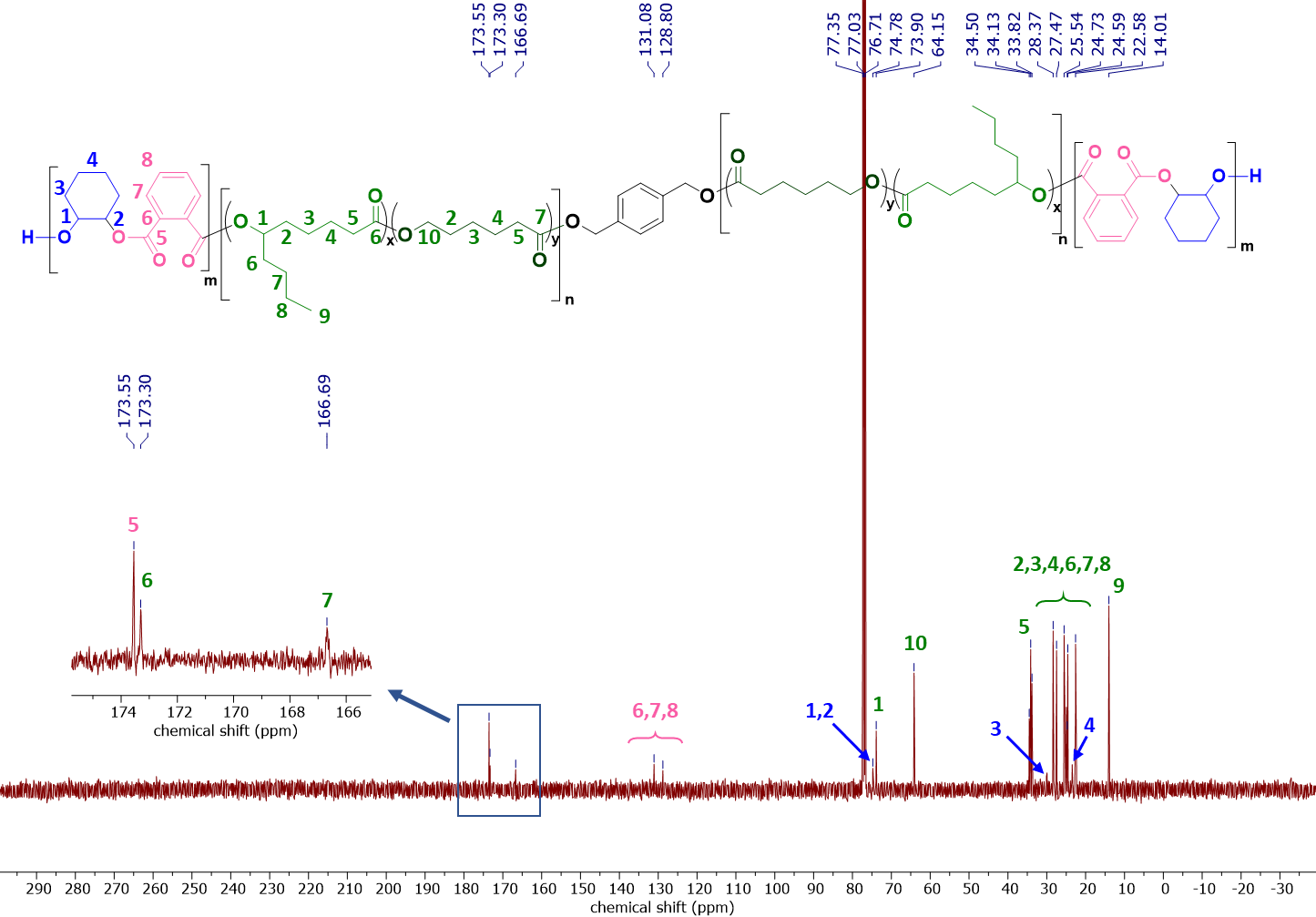


# Figure S8. ^13^C NMR (400 MHz, CDCl_3_) spectrum of P-3.


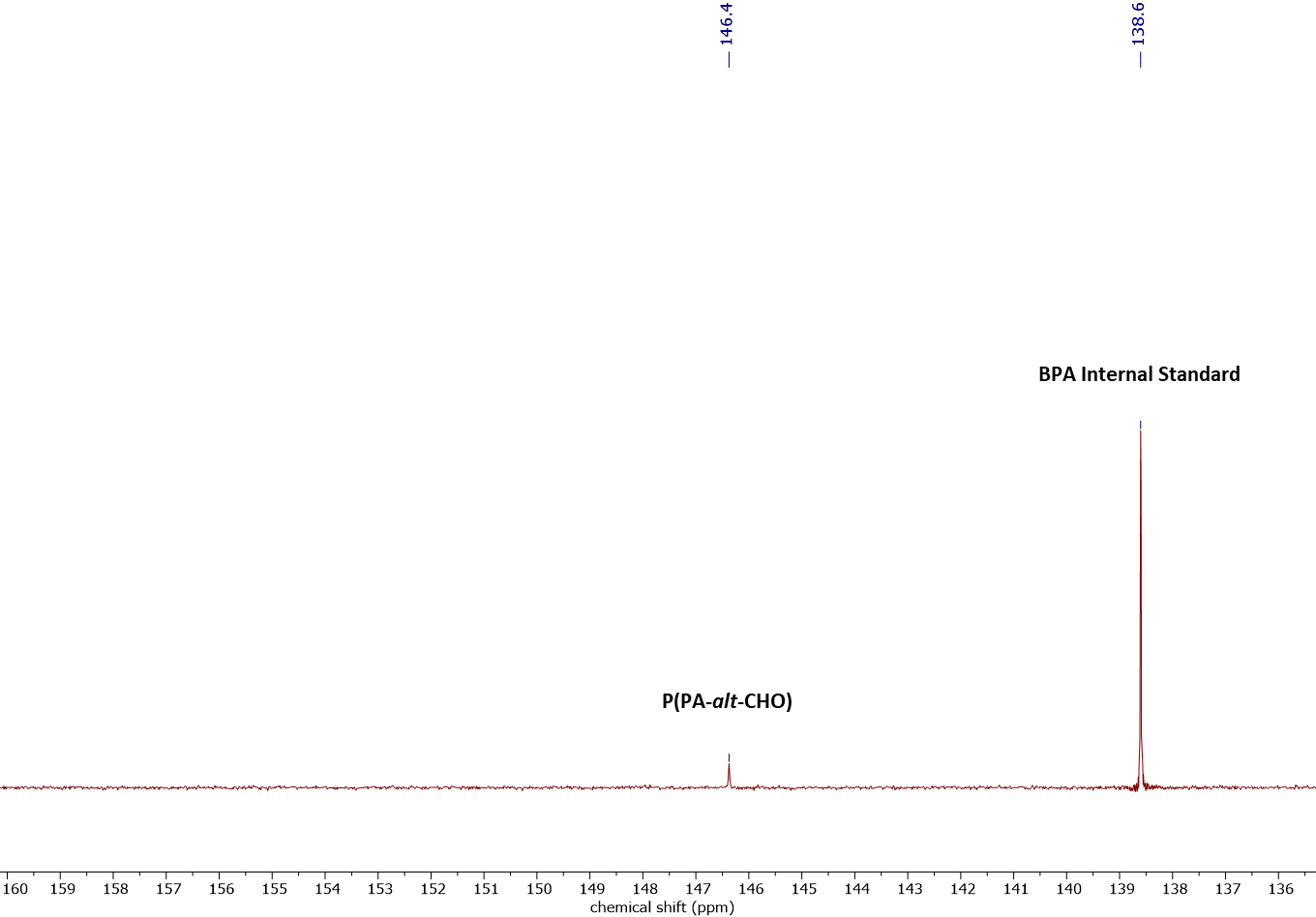


# Figure S9. ^31^P{^1^H} NMR (CDCl_3_) end-group analysis of P-3 (see experimental details above). No signal for PDL hydroxyl end-groups (147.1 ppm) observed for either.


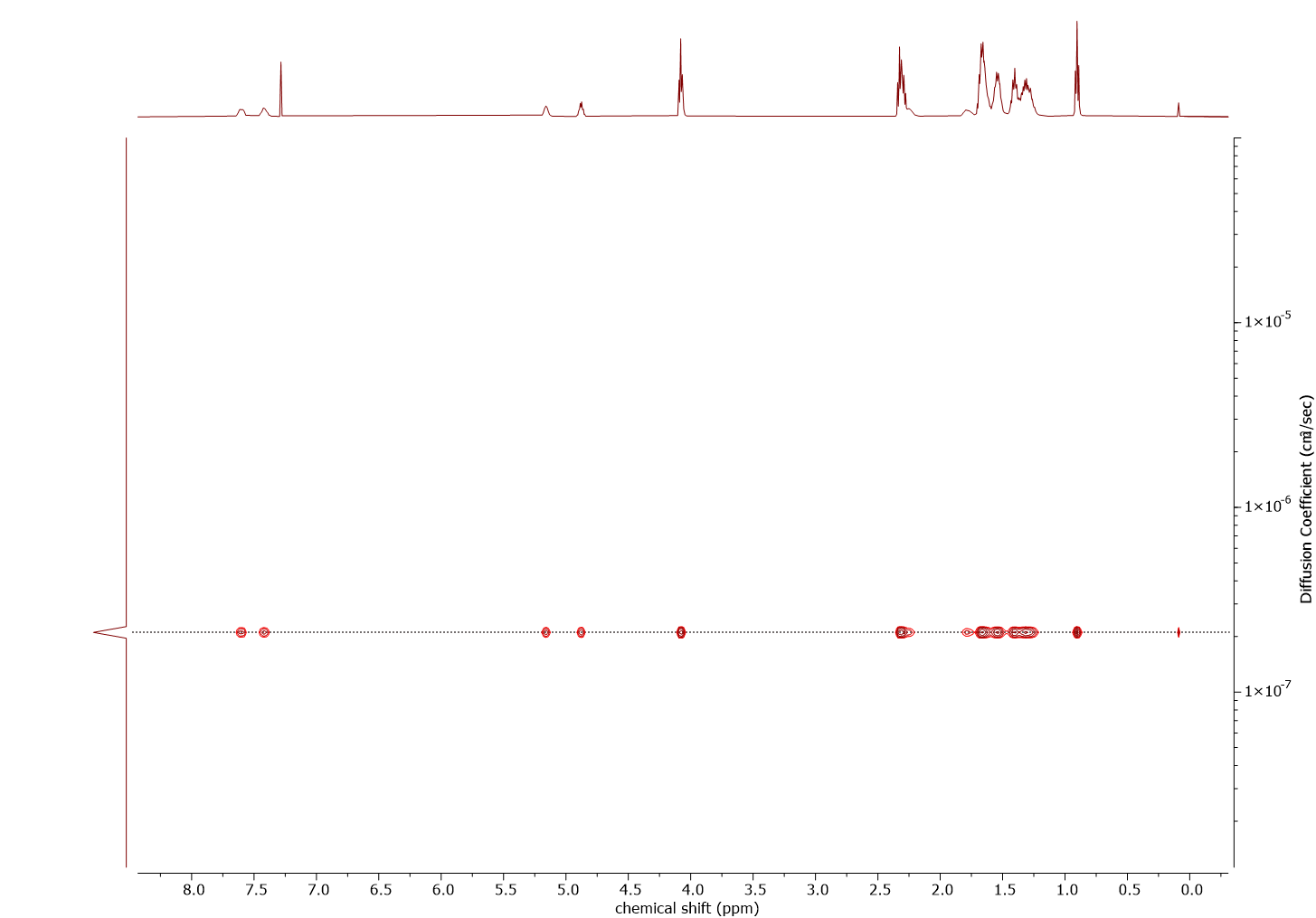


# Figure S10. ^1^H DOSY NMR (500 MHz, CDCl_3_) spectrum of P-3.


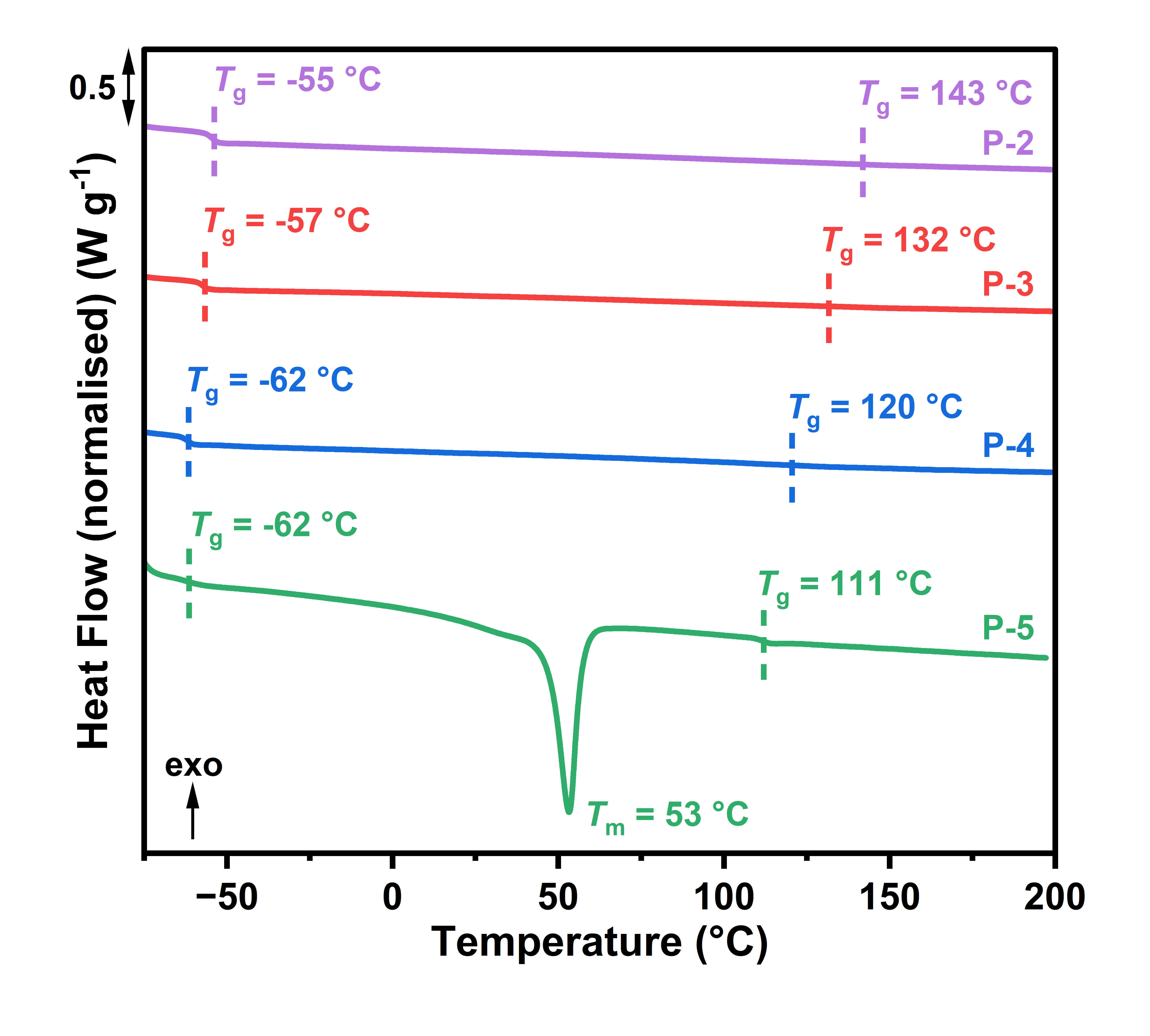


# Figure S11. DSC traces (second heating curve) for polymers P-2 to P-5.


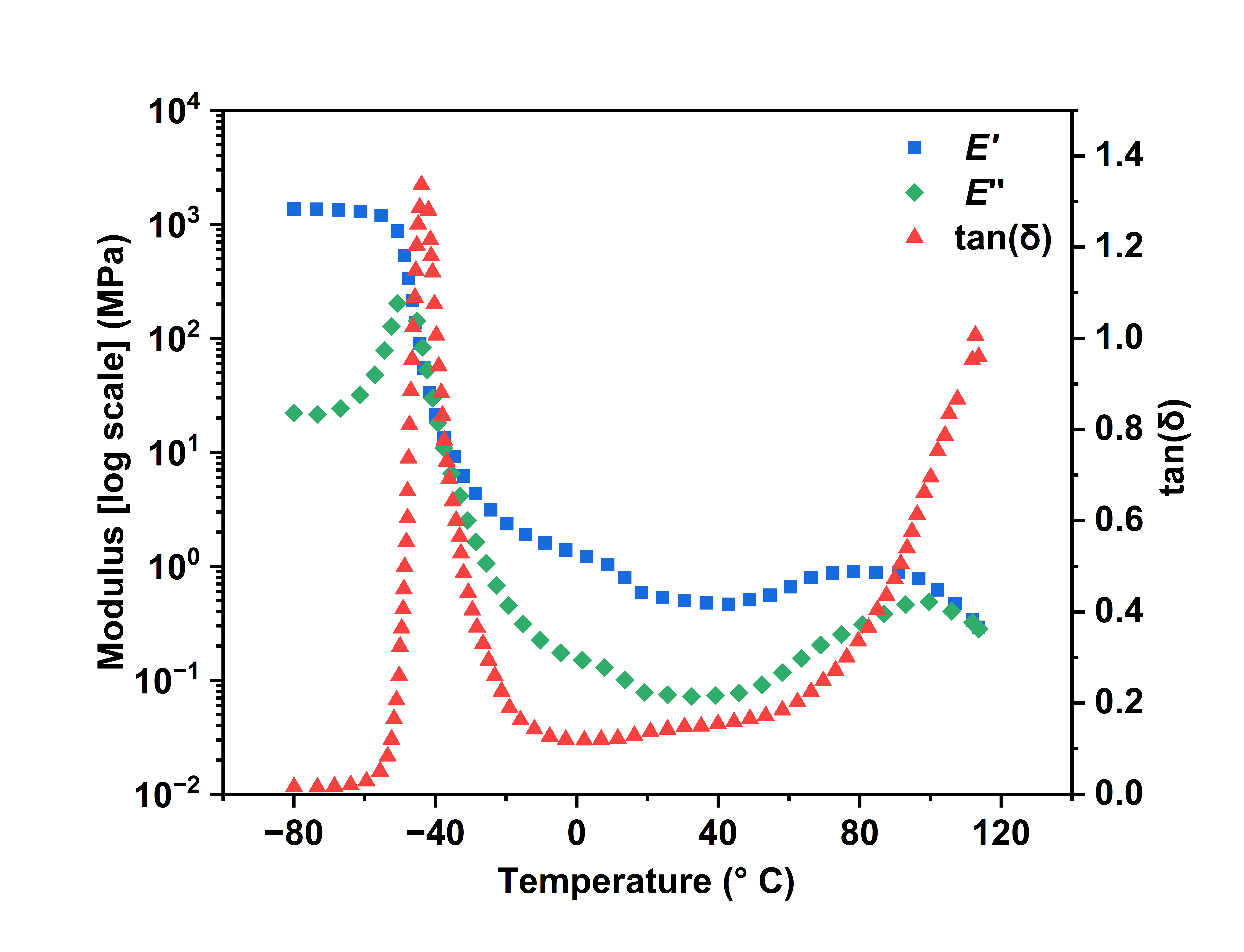


# Figure S12. DMTA of P-2 (heated from -80 to 150 °C, 1 Hz frequency, 1% amplitude strain).


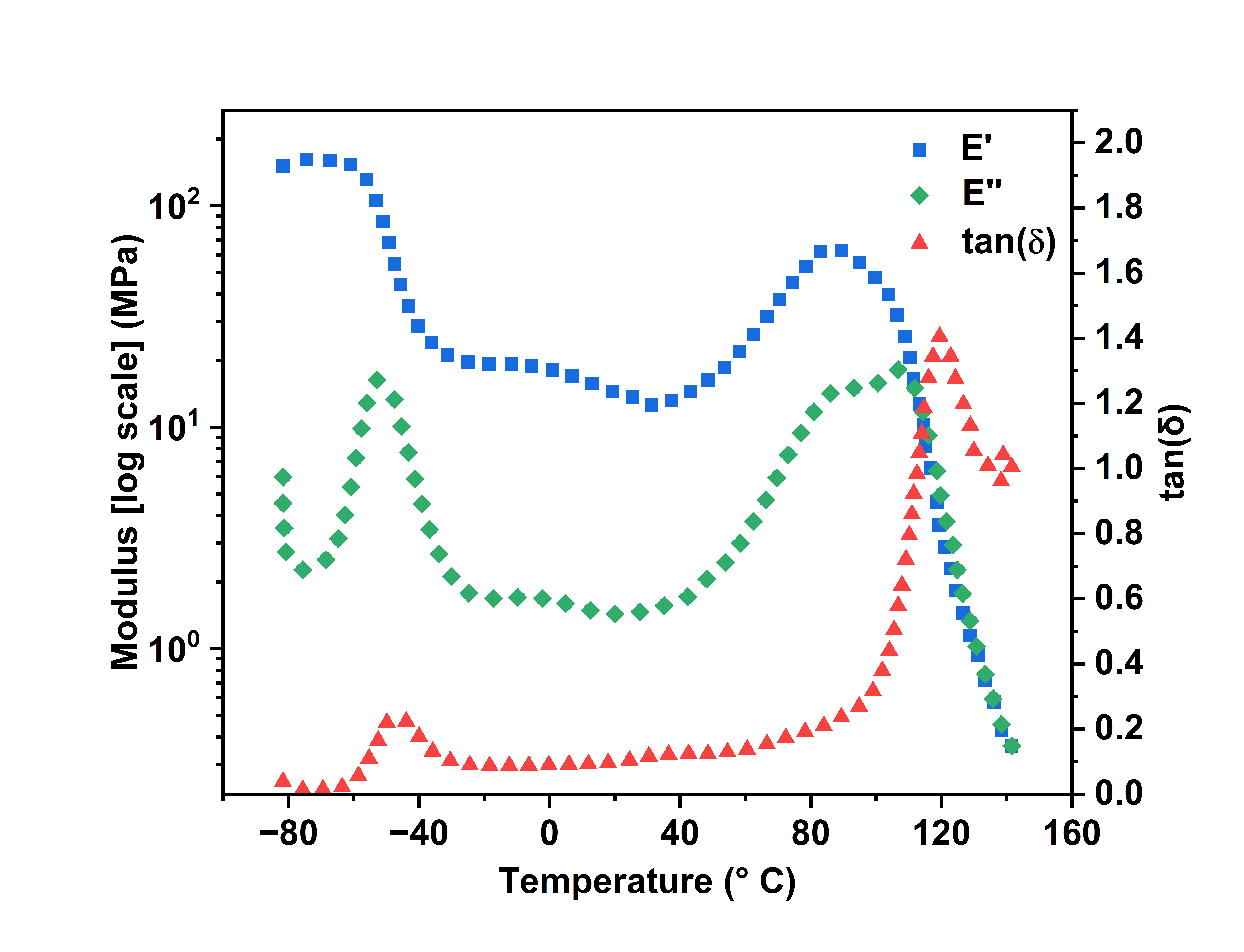


# Figure S13. DMTA of P-4 (heated from -80 to 150 °C, 1 Hz frequency, 1% amplitude strain).


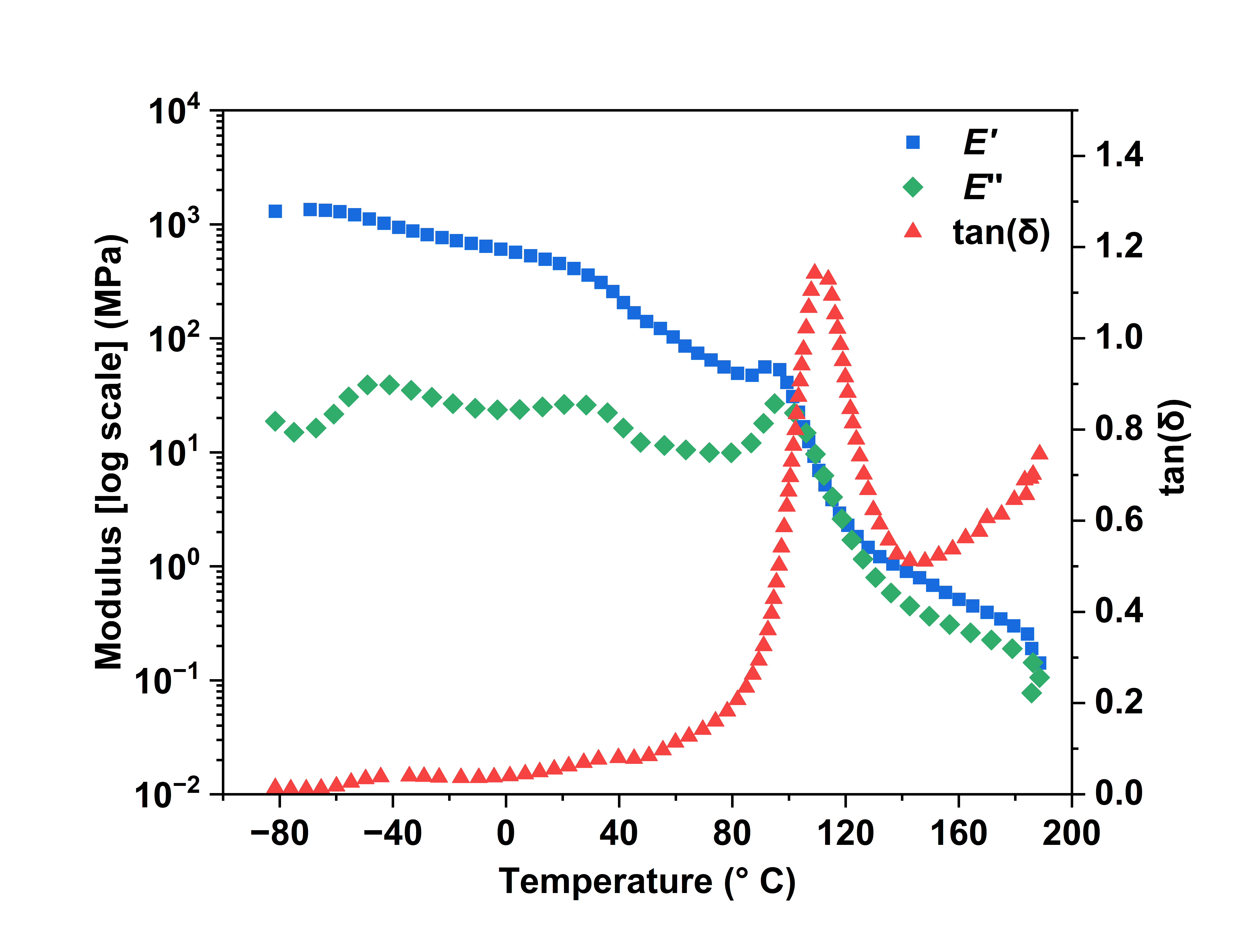


# Figure S14. DMTA of P-5 (heated from -80 to 150 °C, 1 Hz frequency, 1% amplitude strain).


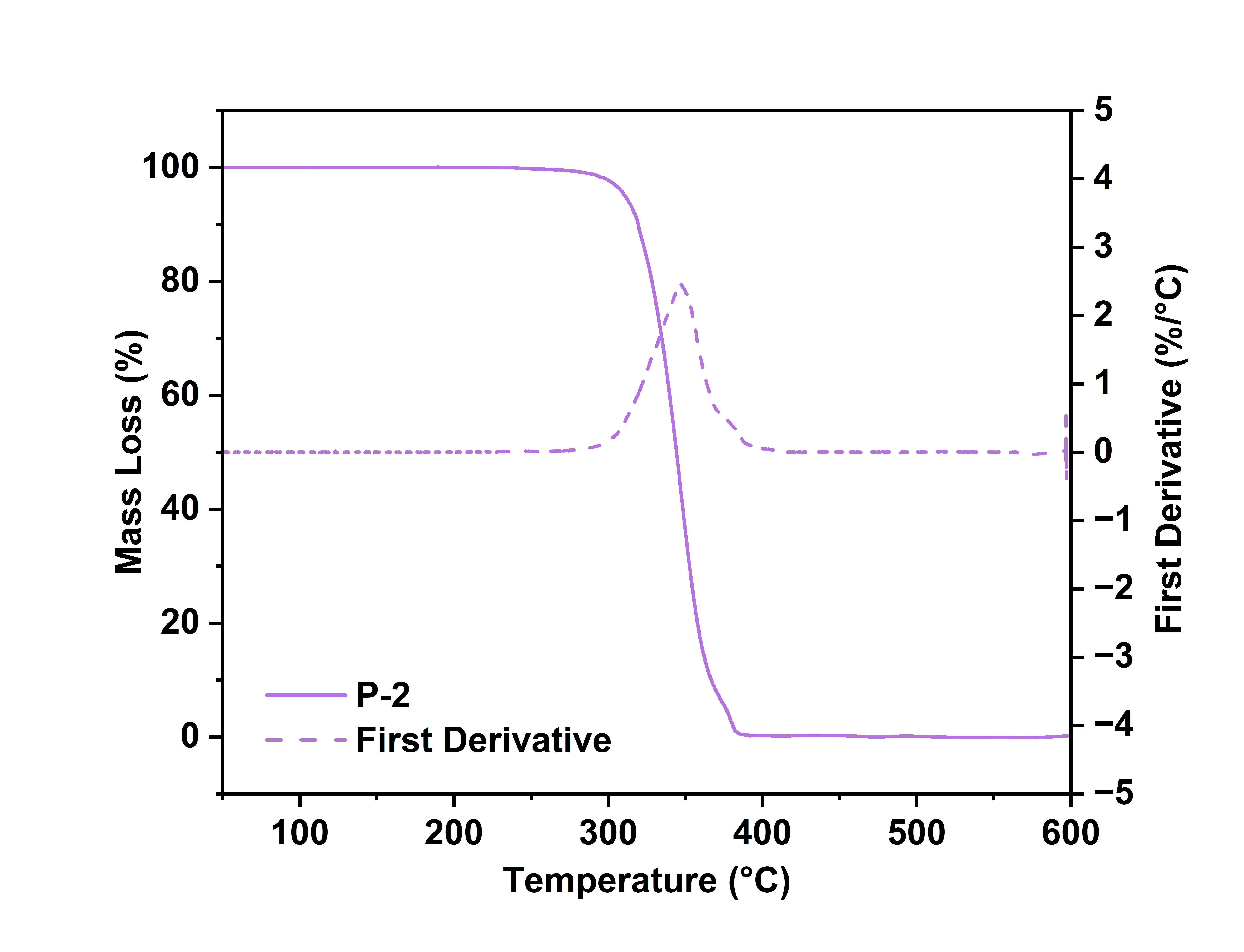


# Figure S15. TGA profile for P-2.


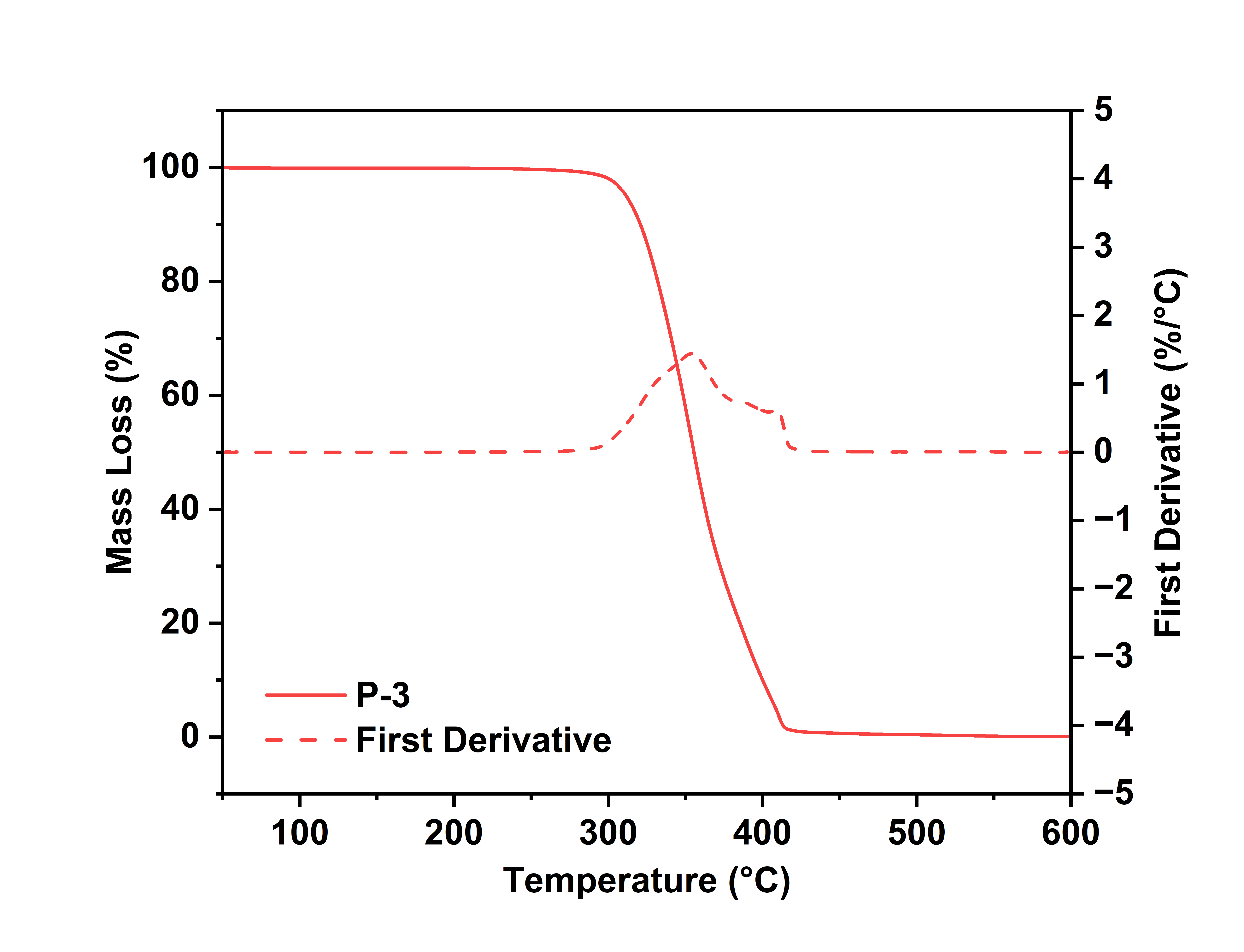


# Figure S16. TGA profile for P-3.


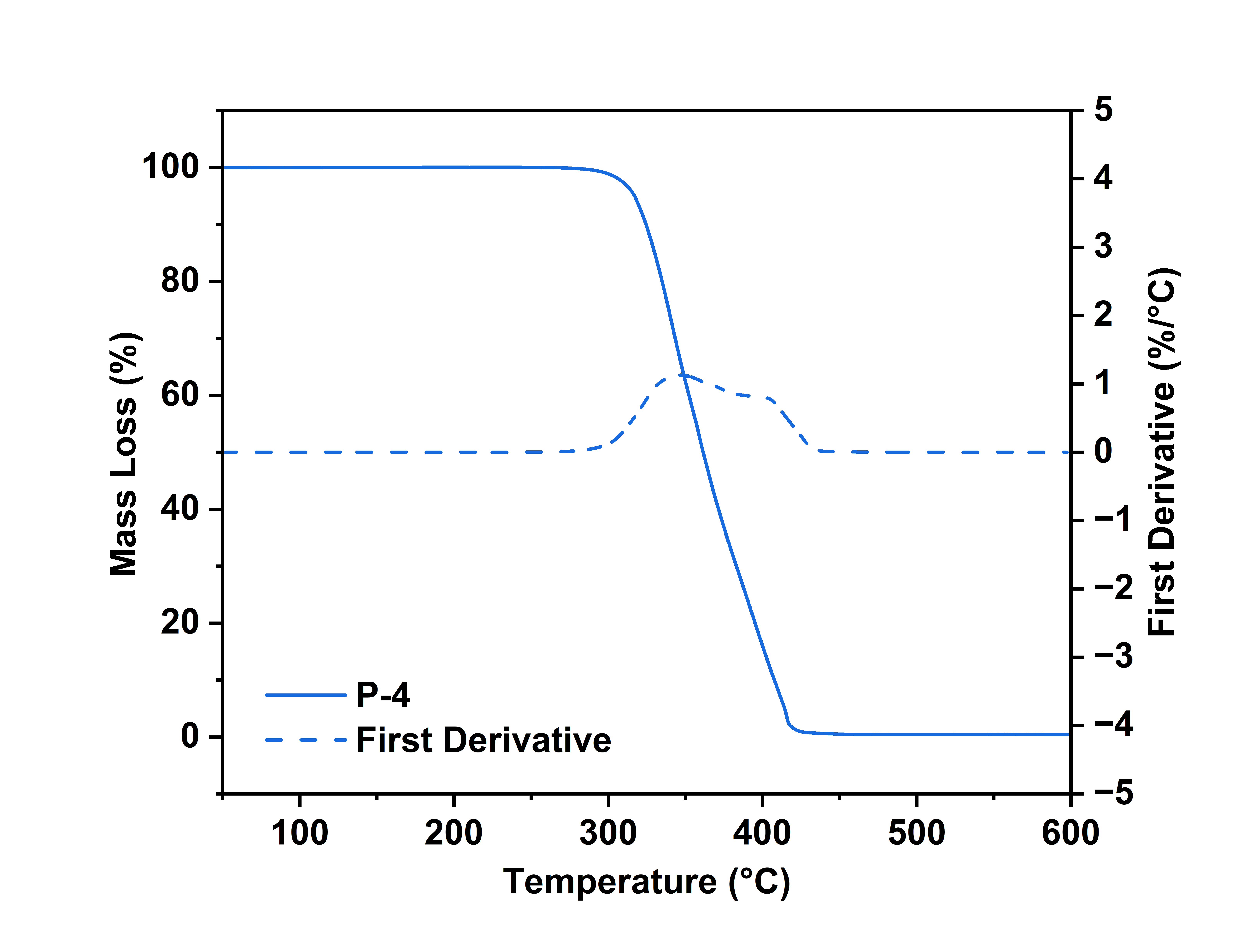


# Figure S17. TGA profile for P-4.


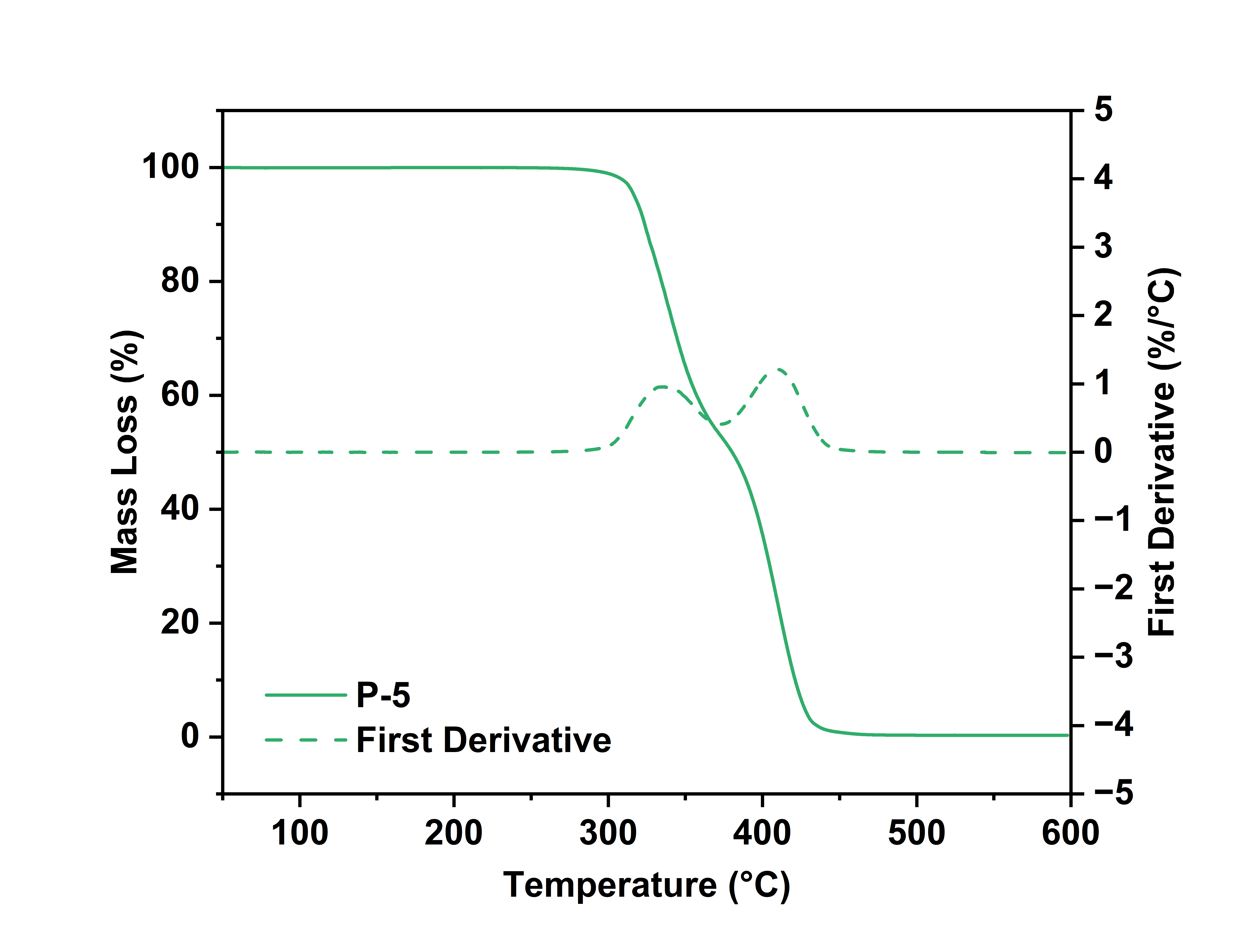


# Figure S18. TGA profile for P-5.

# Table S3. Summary of SAXS data for P-2 to P-5

| **Sample** | ***q** (Å^-1^)***^a^* | ***d* (nm)***^b^* | **Observed *q**/*q****^c^* | **Morphology***^d^* |
| --- | --- | --- | --- | --- |
| P-2 | 0.0249 | 25 | √3q* | Hex |
| P-3 | 0.0191 | 33 | √3q*, √4q*, √9q* | Hex |
| P-4 | 0.0175 | 36 | √4q* | Lam |
| P-5 | 0.0174 | 36 | √4q* | Lam |

*^a^ q** = principle scattering peak. *^b^* Domain spacing, *d*, calculated from the principle scattering peak, *^d^* = 2π/*q**. *^c^* Observed Bragg reflections. *^d^* Proposed phase morphology based on SAXS data.


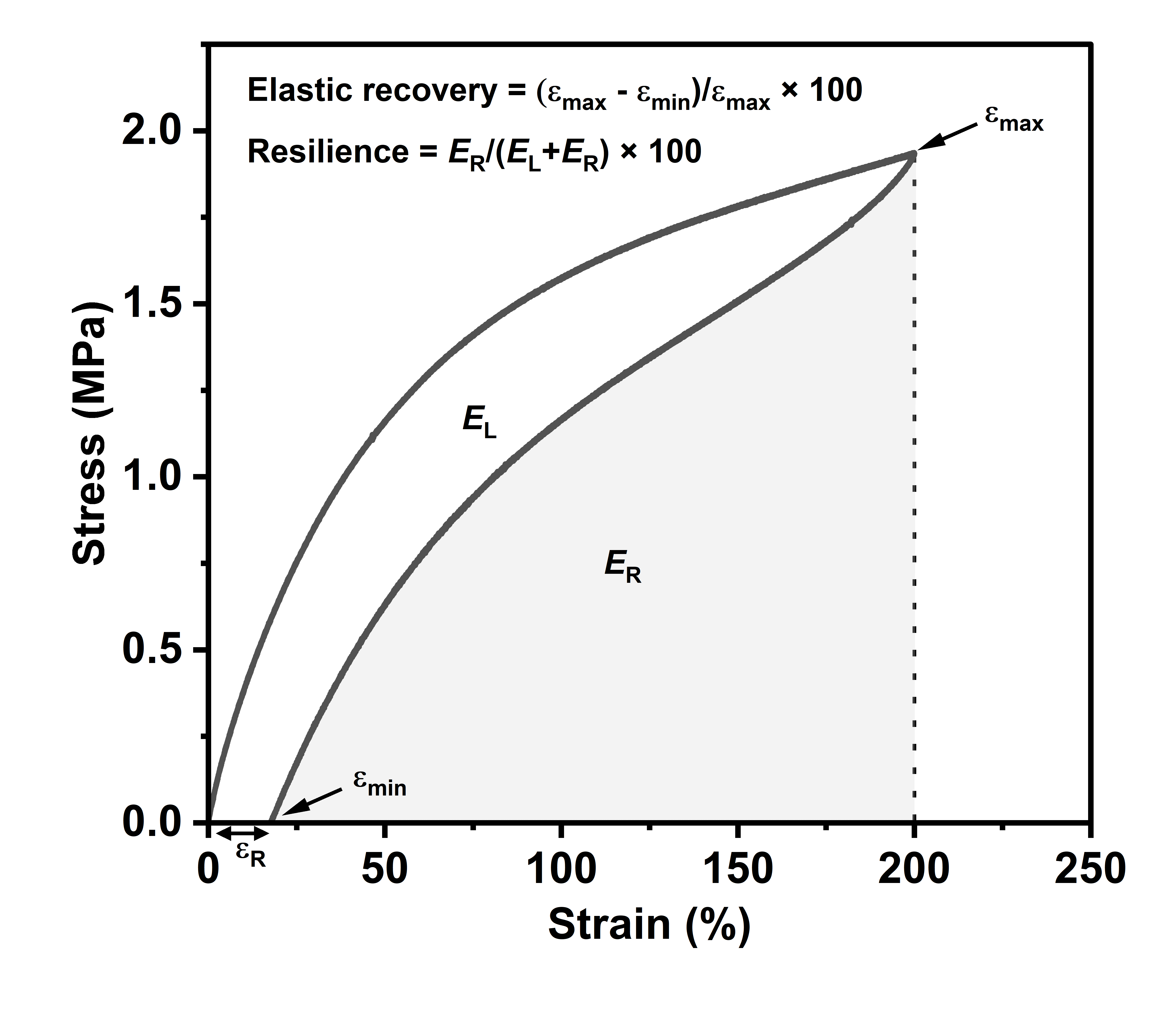


# Figure S19. Parameters measured during cyclic tensile test. Calculation of elastic recovery (ER) and resilience where E_L_ is energy loss, E_R_ energy recovery, ε_max_ (maximum strain), ε_min_ (minimum strain), residual strain (ε_R_).


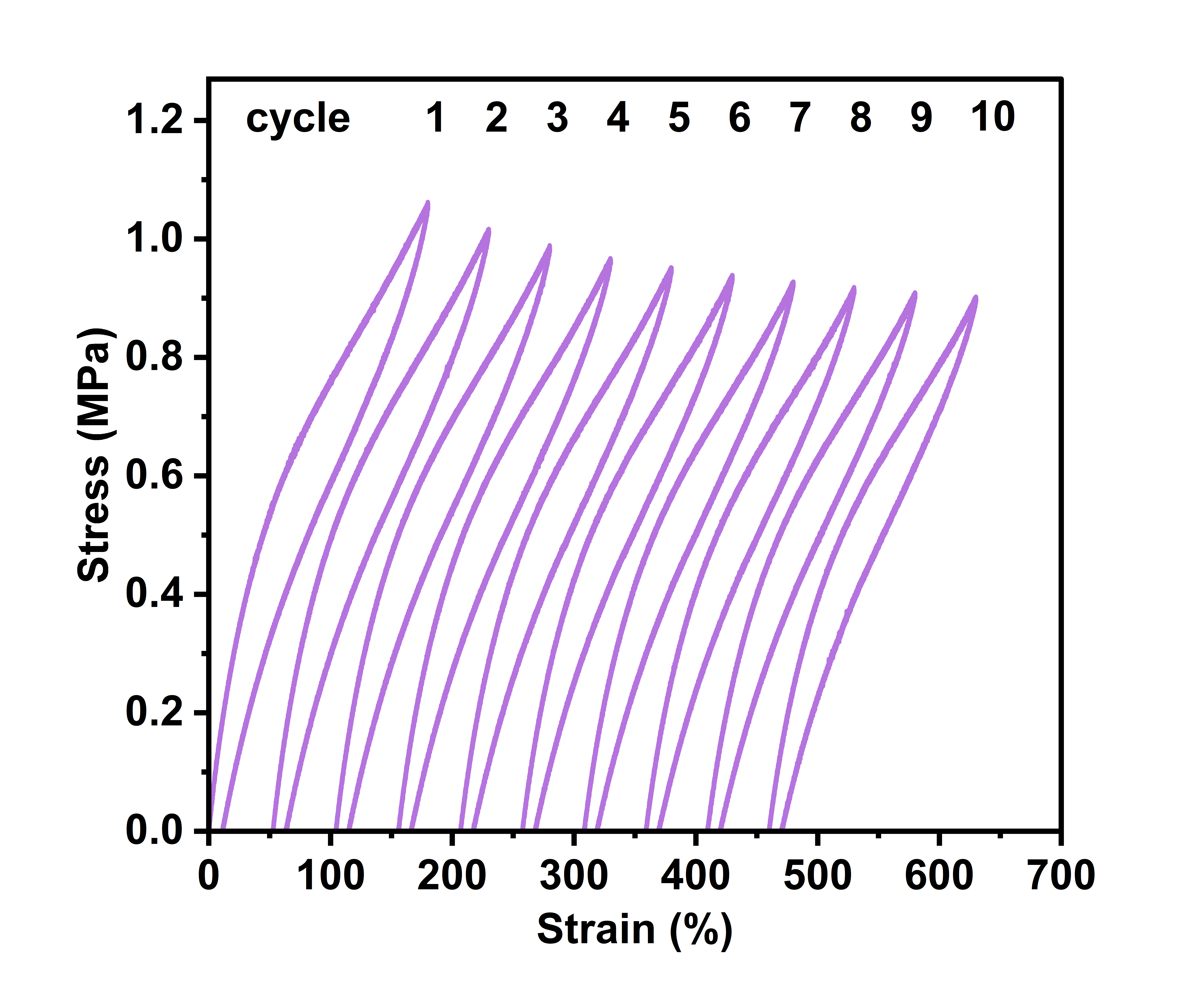

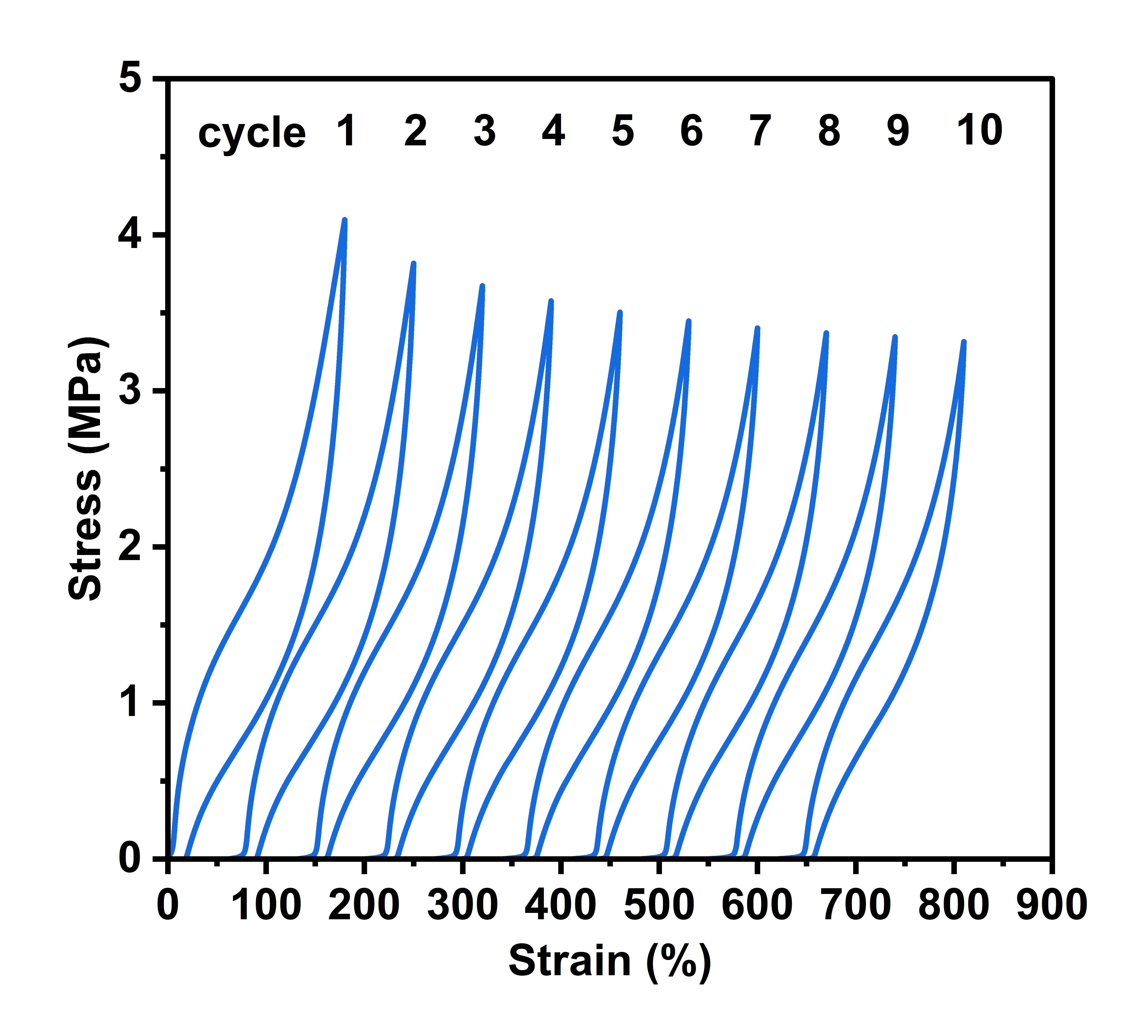


**(a)**

**(b)**


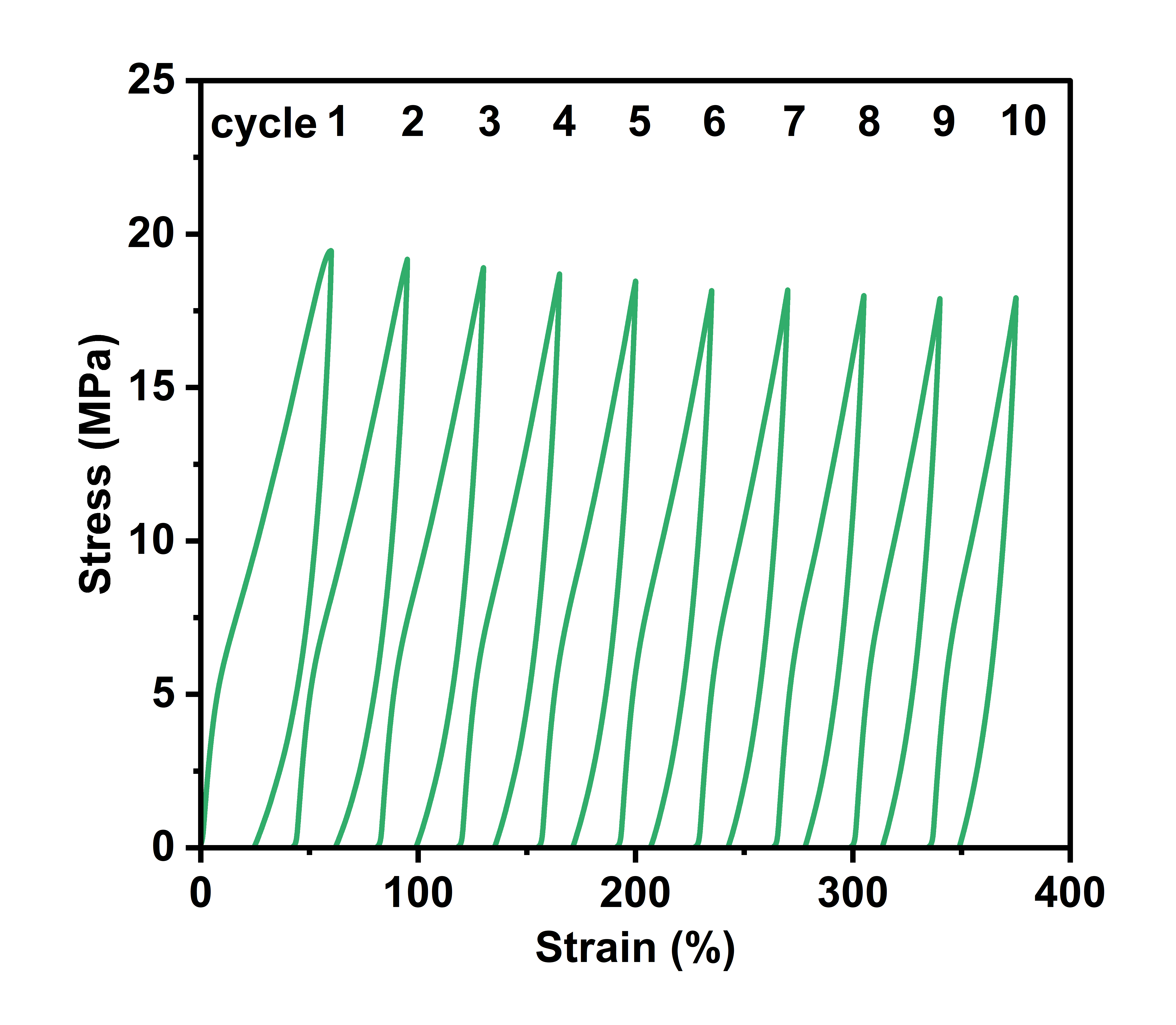


**(c)**

**P-2**

**P-4**

**P-5**

# Figure S20. Cyclic tensile testing of (a) P-2, (b) P-4, and (c) P-5 to 200% strain. 10 cycles shown after pre-processing after stretching to 100% strain (offset by 50% for clarity).


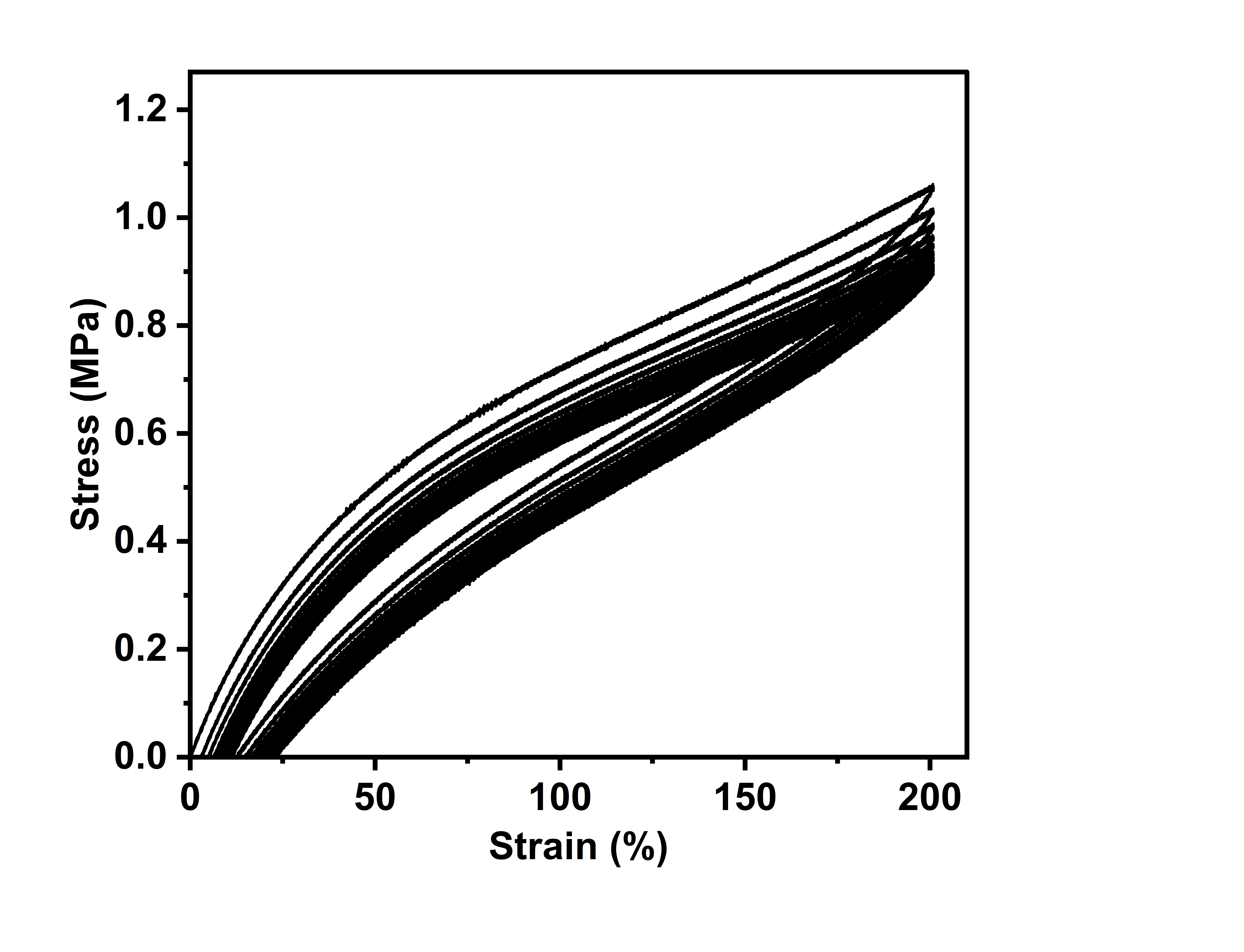

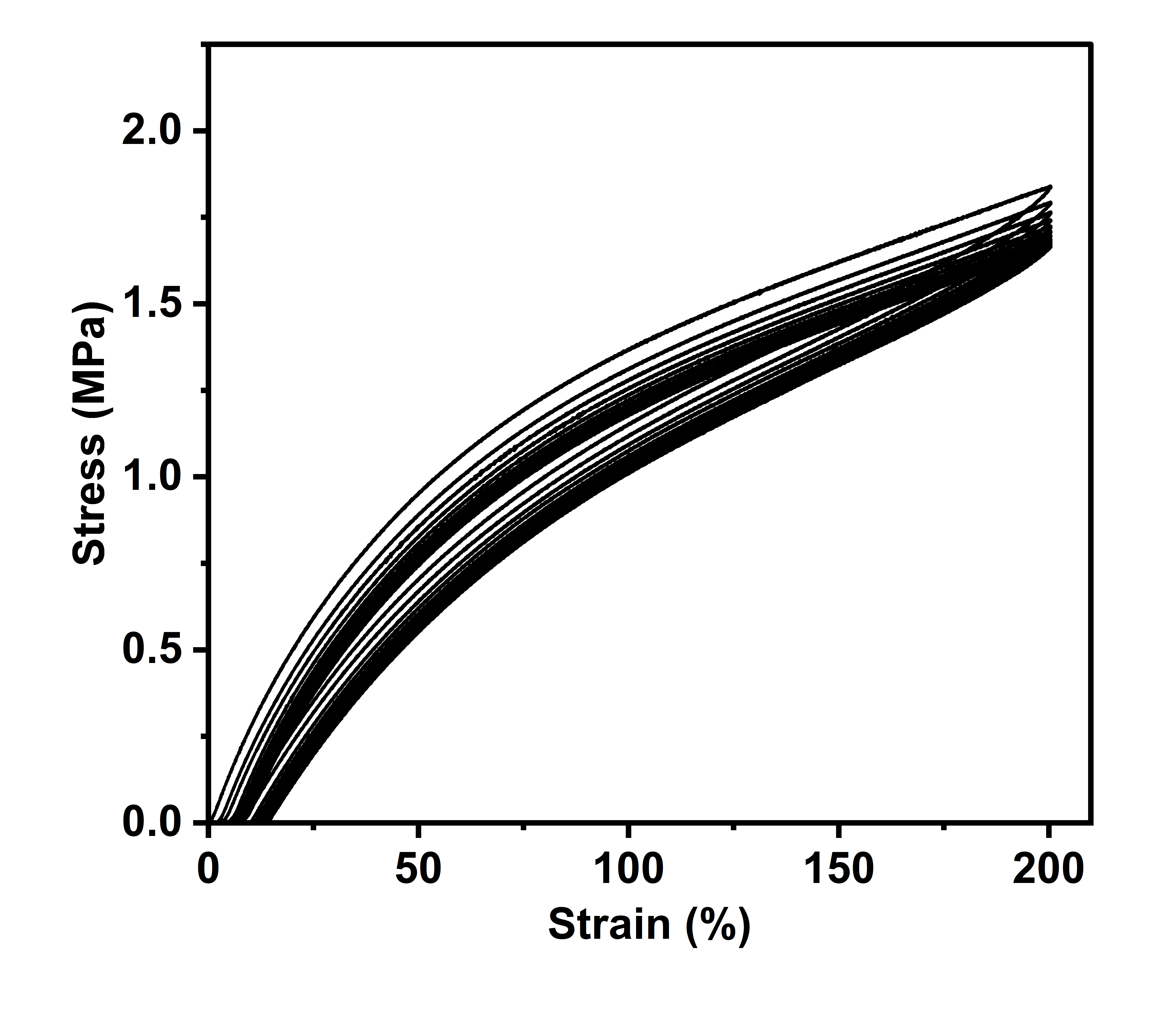


**(a)**

**(b)**


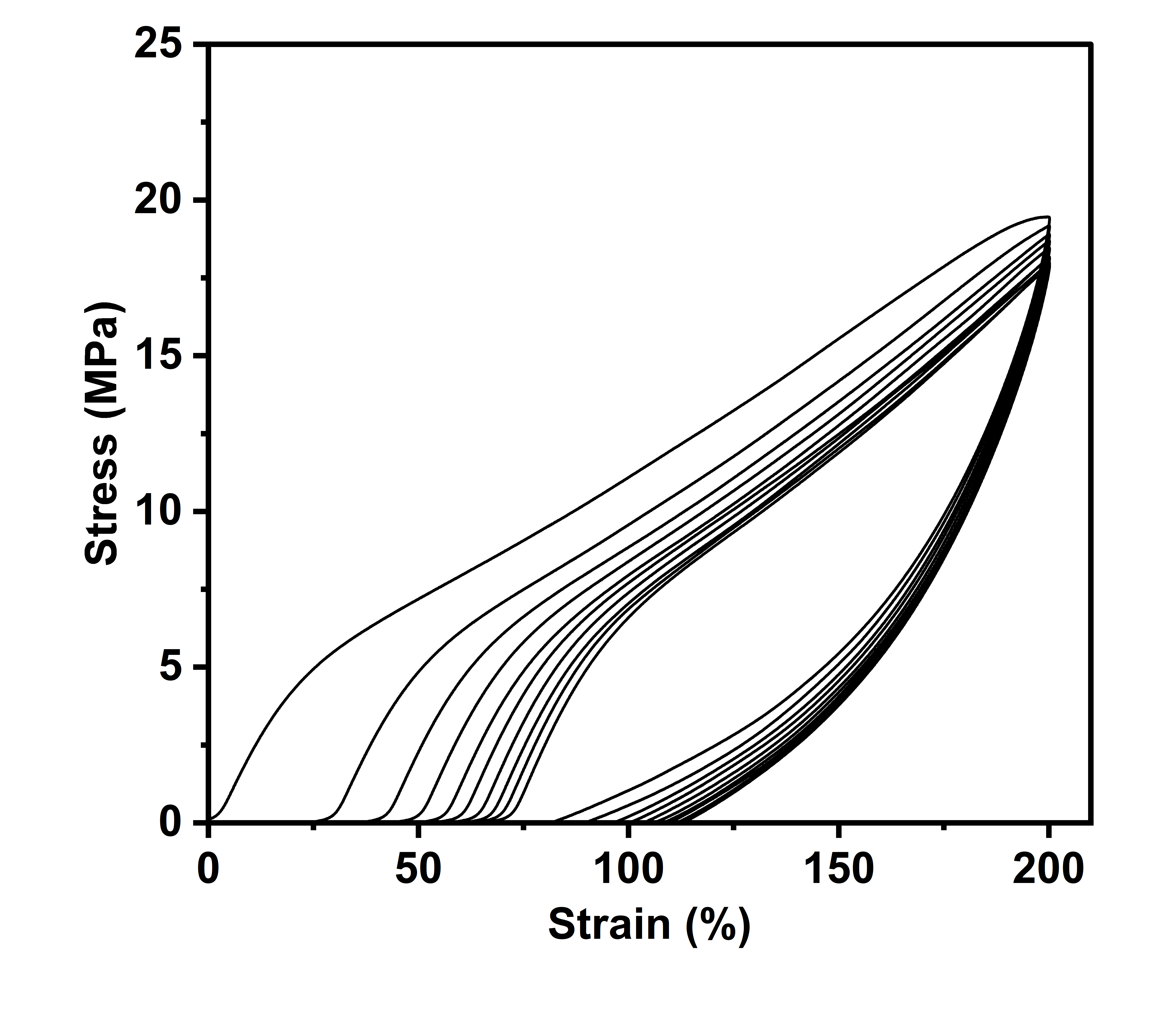

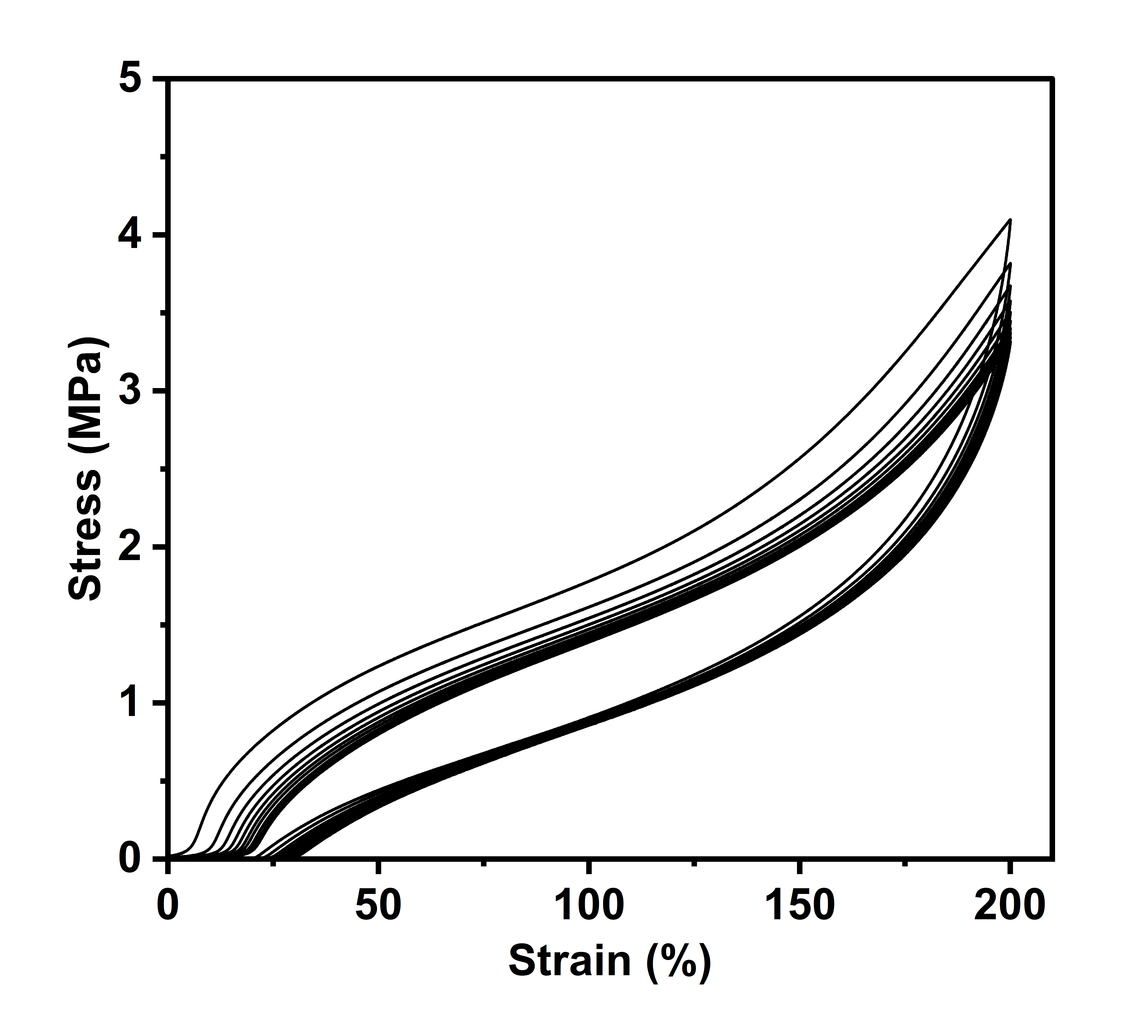


**(d)**

**(c)**

# Figure S21. Cyclic tensile testing of (a) P-2, (b) P-3, (c) P-5, and (d) P-5 to 200% strain. 10 cycles shown after pre-processing after stretching to 100% strain.

# Table S4. Summary of elastic recovery (ER), resilience, and residual strain (ε_R_) for all samples

| **Sample** | **PCL:PDL (wt%)*^a^*** | **ER** | **Resilience** | **ε_R_** |
| --- | --- | --- | --- | --- |
| **P-2** | 23 : 77 | 94.9 ± 4.7 | 76.0 ± 1.9 | 8.5 ± 4.4 |
| **P-3** | 48 : 52 | 96.6 ± 3.7 | 85.4 ± 1.5 | 4.6 ± 0.3 |
| **P-4** | 70 : 30 | 92.8 ± 11.8 | 66.4 ± 3.1 | 10.2 ± 1.3 |
| **P-5** | 100 : 0 | 62.4 ± 14.6 | 58.4 ± 13.0 | 15.2 ± 3.3 |
| **Septon™ (SEBS)** | - | 95.6 ± 0.7 | 85.8 ± 2.4 | 6.6 ± 1.5 |
| **Estane® (TPU)** | - | 92.8 ± 1.2 | 61.5 ± 3.6 | 11.0 ± 2.4 |

*^a^*Determined from ^1^H NMR spectrum of puriﬁed samples

# Figure S22. Samples P-3 and P-4 before and after tensile testing showing transition from transparent to opaque.


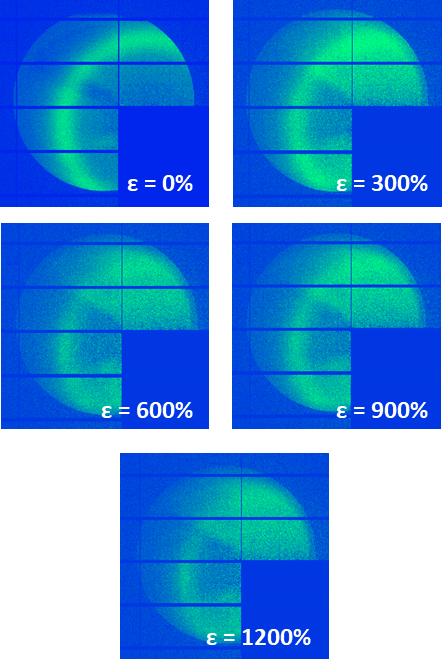


# Figure S23. Evolution of 2D-WAXS patterns of P-1 recorded at increasing % strain (ε).


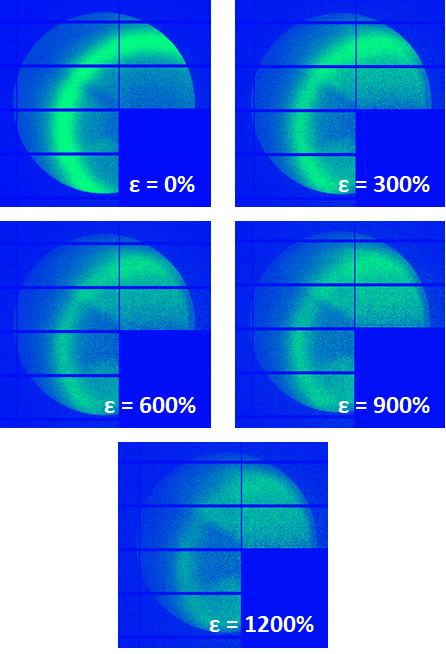


# Figure S24. Evolution of 2D-WAXS patterns of P-2 recorded at increasing % strain (ε).

# Figure S25. Evolution of 2D-WAXS patterns of P-4 recorded at increasing % strain (ε) and after recovery to 0% strain.


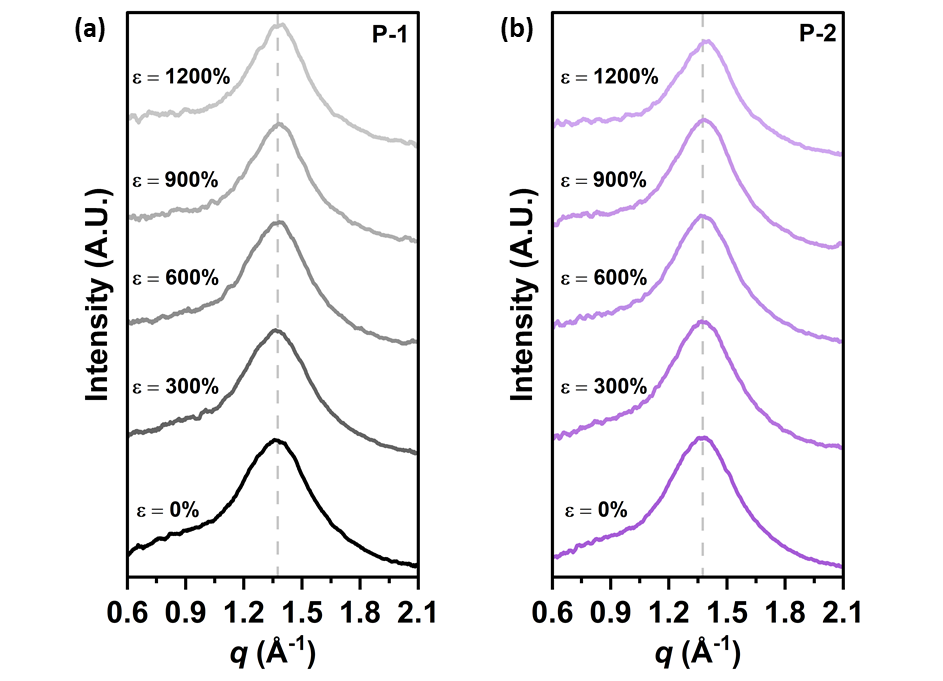


# Figure S26. 1D-WAXS intensity profiles at increasing % strain (ε) and after recovery to 0% strain for a) P-1 and b) P-2.


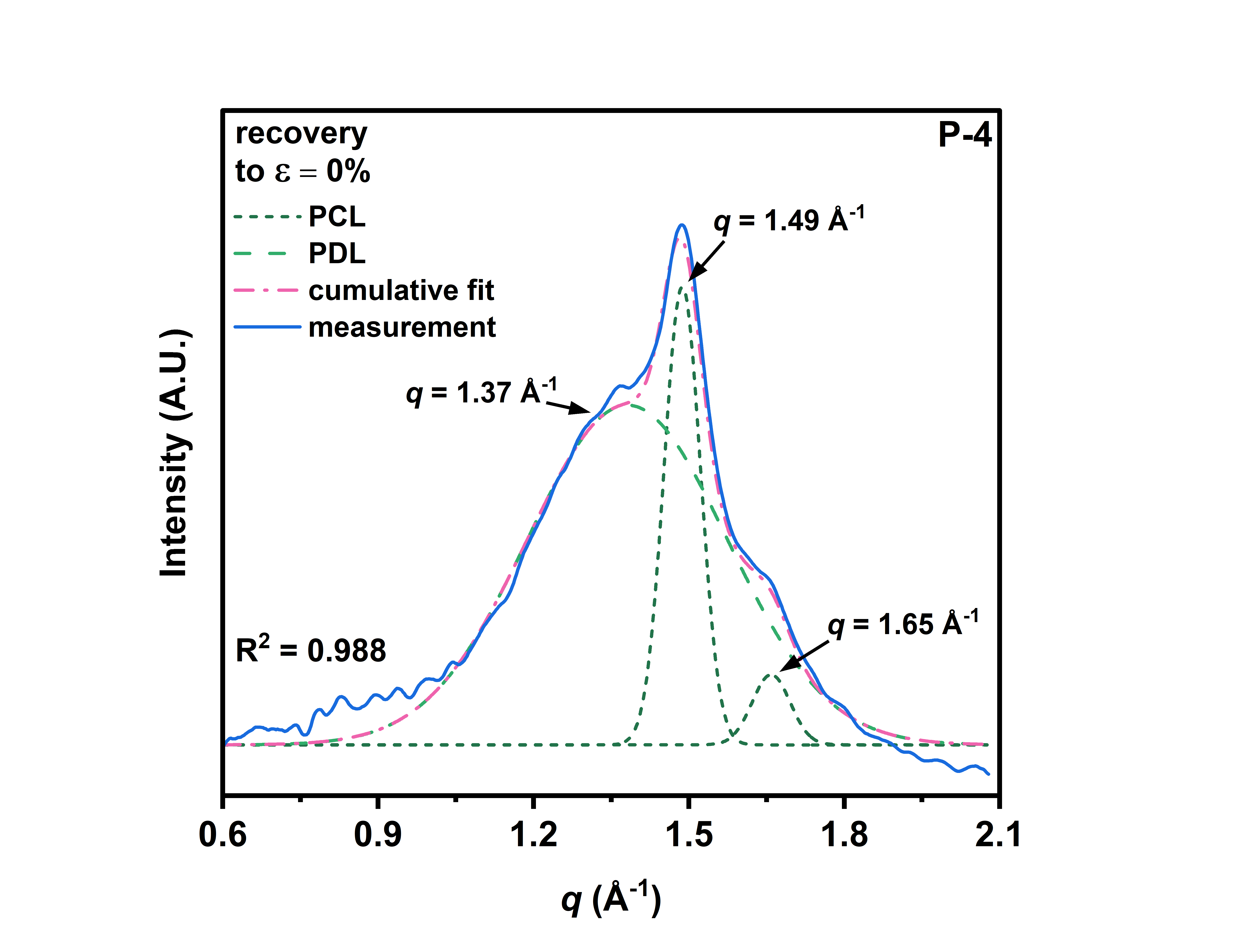


# Figure S27. Deconvoluted 1D-WAXS intensity profile of P-4 after recovery to 0% strain.


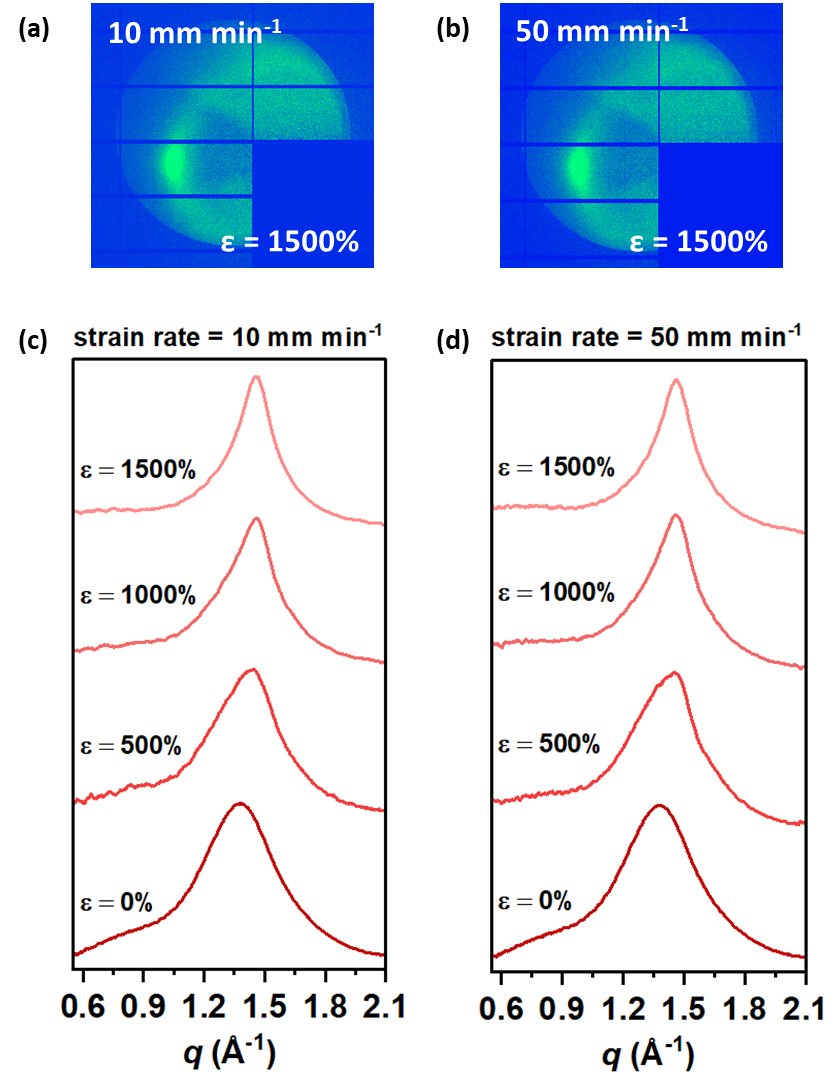


# Figure S28. 2D-WAXS pattern of P-3 recorded at 1500% strain after extension at (a) 10 mm min^-1^ strain rate and (b) 50 mm min^-1^ strain rate. 1D-WAXS intensity profiles at increasing % strain (ε) recorded at (c) 10 mm min^-1^ strain rate and (d) 50 mm min^-1^ strain rate. When increasing strain rate from 10 mm min^-1^ to 50 mm min^-1^, the ε_onset_ of strain-induced crystallization remains the same which suggests faster rate of crystallization during stretching at higher strain rate. The extent and reversibility of the SIC has no dependence on strain rate. This type of behavior is typical in materials exhibiting SIC.^[6]^


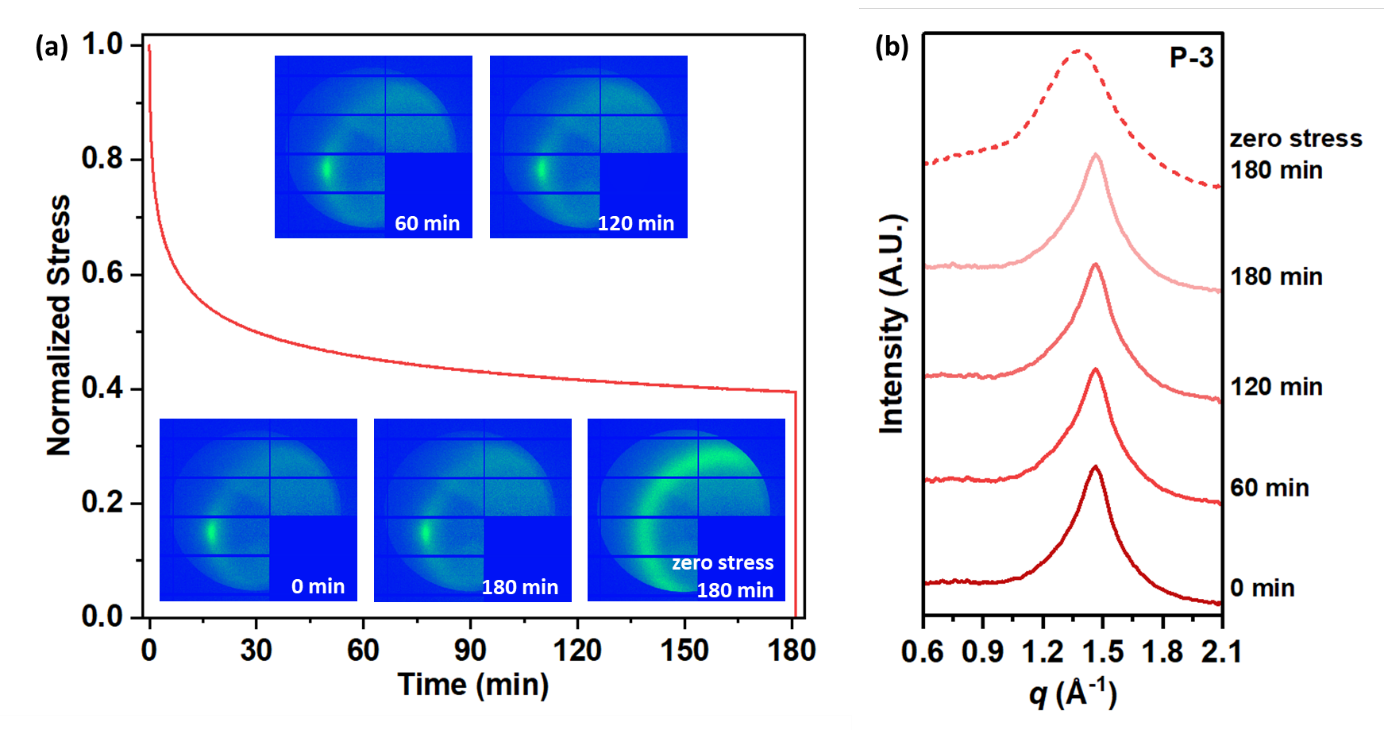


# Figure S29. (a) 2D-WAXS profiles taken during the stress relaxation of P-3 extended to 1500% for 3 h. b) 1D-WAXS intensity profiles at increasing P-3 at each time point and after recovery to zero stress.


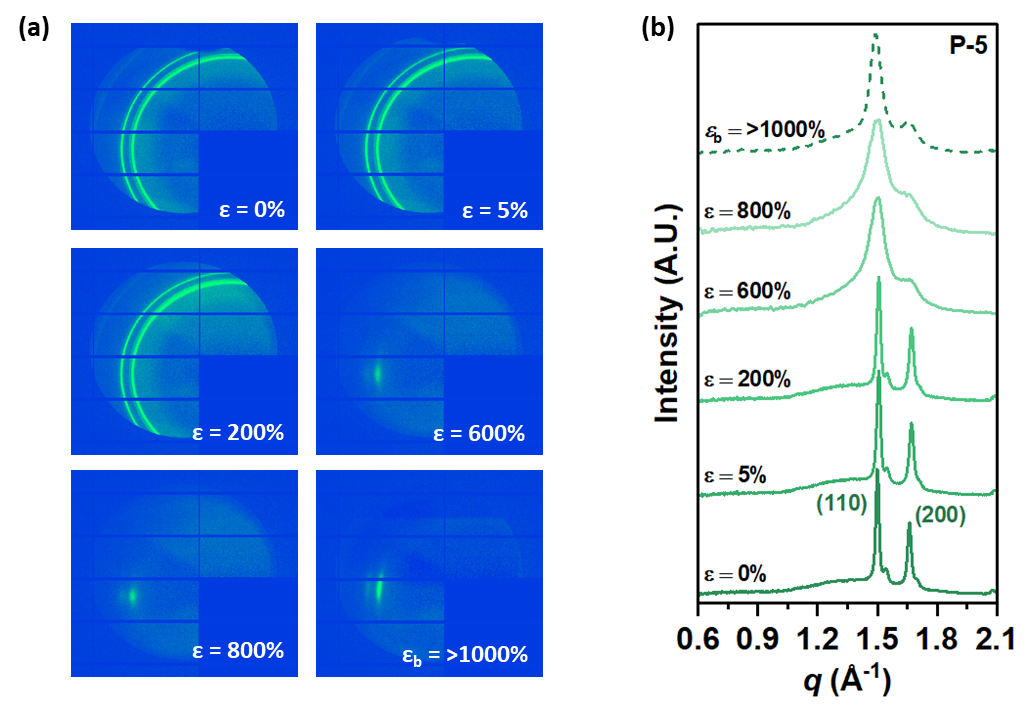


# Figure S30. (a) Evolution of 2D-WAXS patterns of P-5 recorded at increasing % strain (ε) and at break (ε_b_). (b) 1D-WAXS intensity profiles at increasing % strain (ε) and at break (ε_b_).


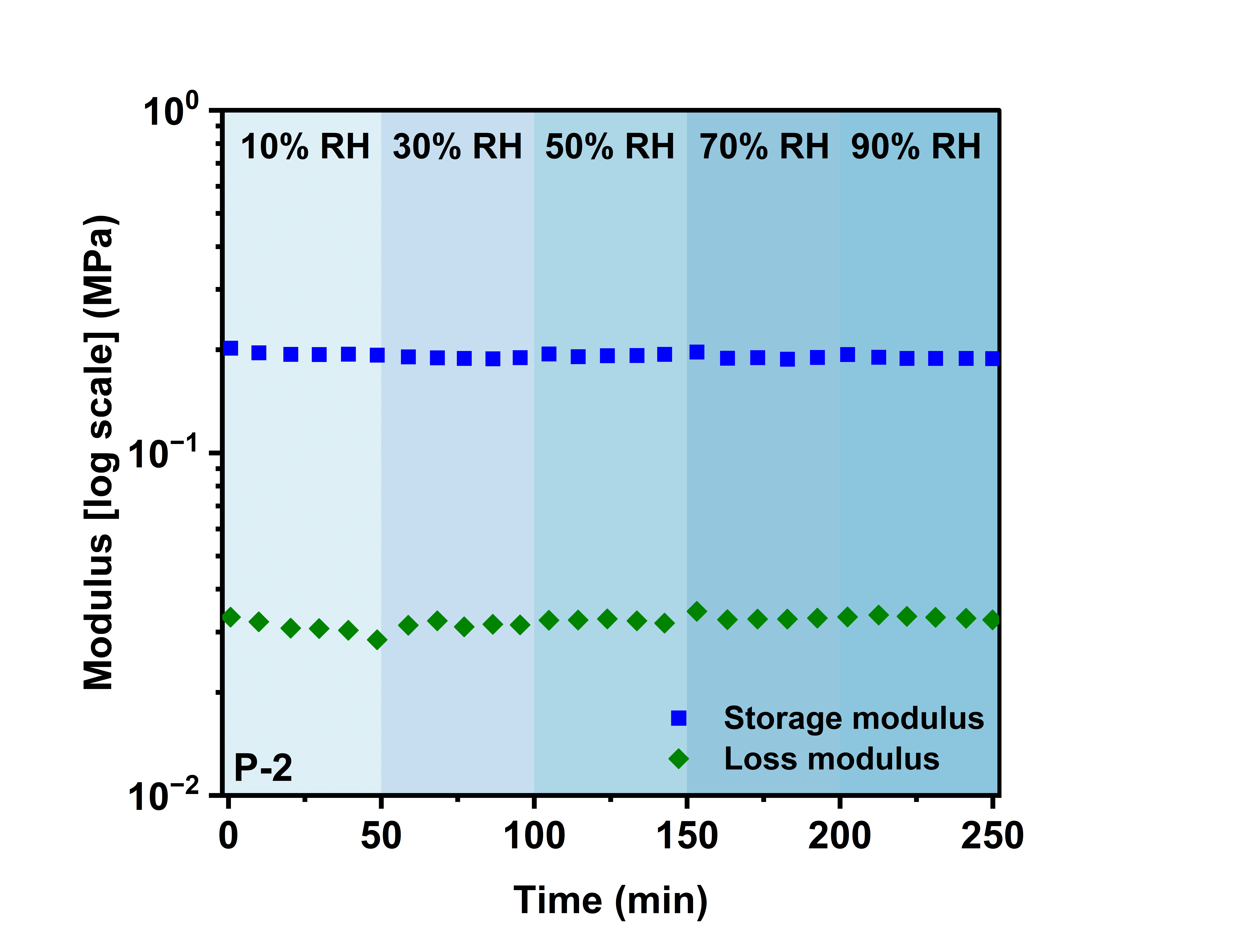

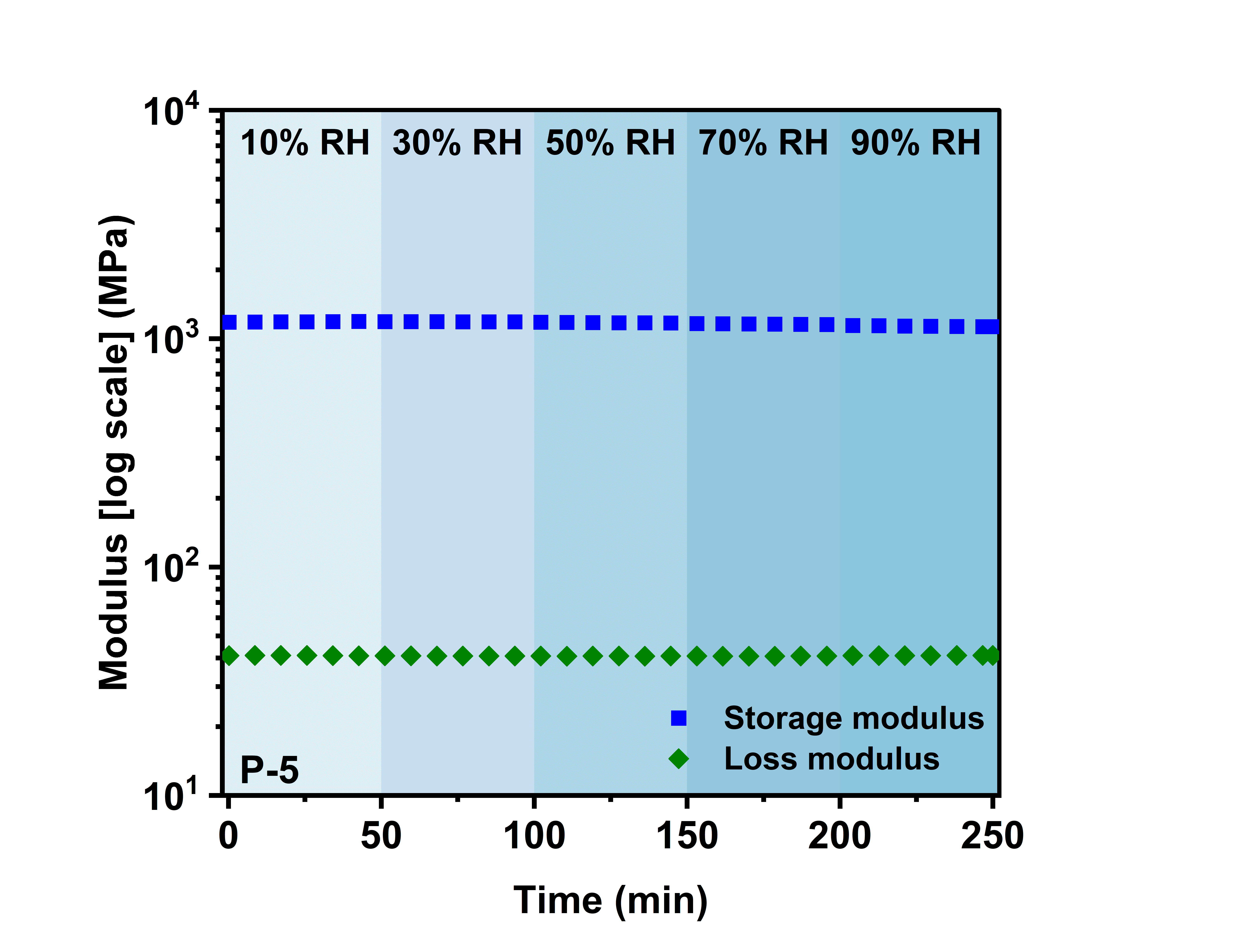

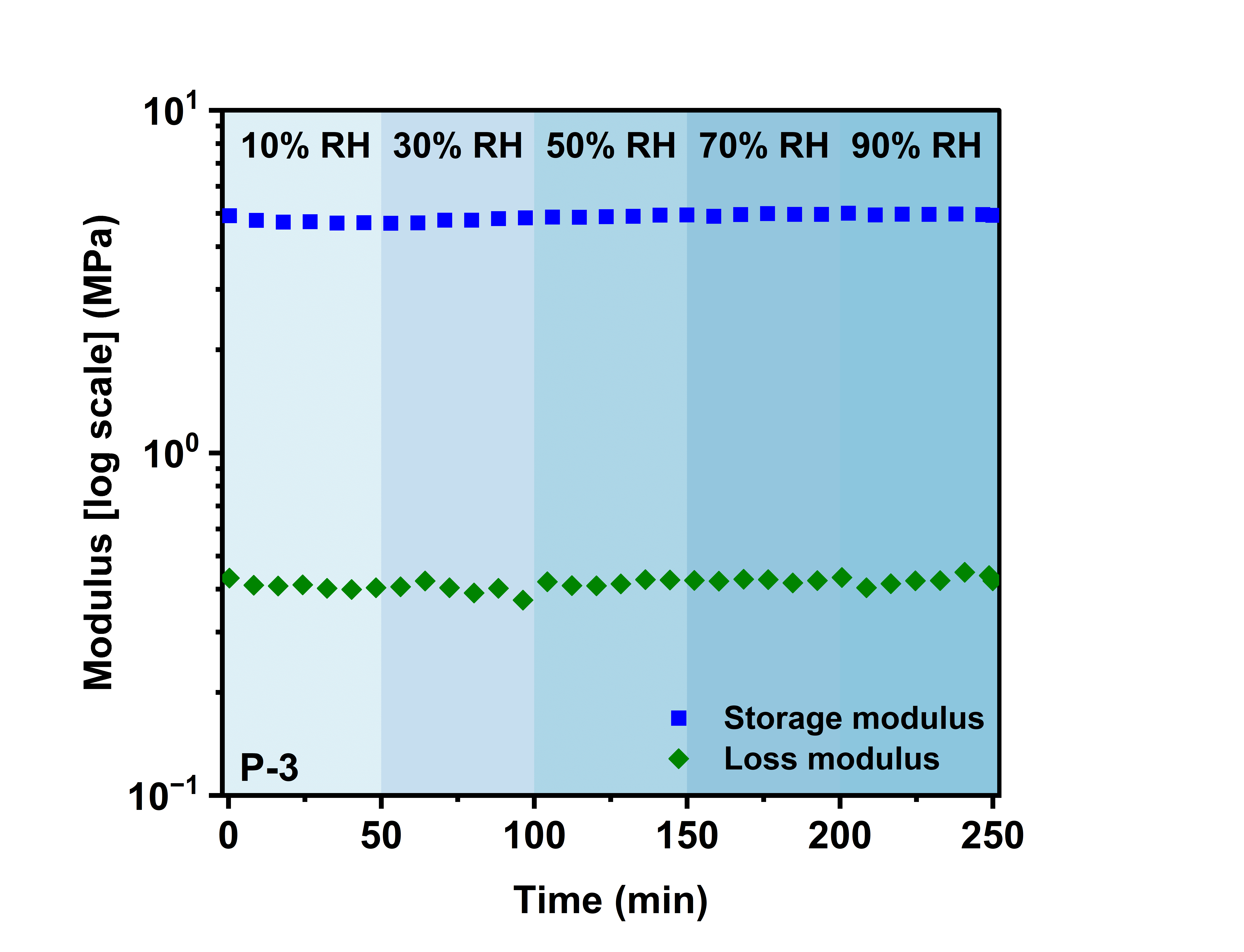




**(a)**

**(b)**

**(c)**

**(d)**

# Figure S31. DMA relative humidity sweeps for (a) P-2, (b) P-3, (c) P-4, and (d) P-5.


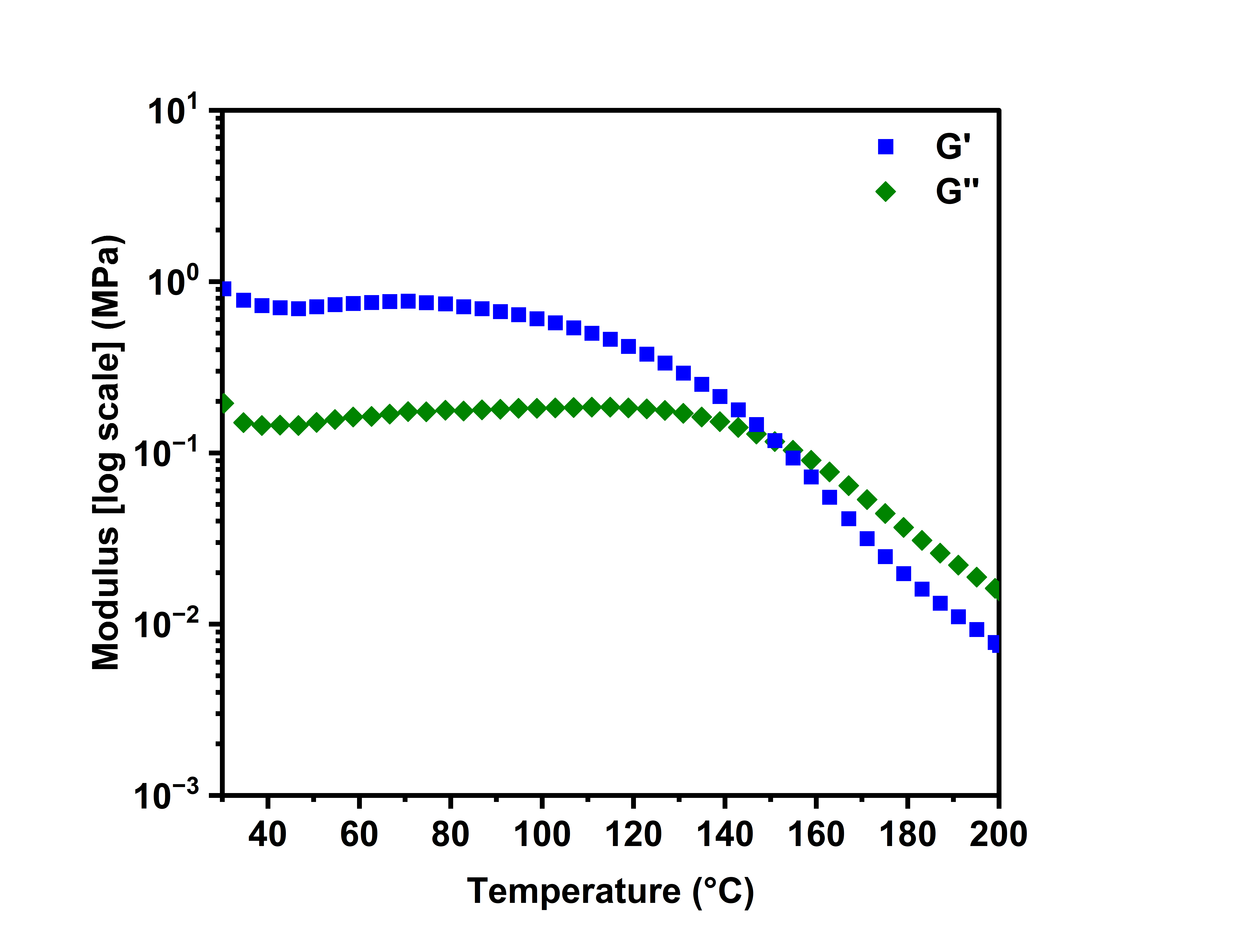


# Figure S32. Rheological temperature sweep of P-3 where the G’ to G’’ crossover occurred at 149 °C.


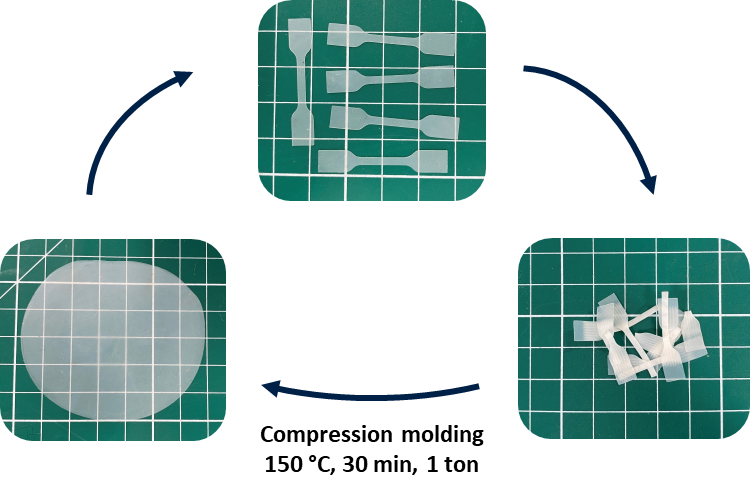


# Figure S33. Schematic of mechanical recycling by compression remolding.

# Table S5. Summary of TPE properties.

| **Sample** | **PCL:PDL*^a^* (wt%)** | ***T*_g_ (°C)*^b^*** | ***E*_y_ (MPa)*^c^*** | **σ (MPa) *^d^*** | **ε_b_ (%)*^e^*** | ***U*_T_**  **(MJ m^-3^) *^f^*** | **ER (%)*^g^*** |
| --- | --- | --- | --- | --- | --- | --- | --- |
| **P-1** | 0 : 100 | -42, 146 | 5.0 ± 0.7 | 6.5 ± 0.2 | 1097 ± 37 | 40 ± 2 | 95 ± 5 |
| **P-2** | 23 : 77 | -55, 143 | 2.1 ± 0.2 | 3.0 ± 0.3 | 1374 ± 39 | 31 ± 2 | 95± 5 |
| **P-3** | 48 : 52 | -57, 132 | 7.3 ± 2.0 | 38.1 ± 1.8 | 2422 ± 159 | 345 ± 36 | 97± 4 |
| **P-4** | 70 : 30 | -62, 120* | 130.6 ± 22.6 | 34.2 ± 2.5 | 1506 ± 64 | 247 ± 24 | 93± 12 |
| **P-5** | 100 : 0 | -62, 111 | 515.0 ± 60.8 | 46.2 ± 6.4 | 1156 ± 101 | 311 ± 61 | 62± 16 |
| **Septon™ (SEBS)^[7]^** | - | -45, 140 | 2.6 ± 0.5 | 34.0 ± 1.6 | 1743 ± | 198 ± 10 | 95.6 ± 0.7 |
| **Estane® (TPU)^[8]^** | - | -30, 110 | 3.1 ± 0.8 | 37.0 ± 3.2 | 1468 ± 195 | 273 ± 59 | 92.8 ± 1.2 |

*^a^*Determined from ^1^H NMR spectrum of puriﬁed samples. *^b^*Determined by DSC, second heating curve. *Upper glass transition temperature from peak in tan(δ) by DMTA. *^c^*Young’s modulus. *^d^*Tensile strength. *^e^*Strain at break. *^f^*Tensile toughness (area under the stress-strain curve). Mean values ± std. dev. from measurements conducted independently on five specimens. *^g^*Elastic recovery determined from cyclic tensile testing to 200% strain, mean values ± std. dev over ten hysteresis cycles.


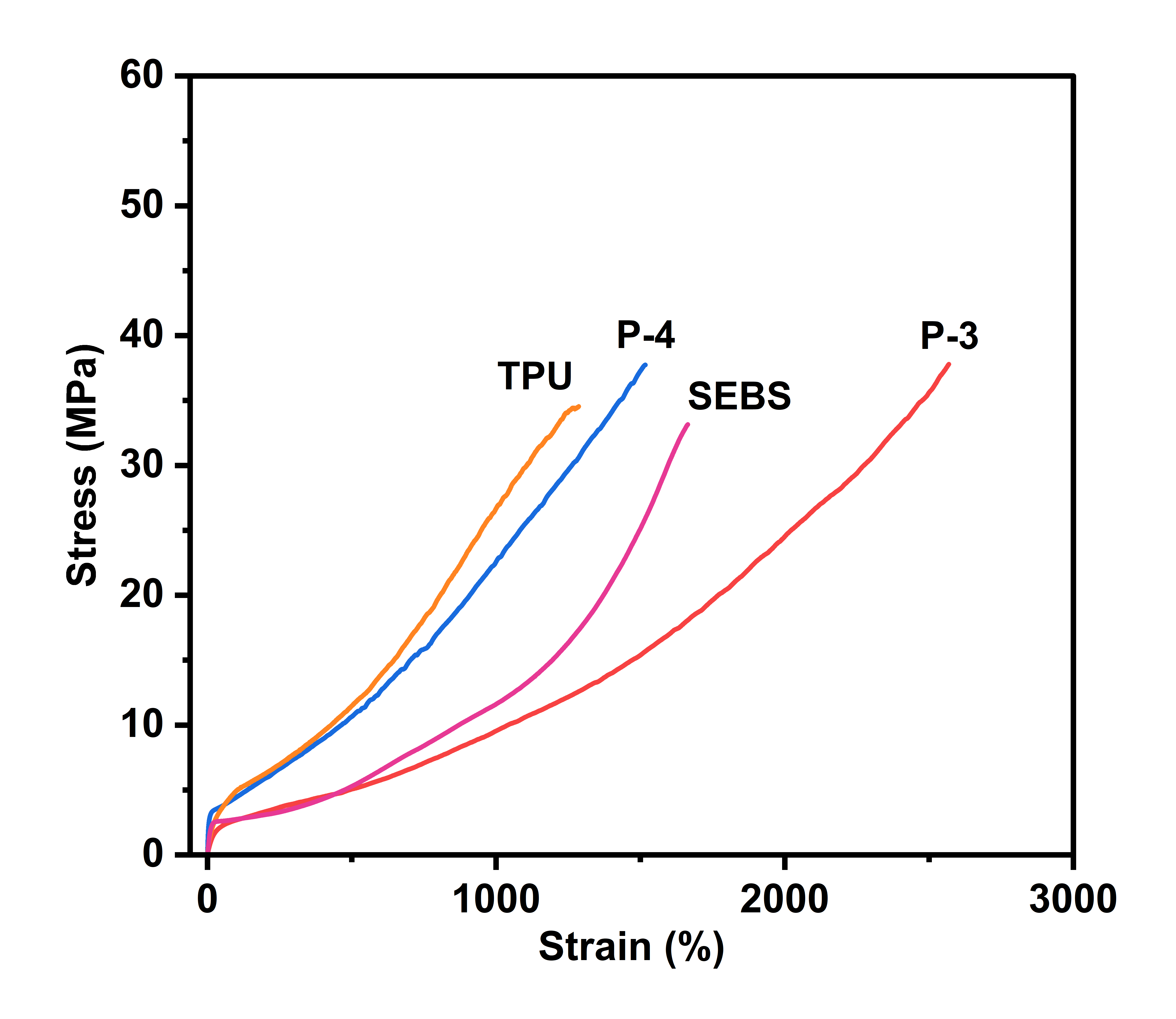


# Figure S34. Tensile stress-strain curves (representative of five repeats) comparing P-3 and P-4 to commercial SEBS and PU.


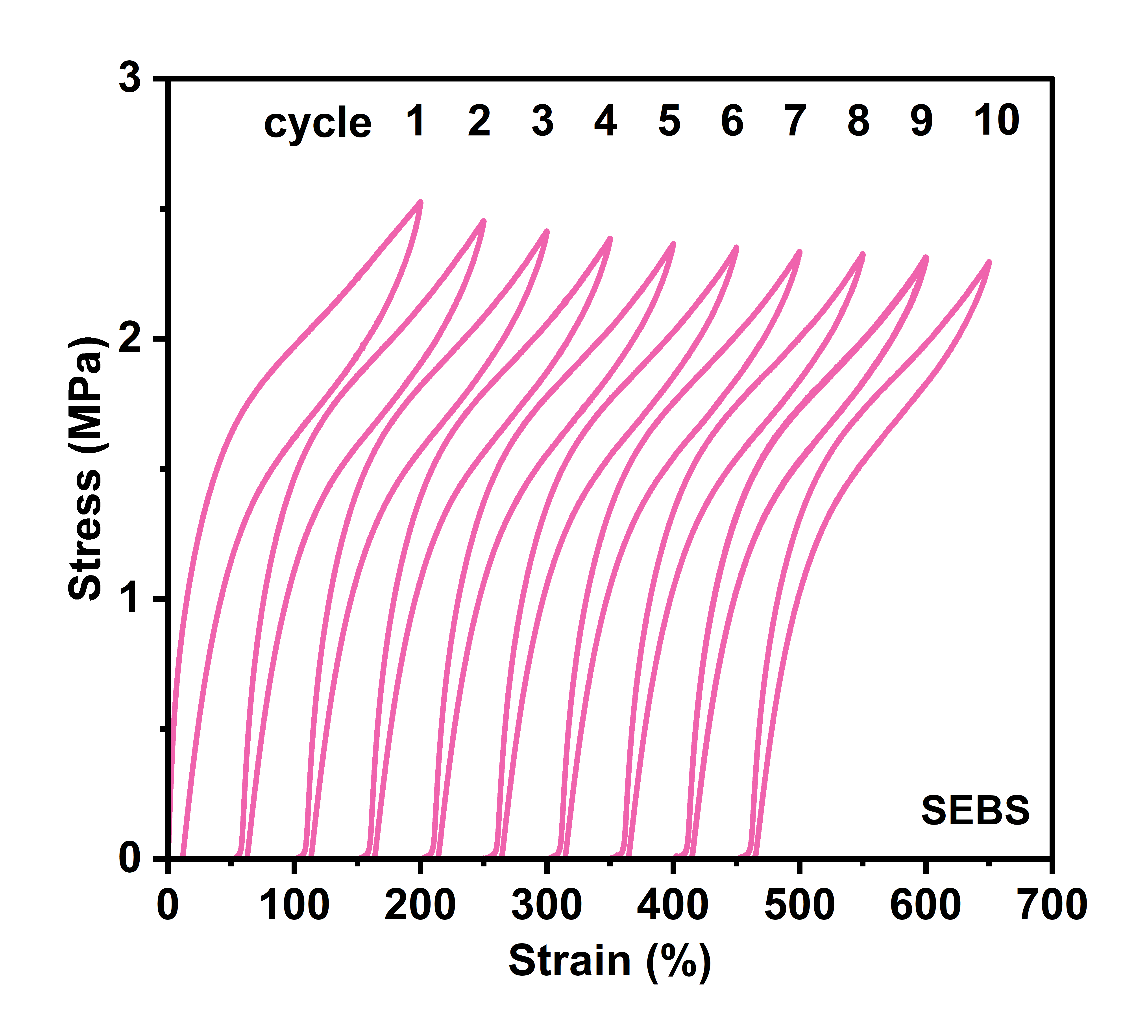


# Figure S35. Cyclic testing of SEBS to 200% strain (offset by 50% for clarity).


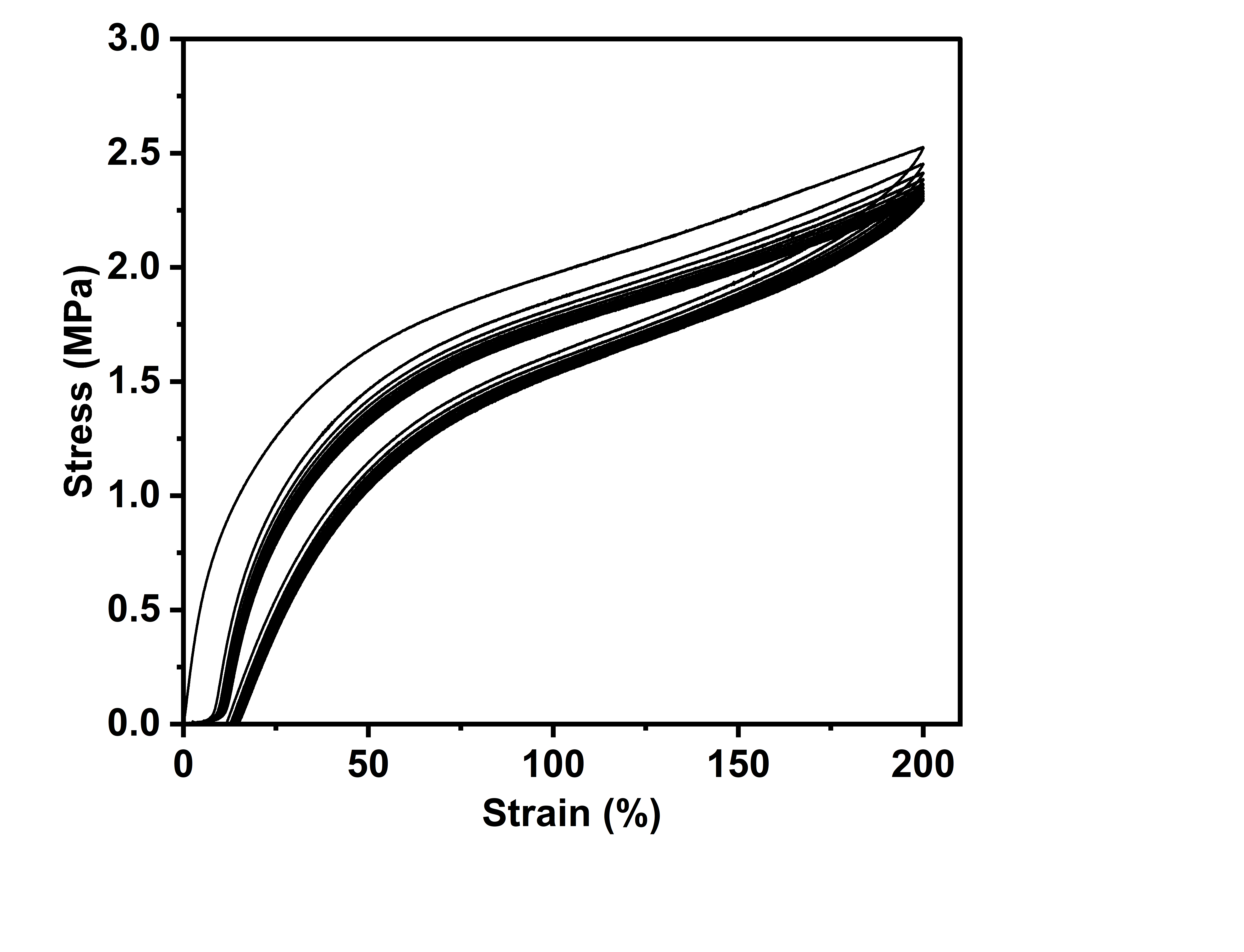


# Figure S36. Cyclic testing of SEBS to 200% strain.


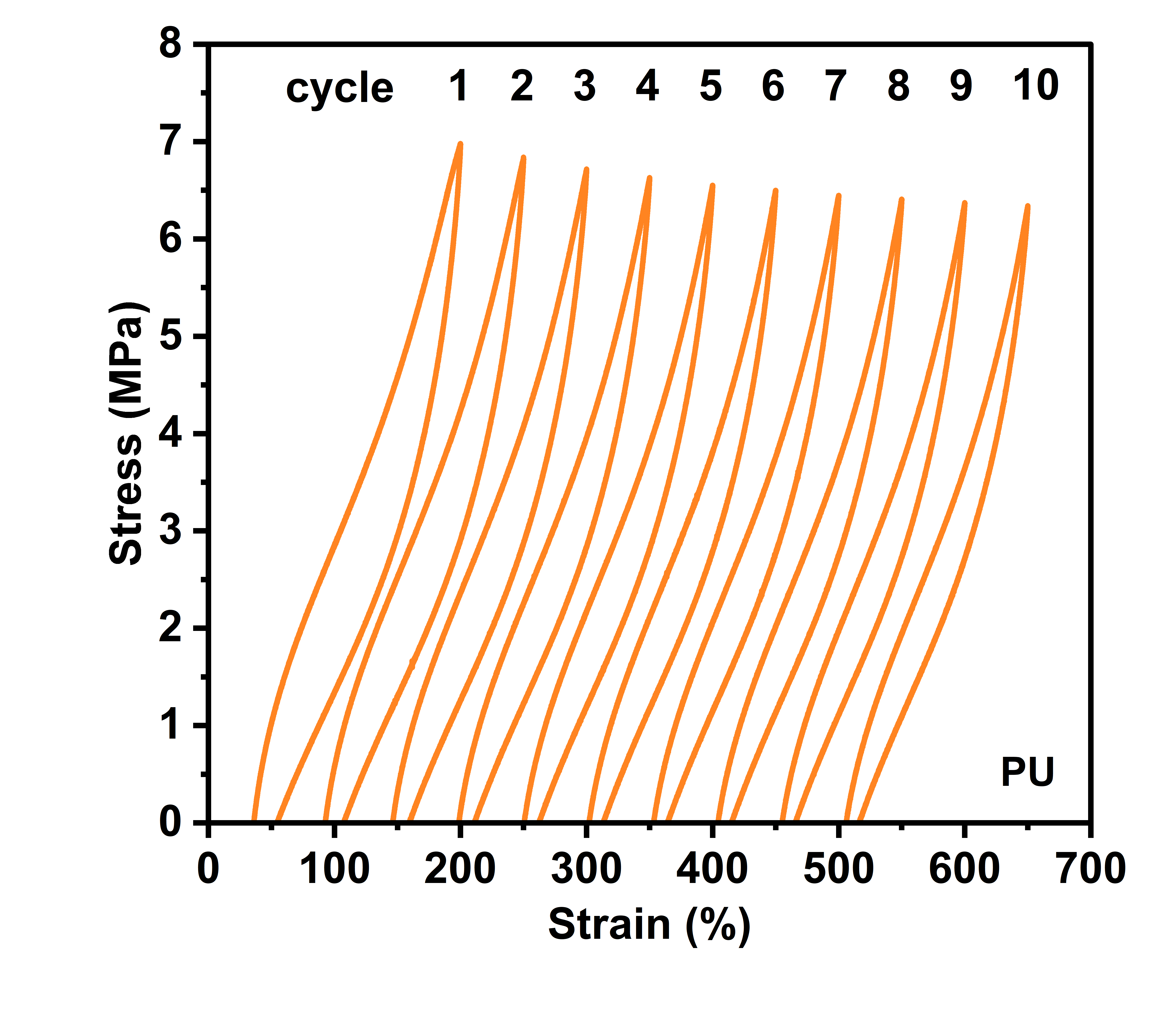


# Figure S37. Cyclic testing of PU to 200% strain (offset by 50% for clarity).


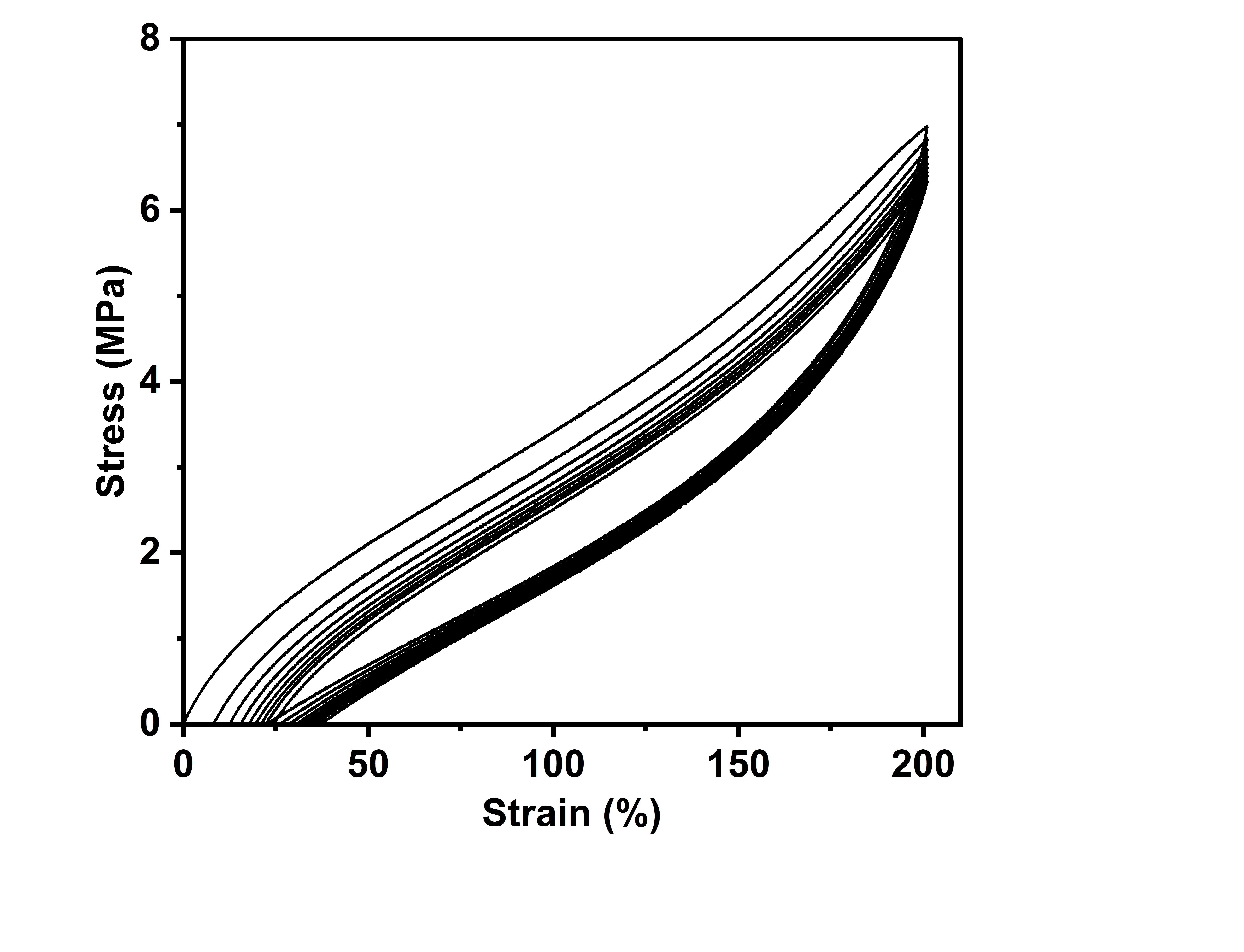


# Figure S38. Cyclic testing of PU to 200% strain.


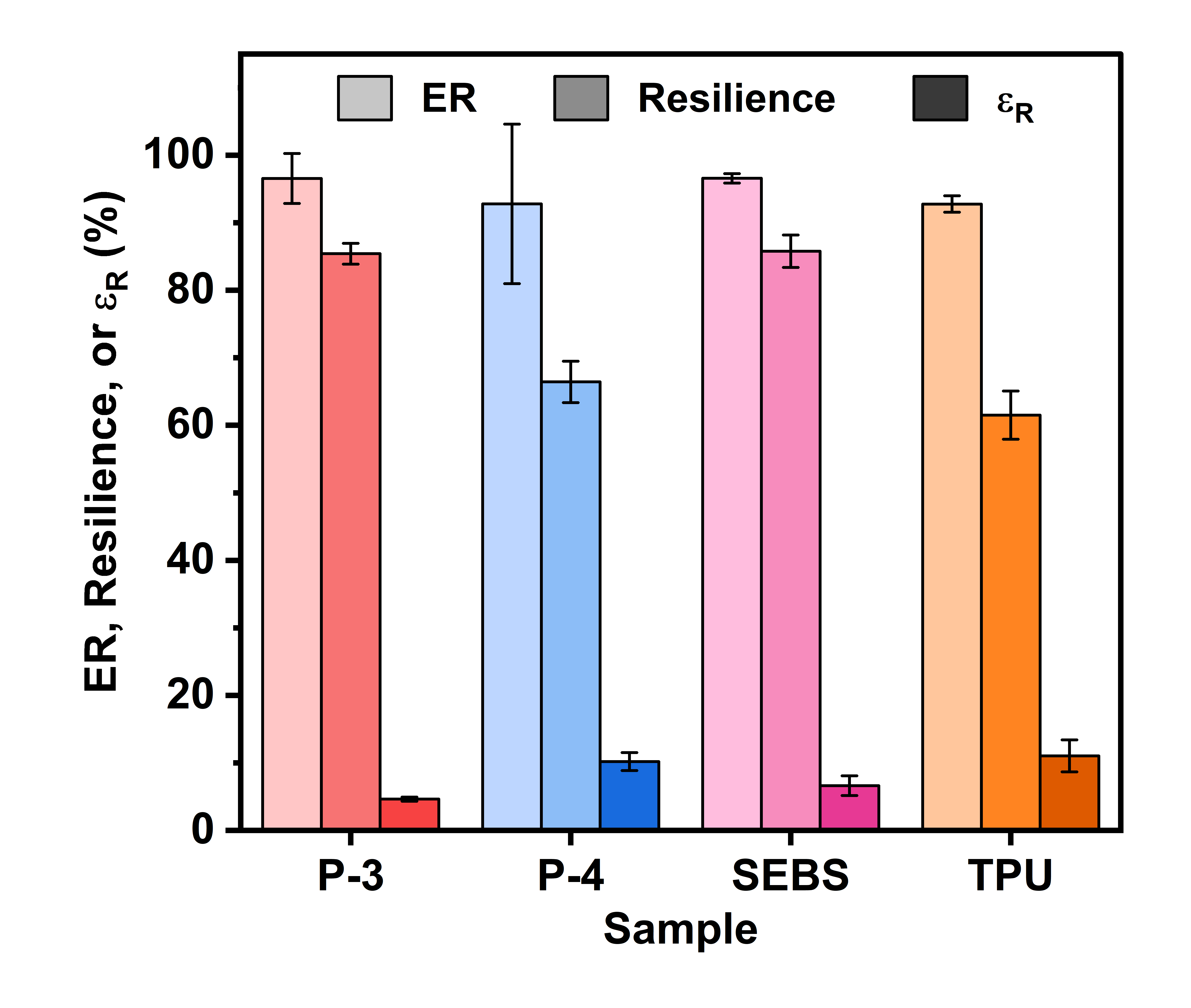


# Figure S39. Comparison of elastic recovery (ER), resilience, and residual strain (ε_R_) for P-3, P-4 , SEBS an PU.


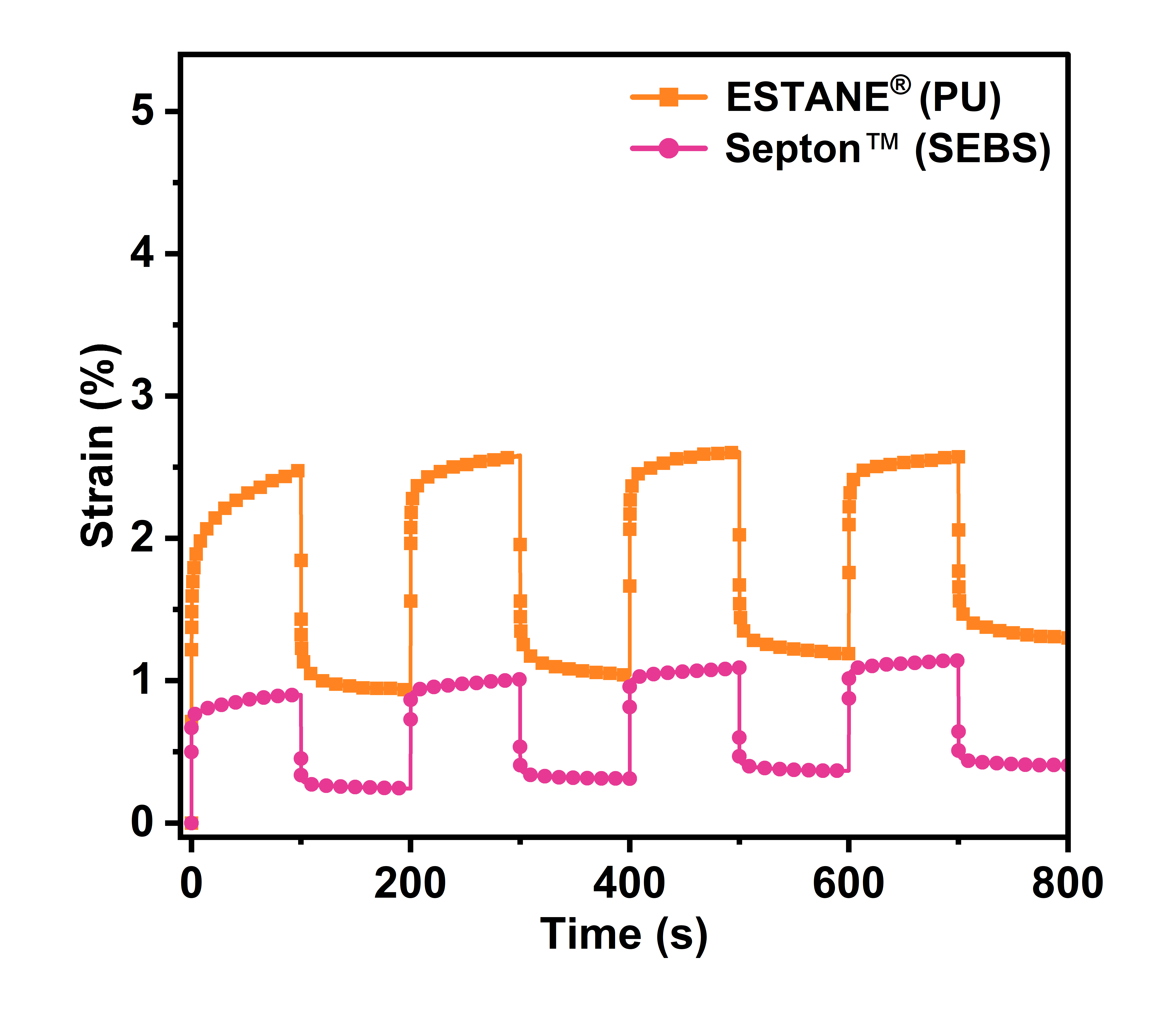


# Figure S40. Creep -recovery experiments commercial SEBS and PU.


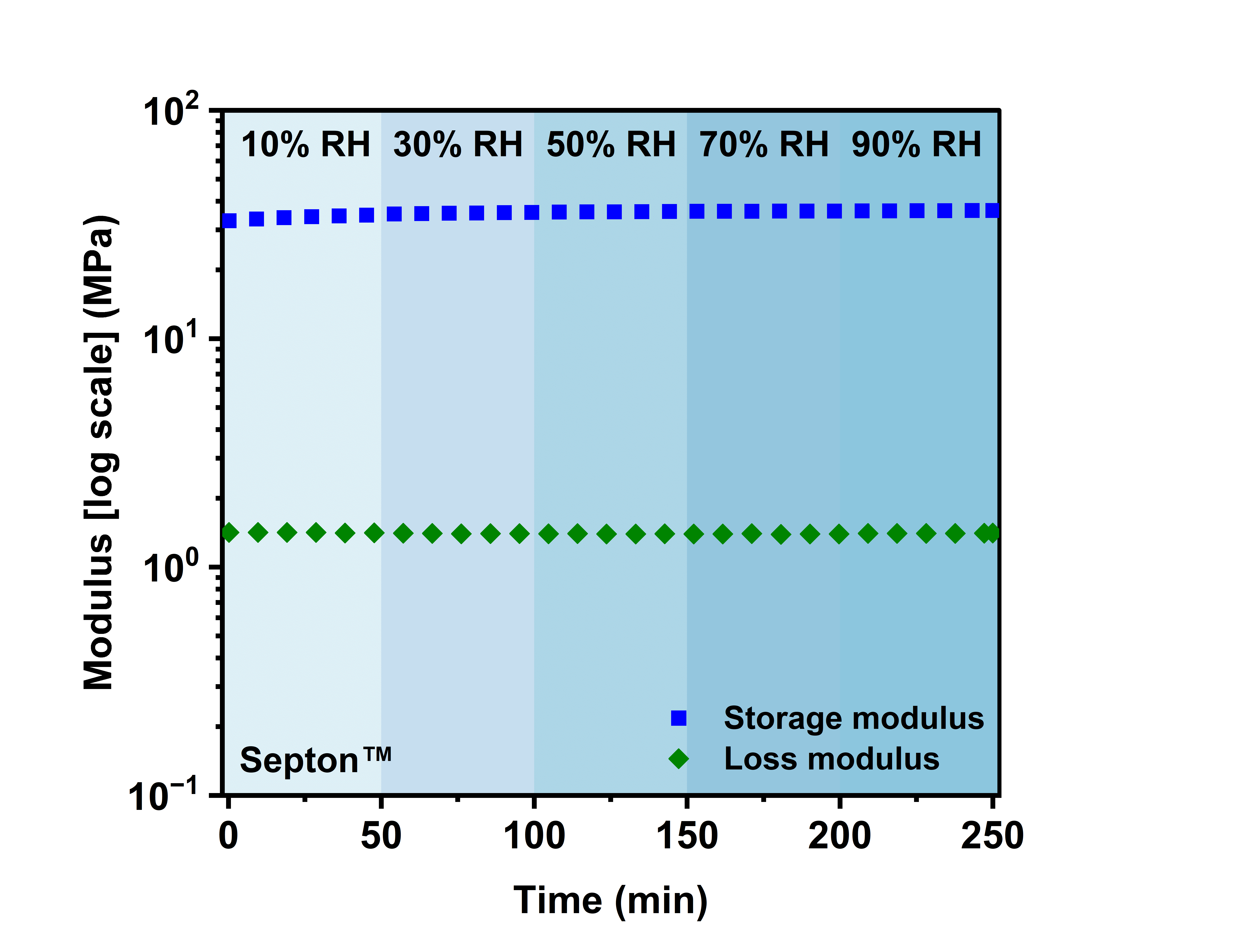

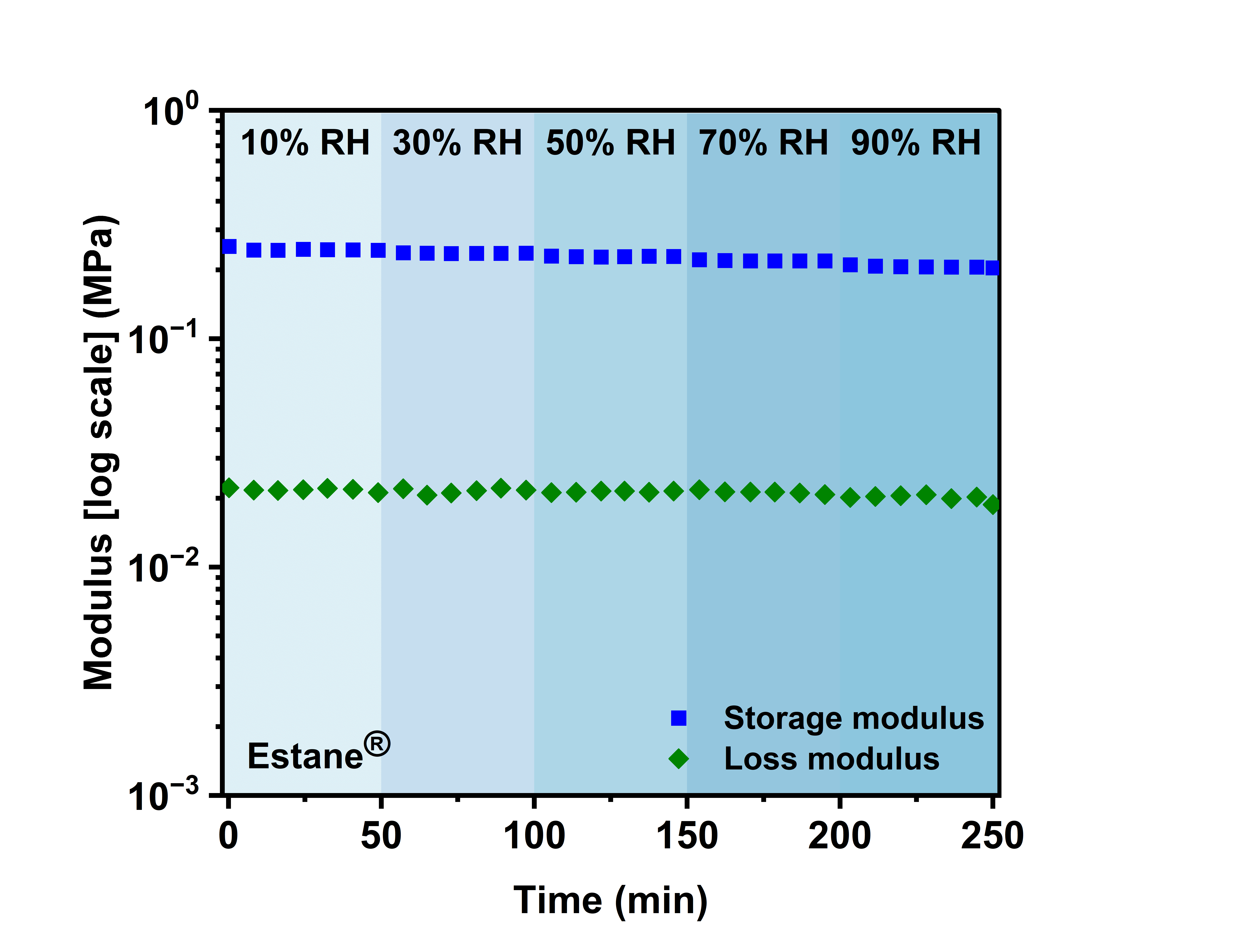


**(a)**

**(b)**

# Figure S41. DMA relative humidity sweeps for (a) commercial SEBS and (b) commercial PU.


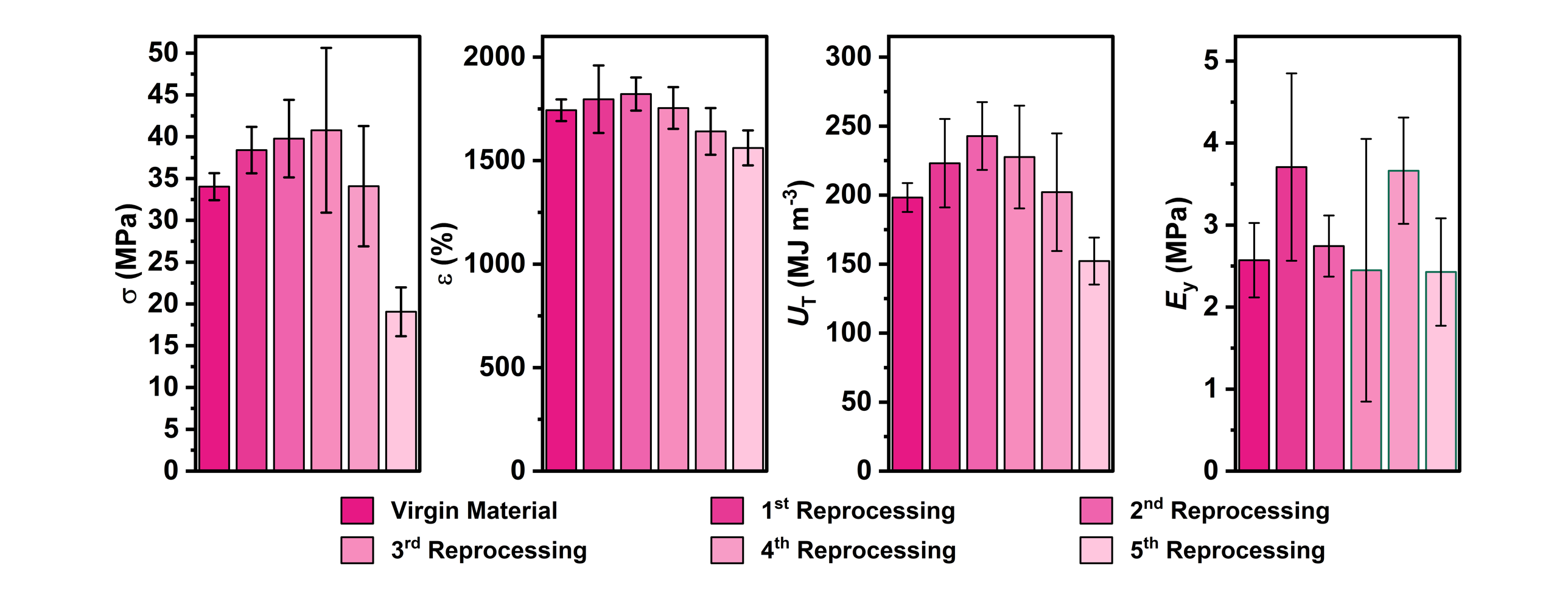


# Figure S42. a) Tensile strength, b) Strain at break, c) Tensile toughness, and d) Young’s Modulus for virgin commercial SEBS and after five mechanical reprocessing cycles. Mean values ±std. dev. from measurements conducted independently on five specimens.


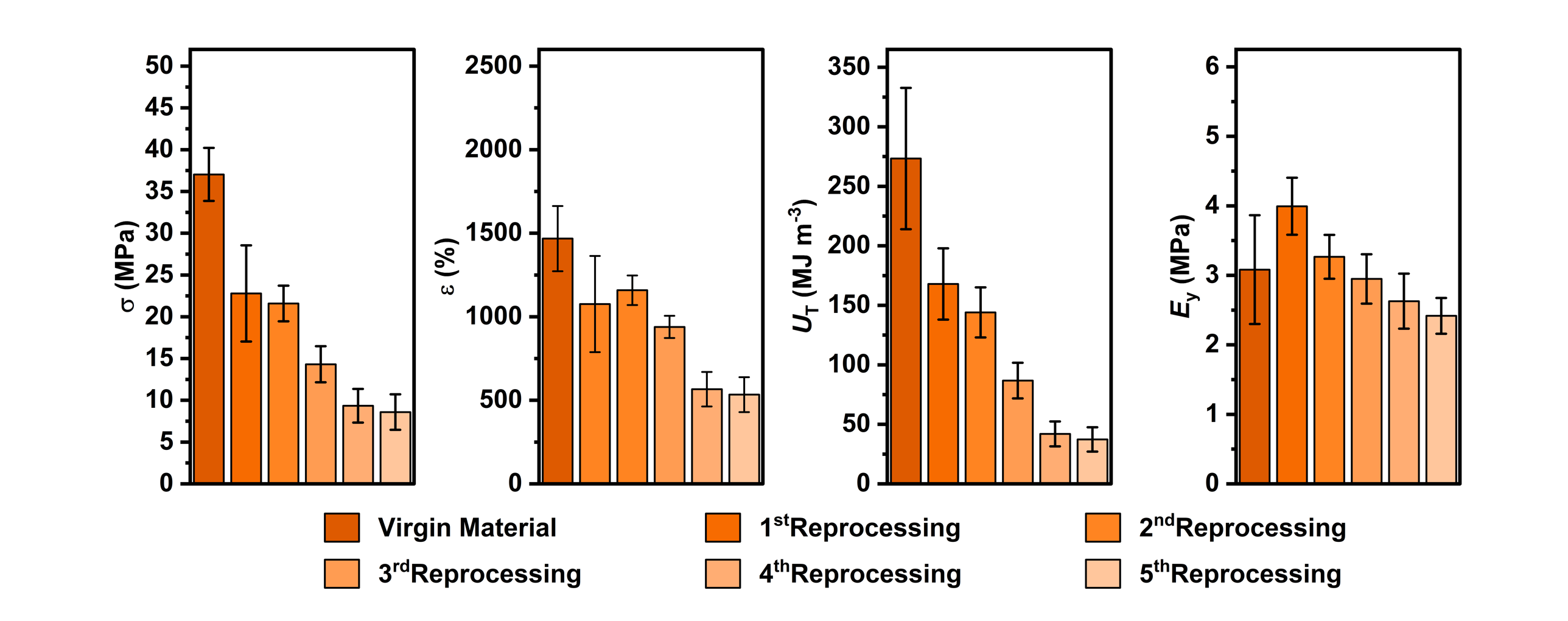


# Figure S43. a) Tensile strength, b) Strain at break, c) Tensile toughness, and d) Young’s Modulus for virgin commercial PUand after five mechanical reprocessing cycles. Mean values ±std. dev. from measurements conducted independently on five specimens.


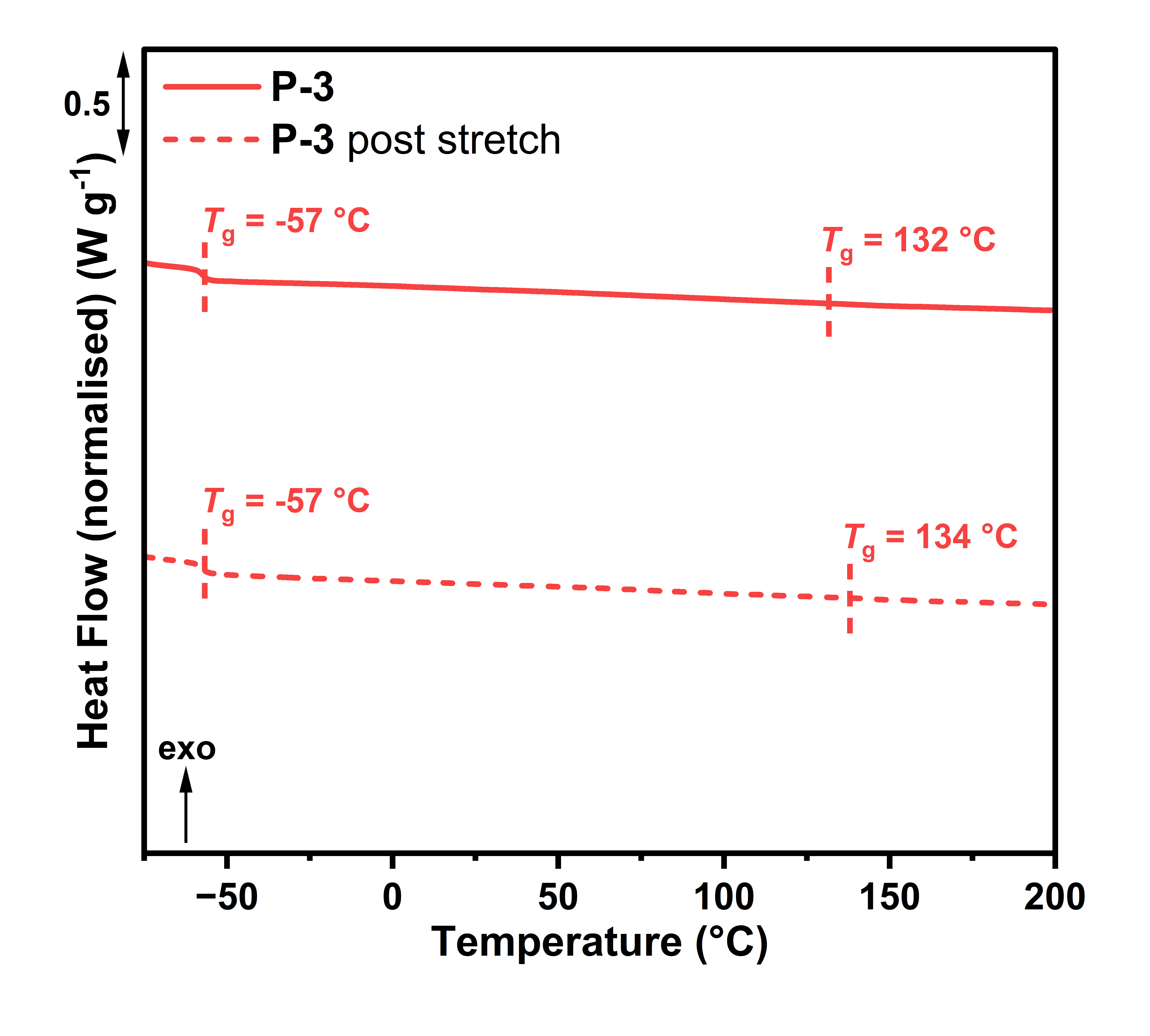


# Figure S44. DSC trace for P-3 and P-3 (dotted) measured immediately after tensile testing.

# References

[1] A. Spyros, D. S. Argyropoulos, R. H. Marchessault, *Macromolecules* **1997**, 30, 327.

[2] a) J. Filik, A. W. Ashton, P. C. Y. Chang, P. A. Chater, S. J. Day, M. Drakopoulos, M. W. Gerring, M. L. Hart, O. V. Magdysyuk, S. Michalik, A. Smith, C. C. Tang, N. J. Terrill, M. T. Wharmby, H. Wilhelm, *J. Appl. Cryst.* **2017**, 50, 959; b) B. R. Pauw, A. J. Smith, T. Snow, N. J. Terrill, A. F. Thunemann, *J. Appl. Cryst.* **2017**, 50, 1800; c) A. J. Smith, S. G. Alcock, L. S. Davidson, J. H. Emmins, J. C. Hiller Bardsley, P. Holloway, M. Malfois, A. R. Marshall, C. L. Pizzey, S. E. Rogers, O. Shebanova, T. Snow, J. P. Sutter, E. P. Williams, N. J. Terrill, *J. Synchrotron Rad.* **2021**, 28, 939.

[3] G. S. Sulley, G. L. Gregory, T. T. D. Chen, L. Peña Carrodeguas, G. Trott, A. Santmarti, K. Y. Lee, N. J. Terrill, C. K. Williams, *J. Am. Chem. Soc.* **2020**, 142, 4367.

[4] Q. P. Guo, G. Groeninckx, *Polymer* **2001**, 42, 8647.

[5] N. Kazemi, T. A. Duever, A. Penlidis, *Macromol. React. Eng.* **2011**, 5, 385.

[6] a) N. Candau, R. Laghmach, L. Chazeau, J.-M. Chenal, C. Gauthier, T. Biben, E. Munch, *Eur. Polym. J.* **2015**, 64, 244; b) P. Sotta, P.-A. Albouy, *Macromolecules* **2020**, 53, 3097; c) P.-A. Albouy, P. Sotta, *Macromolecules* **2020**, 53, 992.

[7] Kuraray, SEPTON™, HYBRAR™ Thermoplastic Elastomers Technical Information, <https://kuraray.com/uploads/5d40f79974d6b/Septon_and_Hybrar_Technical_Information.pdf>, July, **2024**

[8] Lubrizol, ESTANE® 58213 TPU, <https://www.lubrizol.com/-/media/Lubrizol/Engineered-Polymers/Documents/Engineered-Polymer-TDS/ESTANE-58213.pdf>, 2024, **July**
